# LL37-driven mast cell degranulation and inflammation in rosacea via TLR2/JAK2/STAT3 axis

**DOI:** 10.3389/fimmu.2025.1672021

**Published:** 2025-11-26

**Authors:** Huiping Fan, Rui Sun, Qingsong Ma, Xiaojin Li, Jiayun Liu, Mogen Zhang, Chen Xu, Dong Zhang, Weiyuan Ma

**Affiliations:** Department of Dermatology, Affiliated Hospital of Shandong Second Medical University, School of Clinical Medicine, Shandong Second Medical University, Weifang, Shandong, China

**Keywords:** rosacea, LL37, mast cells, TLR2/JAK2/STAT3 pathway, inflammatory response

## Abstract

**Introduction:**

Rosacea is a chronic inflammatory facial dermatosis with incompletely elucidated pathogenesis. LL37 is a key molecular mediator in rosacea development, and mast cells represent pivotal immune players in this process. However, the precise mechanism underlying LL37-induced mast cell degranulation remains undefined.

**Methods:**

A LL37-induced rosacea-like dermatitis mouse model was established with or without ruxolitinib treatment. Hematoxylin-eosin and toluidine blue staining were used to evaluate the pathological changes. Mice skin lesions were collected for transcriptome sequencing, Western blot and immunofluorescence. In vitro, the interaction between LL37 and TLR2 on the mast cell membrane surface was detected by co-immunoprecipitation and fluorescence staining. The activation of the TLR2/MyD88/JAK2/STAT3 signaling pathway was investigated using lentivirus-mediated TLR2 knockdown, MyD88 overexpression, combined with JAK2 inhibitor (ruxolitinib). Ten patients underwent VISIA skin analysis system and severity assessment following topical ruxolitinib treatment.

**Results:**

LL37 induces activation of the TLR2/JAK2 pathway in mast cells of rosacea-like mice. Ruxolitinib ameliorated cutaneous erythema and reduced mast cell infiltration and degranulation. In vitro experiments demonstrated that LL37 directly binds to TLR2, triggering TLR2/MyD88 pathway activation and subsequent mast cell degranulation. Mechanistically, MyD88 directly interacts with JAK2 to modulate the JAK2/STAT3 signaling axis, which governs mast cell degranulation. Topical application of ruxolitinib exhibited clinical efficacy in rosacea patients.

**Conclusion:**

These findings collectively demonstrate that LL37 drives mast cell activation and degranulation in rosacea pathogenesis via TLR2/JAK2/STAT3 pathway activation, while ruxolitinib effectively suppresses this signaling axis. This study provides novel mechanistic insights and therapeutic strategies for rosacea management.

## 1 Introduction

Rosacea is a chronic and multifactorial inflammatory skin disorder characterized by episodic flushing, persistent erythema, inflammatory papules, pustules, and in some cases, phymatous changes (1). These symptoms are often accompanied by debilitating subjective sensations such as burning, stinging, and itching, collectively leading to a profound psychosocial burden (2). Globally, rosacea affects approximately 0.09% to 22.41% of the population, with its prevalence varying by region and ethnicity (3). Beyond its physical manifestations, the condition significantly impacts mental health, with patients facing a markedly increased risk of depression and anxiety (2.44 and 2.18 times higher, respectively) (4). Despite its widespread prevalence and substantial clinical burden, the pathogenesis of rosacea remains incompletely elucidated, thereby hindering the development of targeted and effective therapies.

Emerging evidence suggests that innate immune dysregulation is a central driver of rosacea pathophysiology (5). Environmental and intrinsic triggers, including UV radiation, temperature fluctuations, emotional stress, and *Demodex folliculorum* colonization, initiate a cascade of immune activation. At the forefront of this process is the overactivation of Toll-like receptor 2 (TLR2) on keratinocytes, leading to excessive production of kallikrein 5 (KLK5) (6). KLK5 subsequently cleaves the antimicrobial peptide precursor hCAP18 to generate LL37, a cationic peptide with dual antimicrobial and pro-inflammatory properties (7). While LL37 is indispensable for host defense, its dysregulated expression in rosacea drives pathological inflammation. LL37 not only recruits immune cells through chemotactic signaling but also promotes vascular permeability and angiogenesis, culminating in hallmark features such as erythema, telangiectasia, and inflammatory lesions (8). Notably, elevated LL37 expression has been consistently observed in rosacea lesions, and intradermal injections of LL37 in mice induce rosacea-like phenotypes, strongly implicating LL37 as a central mediator of disease pathogenesis (9, 10).

Mast cells, essential effectors of the innate immune system, play a pivotal role in the inflammatory milieu of rosacea. These multifunctional immune cells are abundant in the dermis and respond rapidly to diverse stimuli, releasing an array of pro-inflammatory mediators, including histamine, tryptase, cytokines, and chemokines (11–13). Studies have revealed a significant increase in mast cell density within rosacea lesions, with their numbers correlating positively with disease duration and severity (14). Importantly, mast cells are indispensable for the inflammatory effects of LL37, as mast cell-deficient mice fail to develop rosacea-like dermatitis following LL37 administration (15). This underscores the critical role of mast cell activation and degranulation in the disease process.

The molecular mechanisms driving mast cell activation in rosacea remain poorly understood, though recent studies highlight the involvement of two key signaling pathways: the TLR2/MyD88 pathway and the Janus kinase/signal transducer and activator of transcription (JAK/STAT) pathway. TLR2 is a well-known immune receptor of mast cells, and its downstream adaptor protein, MyD88, is significantly upregulated in rosacea lesions (16–18). Meanwhile, the JAK/STAT signaling axis is crucial for mast cell survival, activation, and degranulation, with JAK2 serving as a key regulator (19). However, whether LL37 activates mast cells via these pathways, and how this contributes to the inflammatory cascade of rosacea, remains an open question.

This study seeks to unravel the molecular basis of LL37-induced mast cell activation and its contribution to rosacea pathogenesis. Using a combination of *in vivo* and *in vitro* approaches, we demonstrate that LL37 directly binds to TLR2, activating the TLR2/JAK2/STAT3 signaling axis in mast cells. This activation drives mast cell degranulation and the release of pro-inflammatory mediators, including β-hexosaminidase, histamine, IL-6, TNF-α, CCL2, CXCL10 and MMP-9, which collectively amplify the inflammatory response. Furthermore, we show that pharmacological inhibition of JAK2 using ruxolitinib effectively attenuates LL37-induced mast cell activation, offering a potential therapeutic strategy for rosacea. By elucidating the intricate interplay between LL37, TLR2, and JAK2/STAT3 signaling in mast cells, our findings provide novel mechanistic insights into rosacea pathogenesis and lay the groundwork for the development of targeted therapies.

## 2 Materials and methods

### 2.1 LL37-induced rosacea-like mouse model

LL37 peptide (LLGDFFRKSKEKIGKEFKRIVQRIKDFLRNLVPRTES) was synthesized by Sangon Biotech with a verified purity exceeding 95% by high-performance liquid chromatography (HPLC). Six-week-old female BALB/c mice were obtained from Beijing Vital River Laboratory Animal Technology Co., Ltd. and randomly divided into two groups (n = 5 per group). The control group received intradermal injections of phosphate-buffered saline (PBS), while the model group was injected with LL37. All animal procedures were conducted in compliance with institutional guidelines and approved by the Animal Ethics Committee of Shandong Second Medical University (Permit No. 2024SDL693).

LL37 was dissolved in sterile PBS at a concentration of 320 μM, and 40 μL of the solution was administered intradermally into the dorsal skin of each mouse every 12 hours for a total of four injections. Skin images were captured 12 hours after each injection, and erythema severity was evaluated using a scoring system: 0 (no erythema), 1 (slightly visible erythema), 2 (light erythema), 3 (obvious, clear erythema), and 4 (deep erythema with well-defined borders). The erythema area was measured using ImageJ software. Mice were euthanized 24 hours after the final injection. Anesthesia was initiated with intraperitoneal administration of ketamine (100 µg/g) and xylazine (10 µg/g). Mice were sacrificed under anesthesia by cervical dislocation and skin tissues were harvested for subsequent analyses.

### 2.2 RNA sequencing

Skin lesions from both control and model groups were subjected to transcriptomic analysis to identify key signaling pathways involved in rosacea-like dermatitis. RNA extraction, library preparation, sequencing, quality control, and bioinformatics analyses were conducted by Hangzhou Lianchuan Biotechnology Co., Ltd. Paired-end sequencing (PE150) was performed using the Illumina Novaseq™ 6000 platform. Gene expression was quantified using StringTie, with transcript levels represented as fragments per kilobase of transcript per million mapped reads (FPKM). Differentially expressed genes (DEGs) were identified using the R package *edgeR*, with a significance threshold of a fold change >2 or <0.5 and p < 0.05. Enrichment analysis for Gene Ontology (GO) terms and Kyoto Encyclopedia of Genes and Genomes (KEGG) pathways was performed using DAVID software to elucidate the biological roles and pathways associated with the DEGs.

### 2.3 Histological analysis

Skin samples were fixed in 4% paraformaldehyde for 24 hours, dehydrated, cleared, infiltrated, and embedded in paraffin. Sections (4 μm thick) were stained with hematoxylin and eosin (HE) and toluidine blue following the manufacturers’ protocols (Sangon Biotech, Shanghai, China). Stained sections were mounted with neutral balsam and examined under an optical microscope. For each sample, three sections were analyzed, and five randomly selected dermal fields per section were used for quantitative analysis of inflammatory cell infiltration and mast cell counts.

### 2.4 Immunofluorescence

Immunofluorescence staining was performed to detect the expression of TLR2, p-JAK2, and p-STAT3 in mast cells within rosacea-like dermatitis lesions, with c-kit specifically marking mast cells. Paraffin-embedded sections were deparaffinized using xylene, rehydrated through graded ethanol solutions, and subjected to antigen retrieval with citrate buffer (pH 6.0) or EDTA buffer (pH 8.0). Following incubation with 0.1% Triton X-100 for 20 minutes, nonspecific binding was blocked with 10% goat serum. Sections were incubated overnight at 4°C with primary antibodies. The primary antibodies in this study were as follows: Mouse anti-c-Kit (1:200, Santa Cruz Biotechnology, sc-365504), Rabbit-Toll-Like Receptor 2 (1:100, ZEN-BIOSCIENCE, R23333), Rabbit anti-MYD88 (1:100, ZEN-BIOSCIENCE, 340629), Rabbit anti-p-JAK2 (phospho Y1007+Y1008, 1:5000, Abcam, ab32101), Rabbit anti-p-Stat3 (phospho Tyr705, 1:2000, Cell Signaling Technology, 9145S). Fluorescently labeled secondary antibodies (ABclonal Biotechnology Co., Ltd.) were applied for 1 hour at room temperature. Nuclei were counterstained with DAPI (SparkJade, Jinan, China). Fluorescence microscopy revealed cell membranes (red), cytoplasm (green), and nuclei (blue).

### 2.5 JAK2 inhibitor treatment

Mice were assigned to four groups: PBS, PBS + JAK2 inhibitor, LL37, and LL37 + JAK2 inhibitor. The JAK2 inhibitor ruxolitinib (Incyte Corporation, Wilmington, DE, USA) was administered via oral gavage or topical application. For topical treatment, 1.5% ruxolitinib cream was applied 30 minutes after the second, third, and fourth injections of LL37 or PBS. Oral ruxolitinib was dosed at 10 mg/kg/day for 14 days, with LL37 injections administered on the last two days. Erythema severity was evaluated via erythema scoring and area measurements.

### 2.6 Cell culture and intervention

The human mast cell line LUVA (Cat. No. CTCC-001-0351) was purchased from Zhejiang Meisen Cell Technology Co., Ltd., and cultured in StemPro-34 SFM medium supplemented with 1× Glutamax. The human embryonic kidney 293T cells (Cat. No. CL-0005) were purchased from Wuhan PunoSai Life Science Technology Co., Ltd., and cultured in DMEM medium containing 10% fetal bovine serum (Sangon Biotech, Shanghai, China). Both cell lines were maintained at 37°C in a 5% CO_2_ incubator. LUVA cells were treated with 4 μM LL37 for subsequent experiments. LL37 peptides, scrambled LL37, LL37-FITC and LL37-flag were synthesized by Sangon Biotech, with purities exceeding 95%. The inhibitors (ruxolitinib and niclosamide) were obtained from Selleck Chemicals.

### 2.7 Lentiviral infection and transient transfection

Lentiviral products for TLR2 knockdown (sh-TLR2) were purchased from Shanghai Jikai Gene Technology Co., Ltd. Stable knockdown cell lines were established following puromycin selection. TLR2, HA-MyD88 and FLAG-JAK2 overexpression plasmids, as well as siRNA targeting MyD88 were transfected using TurboFect or Lipofectamine 2000 (Thermo Fisher Scientific) according to standard protocols. Gene silencing and overexpression efficiency were confirmed via RT-qPCR and Western blotting. All the overexpression plasmids used in this study were synthesized by Jinan Boshang Biotechnology Co., Ltd. siRNA-MyD88 was synthesized by Sangon Biotech.

### 2.8 Transmission electron microscopy

Mast cell samples were fixed in 2.5% glutaraldehyde (Solarbio, Beijing, China) and processed for ultra-thin sectioning. Sections were stained with uranyl acetate and lead citrate, then examined under a transmission electron microscope (Hitachi HT-7800, Hitachi, Ltd.) to assess degranulation.

### 2.9 Co-immunoprecipitation

Co-IP was conducted to confirm the interaction between LL37 and TLR2, MyD88 and JAK2. Proteins were extracted using RIPA buffer containing protease (SparkJade, Jinan, China). Antibody-protein complexes were captured on magnetic beads (Thermo Fisher Scientific) and analyzed via Western blotting.

### 2.10 Reverse transcription and real-time PCR

Total RNA was isolated using TRIzol Reagent (Thermo Fisher Scientific) and reverse-transcribed to cDNA using MightyScript First Strand cDNA Synthesis Master Mix. RT-qPCR was performed with SGExcel FastSYBR Mixture (Sangon Biotech, Shanghai, China) on a Quant Gene 9600 Real-Time PCR System. Relative expression was calculated using the 2^^−ΔΔCt^ method, with GAPDH as the reference gene. Primer sequences are listed in **Table 1**.

**Table 1:** The primers of genes for RT-qPCR.

Target gene	Forward primers(5’-3’)	Reverse primers(5’-3’)
Mouse GAPDH	TGGATTTGGACGCATTGGTC	TTTGCACTGGTACGTGTTGAT
Mouse IL-6	GGAGACGACGGAGAGTCTATT	CCATTCCAAGAACTCCCCTCTTA
Mouse TNF-α	CAGGCGGTGCCTATGTCTC	CGATCACCCCGAAGTTCAGTAG
Mouse CCL2	TTAAAAACCTGGATCGGAACCAA	GCATTAGCTTCAGATTTACGGGT
Mouse CXCL10	TGCATCAGTGACGGTAAACCA	CACAGTTTGGAGTGTTGAGGAT
Mouse MMP-9	GGACCCGAAGCGGACATTG	CGTCGTCGAAATGGGCATCT
Human GAPDH	CTGGGCTACACTGAGCACC	AAGTGGTCGTTGAGGGCAATG
Human IL-6	ACTCACCTCTTCAGAACGAATTG	CCATCTTTGGAAGGTTCAGGTTG
Human TNF-α	GAGGCCAAGCCCTGGTATG	CGGGCCGATTGATCTCAGC
Human CCL2	CAGCCAGATGCAATCAATGCC	TGGAATCCTGAACCCACTTCT
Human CXCL10	GTGGCATTCAAGGAGTACCTC	TGATGGCCTTCGATTCTGGATT
Human MMP-9	GGGACGCAGACATCGTCATC	TCGTCATCGTCGAAATGGGC

### 2.11 Western blot

Total protein was extracted from tissues or cells using RIPA buffer, and the protein concentration was measured with a BCA kit (SparkJade, Jinan, China). Equal amounts of protein were separated on 10% SDS-PAGE gels and transferred to PVDF membranes (Millipore, Burlington). The membranes were blocked with 5% BSA at room temperature for 2 hours and incubated overnight at 4°C with primary antibodies. After washing with TBS containing 0.1% Tween-20, the membranes were incubated for 1 hour at room temperature with secondary antibodies (rabbit IgG HRP-linked and mouse IgG HRP-linked, Cell Signaling Technology). Protein bands were visualized using enhanced chemiluminescence (ECL) reagent (SparkJade, Jinan, China) and imaged with a Tanon-410 automatic gel imaging system (Shanghai Tianneng Corporation). Grayscale values were quantified using Image J software, and target protein expression was normalized to α-Tubulin. The primary antibodies in this study were as follows: Rabbit anti-TLR2 (1:1500, Abclonal, A19125), Rabbit anti-MyD88 (1:1000, Abcam, ab219413), Rabbit anti-JAK2 (1:1000, Cell Signaling Technology, 3230S), Rabbit anti-p-JAK2 (phospho Y1007+Y1008, 1:5000, Abcam, ab32101), Rabbit anti-STAT3 (1:1000, Cell Signaling Technology,12640S), Rabbit anti-p-STAT3 (phospho Tyr705, 1:2000, Cell Signaling Technology, 9145S), Mouse anti-DYKDDDDK tag (1:10000, Proteintech Group, 66008-4-Ig), Rabbit anti-HA tag (1:8000, Proteintech Group, 51064-2-AP), Rabbit anti-Alpha Tubulin (1:8000, Proteintech Group, 11224-1-AP).

### 2.12 Enzyme-linked immunosorbent assay

ELISA was conducted following the manufacturer’s instructions to measure β-Hex, histamine, TNF-α, IL-6, CCL2, CXCL10 and MMP-9 levels in mouse skin tissues and cell culture supernatants (Research Cloud Biology). Optical density (OD) values were detected using a microplate reader, and cytokine concentrations were calculated based on standard curves.

### 2.13 Molecular docking

The amino acid sequences of the Death domain of MyD88 and the FERM domain of JAK2 were retrieved from UniProt. The three-dimensional structures of these domains were predicted using AlphaFold. Potential interactions between the Death domain of MyD88 and the FERM domain of JAK2 were analyzed with HDOCK. Docking scores and confidence scores were calculated, where *Confidence_score = 1.0/[1.0 + e^ (0.02(Docking_Score + 150))]*. A confidence score > 0.7 indicated high binding probability, 0.5–0.7 suggested potential binding, and < 0.5 indicated low probability. 3D protein-protein interaction diagrams were generated using PyMOL.

### 2.14 Proximity ligation assay

The Duolink^®^*In Situ* Red Mouse/Rabbit Starter Kit (Sigma-Aldrich) and Duolink^®^*In Situ* Detection Reagent Green (Sigma-Aldrich) were used for the assay. Cells were fixed for 30 minutes and permeabilized with 0.1% Triton X-100 for 15 minutes. Blocking was performed with Duolink^®^ Blocking Solution at 37°C for 1 hour. The cells were then incubated overnight at 4°C with primary antibodies against MyD88 and JAK2, followed by incubation with Duolink^®^ PLA probes at 37°C for 1 hour. After two washes, ligase solution was added, and the cells were incubated at 37°C for 30 minutes. Subsequently, the cells were incubated with amplification polymerase solution at 37°C in the dark for 100 minutes. Finally, cells were stained with DAPI-containing mounting solution for nuclear staining and coverslipped. Fluorescent images were captured using a fluorescence microscope.

### 2.15 Patient samples

Ten patients diagnosed with rosacea, based on the National Rosacea Society Expert Committee (NRSEC) criteria, were enrolled. Patients provided informed consent and were treated with topical ruxolitinib cream twice daily for 4 weeks. Facial skin condition was evaluated using the CBS^®^ (Multispectral dermoscope image processing workstation, CBS-2021, Wuhan Boshi Electronics Co., Ltd) and scoring systems, including the Global Flushing Severity Scale (GFSS), Clinician’s Erythema Assessment (CEA), and Patient’s Self-Assessment (PSA) scales. The study was approved by the Ethics Review Committee of Shandong Second Medical University and conducted in accordance with the principles of the Declaration of Helsinki (Permit Number: wyfy-2024-ky-282).

### 2.16 Statistical analyses

Data are expressed as mean ± standard deviation (mean ± SD). Statistical analyses were performed using SPSS v26.0 and GraphPad Prism 9.0. Two-group comparisons were conducted using an unpaired Student’s t-test, and comparisons among multiple groups were performed with one-way analysis of variance (ANOVA), followed by Tukey’s *post hoc* test. Sample sizes (n) for each experiment are reported in figure legends. A P-value < 0.05 was considered statistically significant.

## 3 Results

### 3.1 LL37 induces activation of the TLR2/JAK2 pathway in mast cells of rosacea-like mice

**Figure 1:**
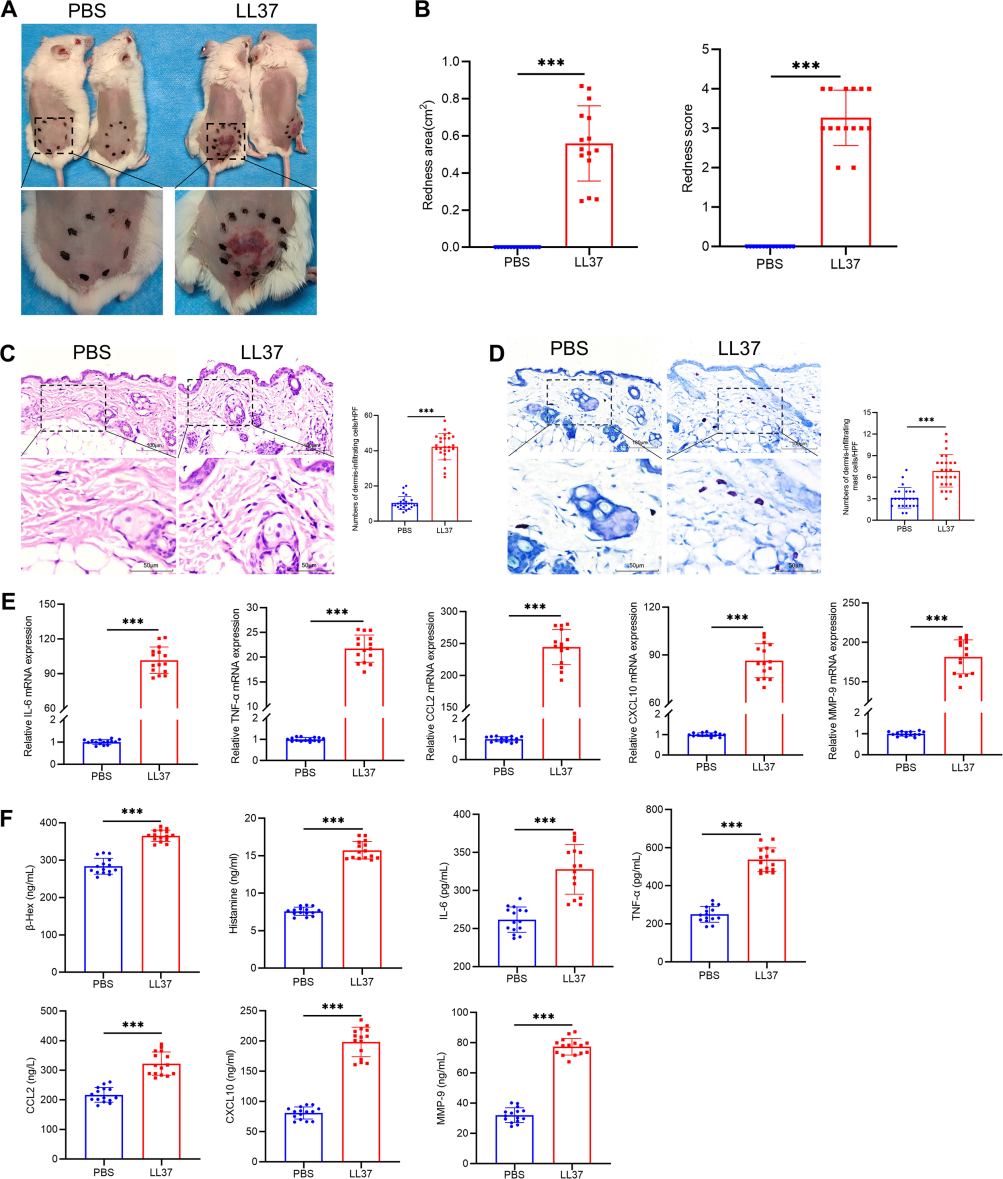
Establishment of LL37-induced rosacea-like dermatitis mouse model. **(A)** Representative images of rosacea-like lesions in each group 12h after the final LL37 injection. **(B)** Severity of rosacea-like lesions evaluated based on redness area and redness score. Representative H&E **(C)** and toluidine blue **(D)** staining of skin/lesion tissue sections. Quantitative analysis of dermal infiltrating cells and mast cells per high-power field. **(E)** mRNA levels of IL-6, TNF-α, CCL2, CXCL10 and MMP-9 in skin/lesion tissues. **(F)** Secretion levels of β-Hex, histamine, IL-6, TNF-α, CCL2, CXCL10 and MMP-9 in skin/lesion tissues. n=5 per group; data are presented as mean ± SD of at least three independent experiments, ***P < 0.001. Scale bar: 50 μm.

To explore the mechanisms underlying rosacea pathogenesis, we established a mouse model of rosacea-like dermatitis through intradermal LL37 injection. Within 12 hours of the final injection, the mice exhibited erythema and capillary dilation at the injection site, accompanied by visible dermal abnormalities (**Figures 1A, B**). Hematoxylin-eosin staining revealed substantial dermal thickening and inflammatory cell infiltration in LL37-treated mice compared to the control group (**Figure 1C**). Mast cells, identified by toluidine blue staining, were markedly increased in the dermis of rosacea-like lesions (**Figure 1D**). To further investigate molecular changes, we analyzed the expression of mast cell degranulation-related markers (β-Hex, histamine, IL-6, TNF-α, CCL2, CXCL10 and MMP-9) using RT-qPCR and ELISA. The results revealed significantly elevated mRNA and protein levels in the LL37-treated group compared to controls (**Figures 1E, F**).

**Figure 2:**
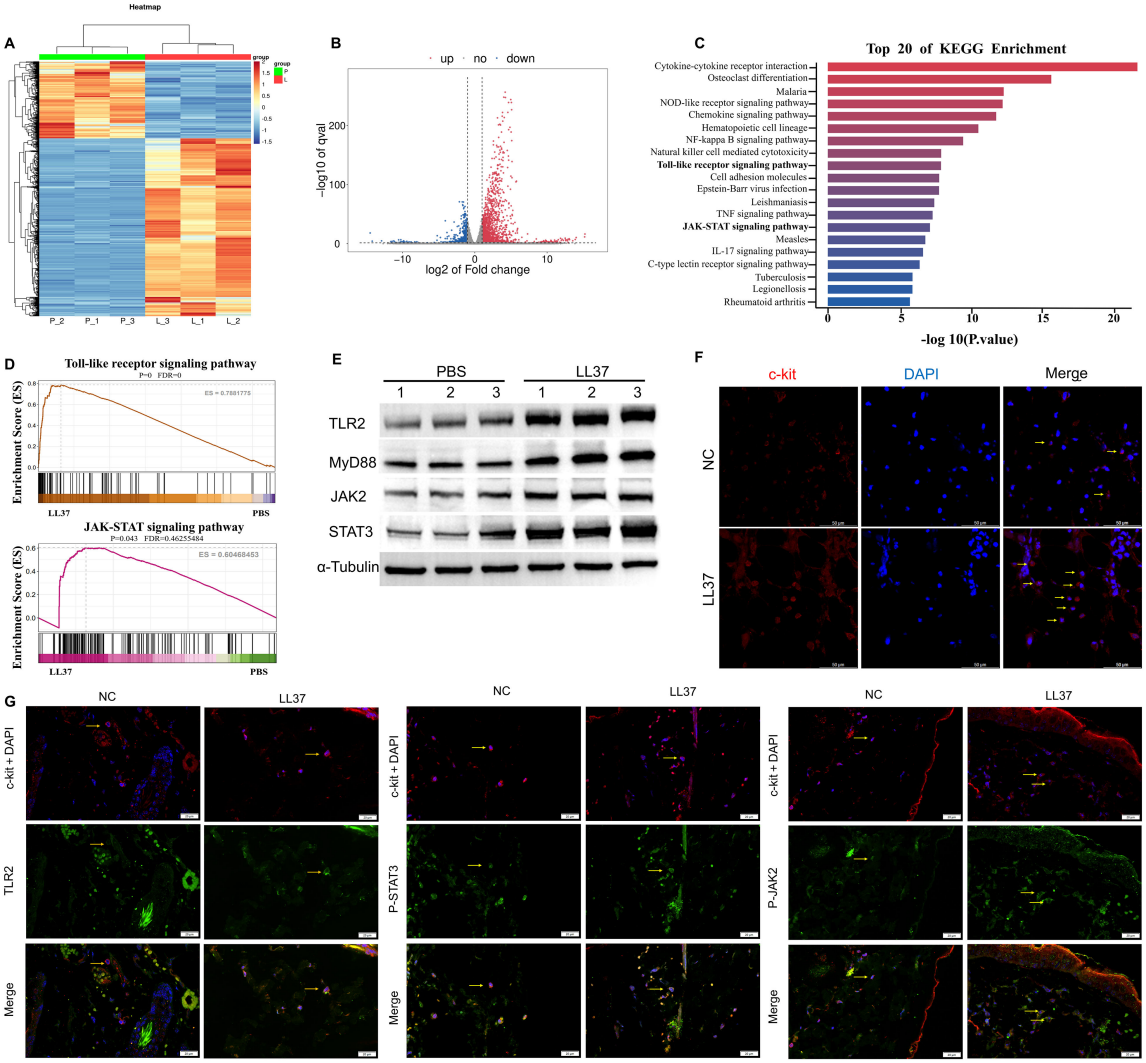
Activation of the TLR2/JAK2 pathway in mast cells of LL37-induced rosacea-like mice. **(A)** Heatmap of differentially regulated transcripts in skin/lesion tissues between control (P: normal skin) and model (L: lesional skin) groups. Blue indicates low FPKM expression; red indicates high FPKM expression. Volcano plot of differentially expressed genes **(B)** and KEGG pathway enrichment analysis **(C)** in skin/lesion tissues. **(D)** GSEA analysis showing enrichment of Toll-like receptor and JAK-STAT signaling pathways. **(E)** Expression levels of TLR2, MyD88, JAK2 and STAT3 in skin/lesion tissues. The blots shown are representative of three independent experiments. **(F)** Immunofluorescence staining of c-kit (red) and DAPI (blue) in skin/lesion tissues. **(G)** Co-localization of c-kit (red) with TLR2, p-JAK2 and p-STAT3 (green) in skin/lesion tissues. Scale bar: 50 μm.

We subsequently performed transcriptome sequencing on lesional skin from LL37-induced rosacea-like dermatitis mice and skin from control mice. Hierarchical clustering analysis revealed marked differences in gene expression patterns between rosacea-like dermatitis lesions and normal skin (**Figure 2A**). Transcriptome sequencing of skin lesions identified 2166 differentially expressed genes (DEGs) between LL37-treated and control mice, including 1511 upregulated and 655 downregulated genes (*P* < 0.05, |log2(fold change)| > 2, **Figure 2B**). Gene set enrichment analysis (GSEA) highlighted significant activation of pathways, including Toll-like receptor and JAK/STAT pathway, which were significantly enriched (*P* < 0.05, **Figures 2C, D**). Western blot analysis further confirmed upregulation of TLR2, MyD88, JAK2 and STAT3 in skin lesions of LL37-treated mice compared to controls (**Figure 2E**). Immunofluorescence revealed that the number of c-kit-labeled mast cells in the model group was significantly higher than that in the control group (**Figure 2F**). Additionally, the fluorescence intensities of TLR2, p-JAK2, and p-STAT3 in mast cells within the skin lesions of the model group were markedly elevated compared to the control group (**Figure 2G**). These findings collectively indicate that the TLR2/JAK2/STAT3 signaling pathway plays a pivotal role in mast cell activation and degranulation in LL37-induced rosacea-like dermatitis.

### 3.2 JAK2 inhibitor significantly alleviates rosacea-like lesions in mice

**Figure 3:**
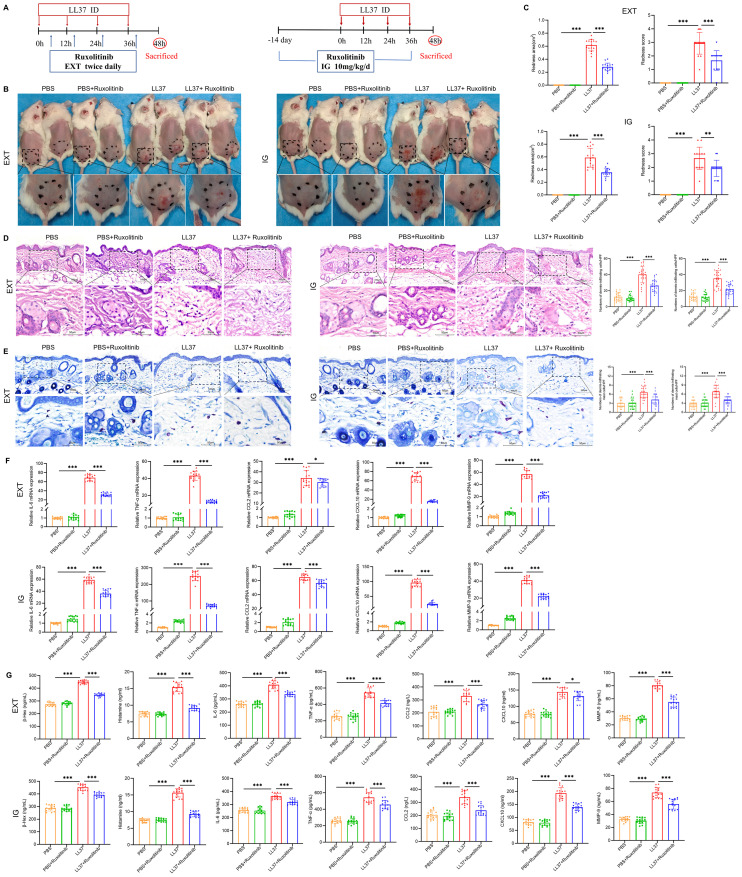
JAK2 inhibitor alleviates rosacea-like lesions in mice. **(A)** Schematic of LL37-induced model and ruxolitinib treatment. EXT: External Application, IG: Intragastric Administration. **(B)** Representative images of lesions post- ruxolitinib treatment. **(C)** Lesion severity assessed by redness area and score. H&E **(D)** and toluidine blue **(E)** staining of skin/lesion tissues. **(F)** mRNA levels of IL-6, TNF-α, CCL2, CXCL10 and MMP-9. **(G)** Secretion levels of β-Hex, histamine, and cytokines. n=5 per group; data are presented as mean ± SD of at least three independent experiments, *P < 0.05, **P < 0.01, ***P < 0.001. Scale bar: 50 μm.

To evaluate the therapeutic potential of targeting JAK2 signaling in rosacea, LL37-induced mice were treated with ruxolitinib (a JAK1/2 inhibitor) via intragastric administration (IG) or external application (EXT) (**Figure 3A**). Both treatment modalities resulted in significant reductions in redness score and redness area compared to untreated controls (**Figures 3B, C**). Histological analysis showed markedly reduced dermal inflammation, mast cell infiltration, and vascular abnormalities in ruxolitinib-treated mice (**Figures 3D, E**). In addition, RT-qPCR and ELISA demonstrated a significant decrease in β-Hex, histamine, IL-6, TNF-α, CCL2, CXCL10 and MMP-9 expression in ruxolitinib-treated groups compared to untreated LL37-induced mice (**Figures 3F, G**). These findings suggest that inhibiting JAK2 signaling effectively mitigates rosacea-like skin inflammation in the LL37-induced model.

### 3.3 LL37 directly induces mast cell activation and degranulation

**Figure 4:**
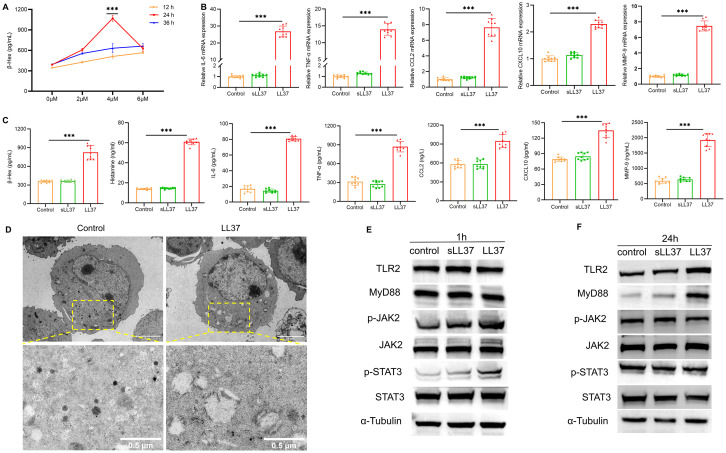
LL37 induces mast cell activation and degranulation. **(A)** β-Hex secretion in LUVA cells treated with LL37 at varying concentrations/durations. **(B)** mRNA levels of IL-6, TNF-α, CCL2, CXCL10 and MMP-9 in cells of each group. **(C)** Secretion levels of β-Hex, histamine, IL-6, TNF-α, CCL2, CXCL10 and MMP-9 in cells of each group. **(D)** Transmission electron microscopy images of degranulation in LL37-treated mast cells. **(E)** Expression of TLR2, MyD88, p-JAK2 and p-STAT3 in cells of each group after 1h incubation with LL37. **(F)** Expression of TLR2, MyD88, p-JAK2 and p-STAT3 in cells from each group after 24h incubation with LL37. *n* = 3 per group; data are presented as mean ± SD of at least three independent experiments, ***P < 0.001.

To investigate whether LL37 directly induces mast cell activation, the release of β-Hex from LUVA cells was measured following treatment with different concentrations of LL37 at various time points. The results showed that at a concentration of 4 µM LL37 incubated for 24 hours, the release of β-Hex was significantly increased (**Figure 4A**). Therefore, 4 µM LL37 was selected for subsequent mast cell experiments. Subsequent experiments revealed that LL37 treatment significantly increased β-Hex and histamine release, as well as the mRNA and protein levels of IL-6, TNF-α and other cytokines (**Figures 4B, C**). However, this result was not observed in the scrambled LL37 treatment group, indicating that LL37 can directly induce mast cell activation and degranulation.

Transmission electron microscopy (TEM) of LL37-treated mast cells showed a marked reduction in electron-dense granules (**Figure 4D**). Granule-depleted areas of the cytoplasm displayed vacuole-like structures where granular material had been released. Furthermore, to further investigate whether the TLR2/JAK2/STAT3 signaling pathway is involved in LL37-induced mast cell activation and degranulation, western blot analysis was performed to detect the protein expression of key signaling molecules in LUVA cells following stimulation with LL37 or scrambled LL37. The results showed that after 1 hour of LL37 incubation, the phosphorylation levels of JAK2 and STAT3 were significantly elevated compared to the control group (**Figure 4E**). After 24 hours of incubation, the expression levels of TLR2 and MyD88 were markedly higher than in the control group, whereas no such changes were observed in the scrambled LL37 treatment group (**Figure 4F**). Collectively, these findings suggest that LL37 directly induces mast cell activation and degranulation, a process potentially linked to the activation of the TLR2/MyD88 and JAK2/STAT3 signaling pathways.

### 3.4 LL37 activates mast cells via direct interaction with TLR2

**Figure 5:**
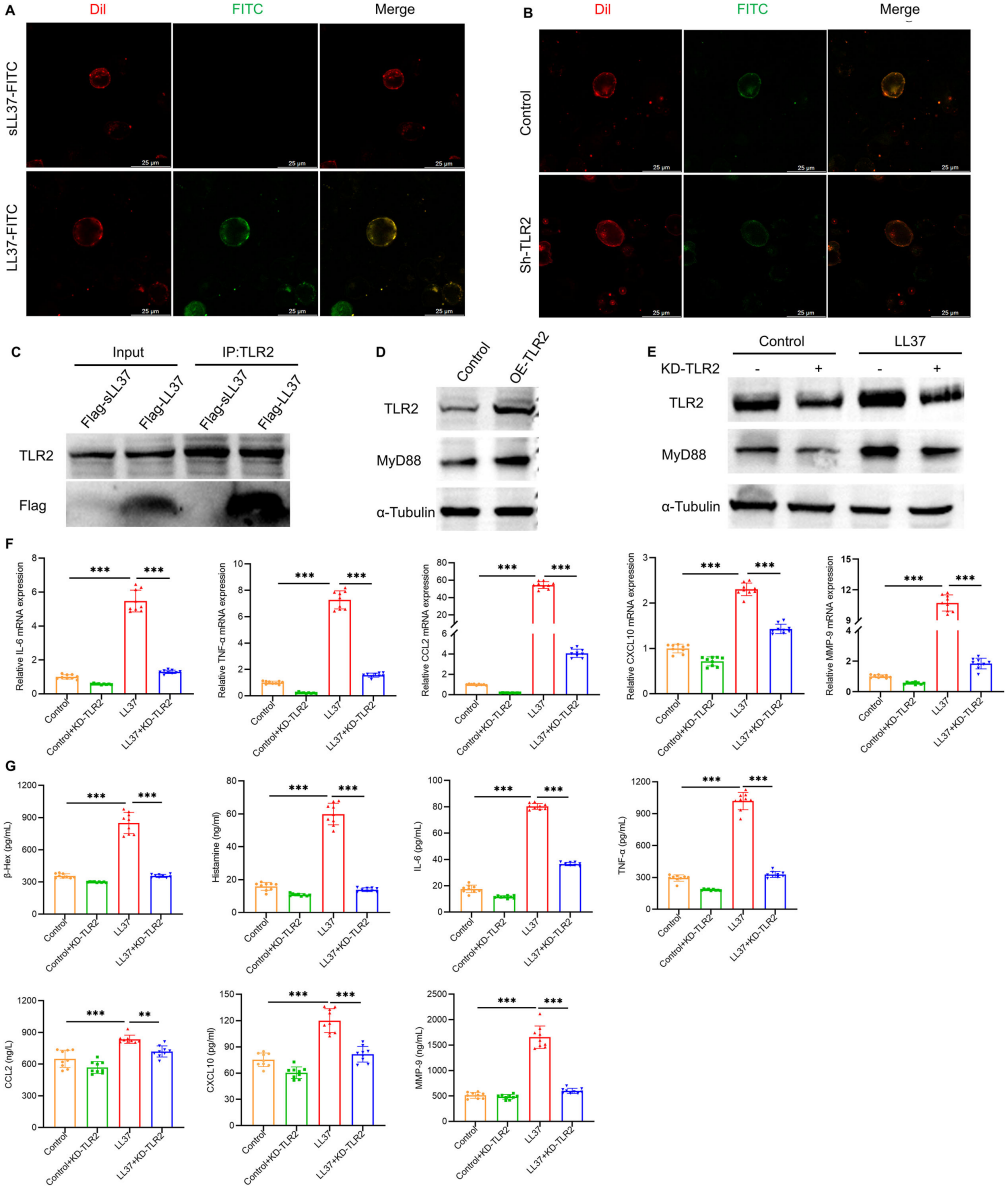
LL37 binds to TLR2 on mast cells to induce activation and degranulation. **(A)** Membrane localization of LL37/sLL37 in LUVA cells. Scale bar: 25 μm. **(B)** Membrane localization of LL37 in TLR2-knockdown mast cells. **(C)** Co-immunoprecipitation of LL37-flag with TLR2. **(D)** Expression levels of TLR2 and MyD88 in TLR2-overexpressing. **(E)** Expression levels of TLR2 and MyD88 in cells of each group. **(F)** mRNA levels of IL-6, TNF-α, CCL2, CXCL10 and MMP-9 in cells of each group. **(G)** Secretion levels of β-Hex, histamine, IL-6, TNF-α, CCL2, CXCL10 and MMP-9 in cells of each group. *n* = 3 per group; data are presented as mean ± SD of at least three independent experiments, **P < 0.01, ***P < 0.001.

To further investigate the functional relationship between LL37 and the TLR signaling pathway in LUVA cells, mast cells were co-incubated with FITC-conjugated LL37 or scrambled LL37, along with the membrane-binding red fluorescent probe Dil. Scrambled LL37, a randomized-sequence control peptide lacking the native structure and bioactivity, was used to exclude non-specific effects from the peptide’s inherent charge or properties. Microscopic analysis revealed that LL37 colocalized with Dil on the mast cell membrane, whereas scrambled LL37 failed to localize to the membrane (**Figure 5A**). Following TLR2 knockdown mediated by lentiviral shRNA, fluorescence staining using FITC-labeled LL37 demonstrated colocalization of LL37 with the plasma membrane of mast cells, a process significantly reduced by TLR2 knockdown (**Figure 5B**). Co-immunoprecipitation experiments further confirmed the direct interaction between LL37 and TLR2 (**Figure 5C**). These results demonstrate that LL37 binds to TLR2 on the mast cell membrane surface.

In mast cells with TLR2 overexpression, there is a marked upregulation in the expression of MyD88, a canonical downstream signaling molecule of the TLR2 pathway (**Figure 5D**). Knockdown of TLR2 using shRNA significantly inhibited LL37-induced MyD88 expression and downstream signaling (**Figure 5E**). Meanwhile, stimulation of TLR2-knockdown mast cells with LL37 failed to induce increased mRNA expression levels of degranulation-related cytokines including IL-6, TNF-α, CCL2, CXCL10, and MMP-9 (**Figure 5F**). Furthermore, the release levels of these cytokines along with β-Hex and histamine in the cell culture supernatant were also significantly suppressed (**Figure 5G**). The above results indicate that LL37 can directly interact with TLR2 on the surface of mast cell membrane, thereby triggering the activation of the TLR2/MyD88 signaling pathway, which induces mast cell activation and degranulation.

### 3.5 TLR2/MyD88 signaling regulates JAK2/STAT3 activation in mast cells

**Figure 6:**
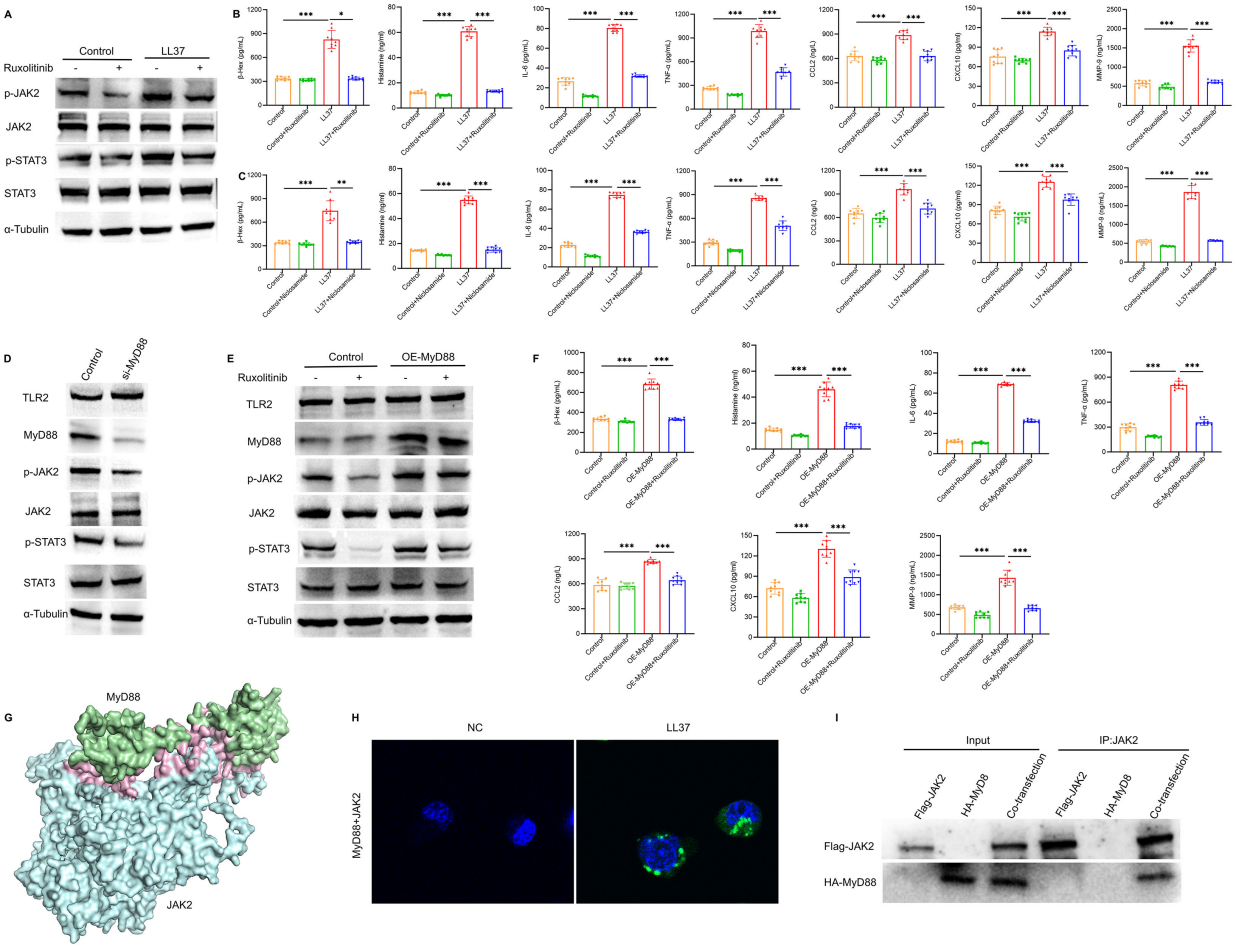
TLR2/MyD88 signaling regulates JAK2/STAT3 activation in mast cells. **(A)** Expression levels of p-JAK2 and p-STAT3 in LUVA cells of each group. **(B, C)** Secretion levels of β-Hex, histamine, IL-6, TNF-α, CCL2, CXCL10 and MMP-9 in cells of each group. **(D)** Expression of TLR2, MyD88, p-JAK2 and p-STAT3 in MyD88-knockdown cells. **(E)** Expression levels of TLR2, MyD88, p-JAK2 and p-STAT3 in cells of each group. **(F)** Secretion levels of β-Hex, histamine, IL-6, TNF-α, CCL2, CXCL10 and MMP-9 in cells of each group. **(G)** Three-dimensional model of MyD88-JAK2 molecular docking. **(H)** PLA showing MyD88-JAK2 the physical interaction (green dots). **(I)** Co-IP demonstrates the interaction between MyD88 and JAK2. *n* = 3 per group, data are presented as mean ± SD of at least three independent experiments, *P < 0.05, **P < 0.01, ***P < 0.001.

Further analysis revealed that LL37 stimulation significantly increased JAK2 and STAT3 phosphorylation in mast cells, which was accompanied by enhanced β-Hex release and cytokine production. Treatment with ruxolitinib suppressed these effects (**Figures 6A, B**). To further elucidate the role of STAT3 in mast cell activation and degranulation, niclosamide was used to inhibit the phosphorylation of STAT3. ELISA results demonstrated that niclosamide significantly inhibited LL37-induced release of β-Hex and degranulation-related cytokines in mast cells (**Figure 6C**). These findings indicate that LL37-induced mast cell degranulation and cytokine release are mediated through the phosphorylation of the JAK2/STAT3 signaling pathway.

To explore the relationship between TLR2/MyD88 and JAK2/STAT3 signaling, we overexpressed MyD88 and inhibited JAK2 phosphorylation using ruxolitinib for further analysis. MyD88 overexpression rescued the decline in JAK2 phosphorylation, while MyD88 knockdown significantly reduced JAK2 and STAT3 phosphorylation (**Figures 6D, E**). Similarly, ruxolitinib treatment attenuated the increased release of mast cell degranulation markers, including β-Hex and histamine, as well as related cytokines, induced by MyD88 overexpression (**Figure 6F**).

Based on these findings, we performed molecular docking between the Death domain of MyD88 protein and the FERM domain of JAK2 protein. The results revealed an optimal binding model with a docking score of -239.48 and a confidence score of 0.8569, indicating a high likelihood of interaction between MyD88 and JAK2 (**Figure 6G**). Proximity ligation assay (PLA) results showed that MyD88 physically interacted with JAK2 in mast cells following LL37 treatment (**Figure 6H**). Additionally, co-immunoprecipitation (Co-IP) experiments confirmed the interaction between MyD88 and JAK2 (**Figure 6I**). These findings suggest that TLR2/MyD88 directly activates the JAK2/STAT3 signaling pathway, leading to mast cell activation and degranulation.

### 3.6 Topical ruxolitinib treatment shows clinical efficacy in rosacea patients

**Figure 7:**
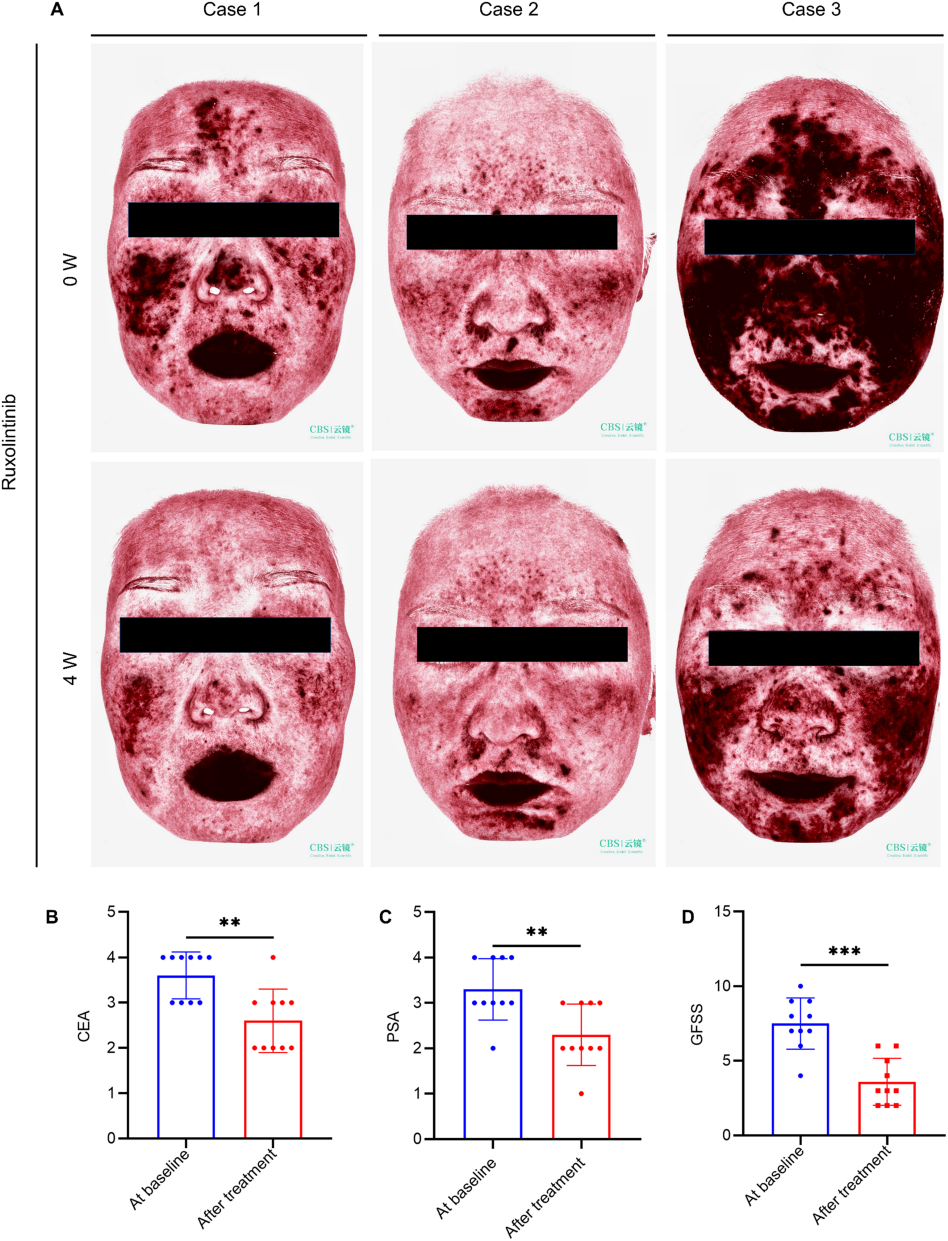
Topical ruxolitinib improves rosacea lesions in patients. **(A)** VISIA imaging of facial lesions in 3 erythematotelangiectatic rosacea (ETR) patients pre- and post-treatment. Changes in Clinician’s Erythema Assessment **(CEA) (B)**, Patient Self-Assessment (PSA) **(C)** and Global Flushing Symptom Score (GFSS) **(D)**. Data are presented as mean ± SD of at least three independent experiments, **P < 0.01, ***P < 0.001.

To assess clinical efficacy, topical ruxolitinib was applied to 10 rosacea patients for four weeks. The treatment significantly improved erythema and papules, as reflected by reductions in CEA, PSA, and GFSS scores (**Figures 7A–D**). Most patients reported mild itching or burning sensations during the first week, but no severe adverse events were observed, indicating good safety and tolerability.

## 4 Discussion

Mast cells are central players in the immune system, contributing to both innate and adaptive immunity as well as immune regulation. Excessive mast cell activation has been strongly linked to the pathogenesis of numerous allergic and inflammatory diseases, including bronchial asthma, allergic rhinitis, urticaria, and rosacea. This activation is characterized by mast cell infiltration into inflamed tissues and the subsequent release of a broad spectrum of bioactive mediators (20). Mature mast cells express a variety of membrane receptors, such as FcϵRI, TLRs, KIT, NKRs, and Mrgprs, among others (21). Each receptor plays a distinct role in mast cell activation, leading to downstream inflammatory responses. For example, allergen-specific IgE binding to FcϵRI triggers mast cell degranulation, releasing histamine, which is central to acute allergic reactions like urticaria.

Mast cells have increasingly been recognized as key contributors to the pathogenesis of skin diseases, including rosacea (22, 23). In rosacea, mast cell degranulation is a pivotal pathological event, leading to the release of histamine, tryptase, inflammatory cytokines, chemokines, and vascular endothelial growth factors (15, 24). These mediators contribute to various clinical manifestations of rosacea, such as erythema, telangiectasia, and the papulopustular subtype. Among these, vascular endothelial growth factors (VEGF) platelet-derived growth factor (PDGF), and fibroblast growth factor-2 (FGF-2) orchestrate vascular and lymphatic remodeling, promoting persistent capillary dilation and lymphatic edema characteristic of erythematotelangiectatic rosacea. Additionally, inflammatory cytokines like TNF-α and IL-6 activate macrophages and endothelial cells, facilitating leukocyte infiltration and contributing to papulopustular rosacea. Notably, cytokines such as TNF-α, IL-6, and IL-8 stimulate nociceptors, leading to the burning and stinging sensations commonly experienced by rosacea patients (25).

In recent years, increasing evidence has highlighted the therapeutic potential of targeting mast cell activation in rosacea. For example, botulinum toxin and sodium cromoglycate have been shown to prevent mast cell degranulation, reducing inflammation and alleviating symptoms in both animal models and patients (15, 26). These findings underscore the importance of further elucidating the molecular mechanisms underlying mast cell activation in rosacea to identify novel therapeutic targets.

In this study, we demonstrated that LL37, an antimicrobial peptide implicated in the pathogenesis of rosacea, directly binds to TLR2 on mast cells, triggering their degranulation. TLR2, a highly expressed pattern recognition receptor on mast cells, plays a crucial role in innate immune responses (17). Our findings confirmed that LL37 induces mast cell activation through the TLR2/MyD88 signaling pathway, promoting the release of inflammatory mediators and chemokines that drive rosacea pathology. Importantly, we also provided evidence for cross-talk between the TLR2/MyD88 and JAK2/STAT3 pathways in mast cell activation. Specifically, we demonstrated that MyD88 overexpression rescues the inhibition of JAK2 phosphorylation by ruxolitinib, suggesting a functional interaction between these pathways.

The JAK/STAT pathway is a well-known mediator of cell proliferation, differentiation, and immune regulation and has been implicated in various inflammatory and autoimmune skin diseases (27–29). In rosacea, we found that the JAK2/STAT3 signaling pathway is significantly activated in mast cells and LL37-induced skin lesions. The activation of this pathway contributes to mast cell degranulation and the production of pro-inflammatory cytokines. Combining the results of molecular docking between MyD88 and JAK2 proteins, proximity ligation assay (PLA), and co-immunoprecipitation (Co-IP), we demonstrated that the TLR2/MyD88 pathway directly regulates the JAK2/STAT3 signaling pathway, which is involved in LL37-induced mast cell activation and degranulation.

These findings are consistent with previous studies demonstrating the role of JAK/STAT signaling in inflammatory skin diseases. For example, JAK inhibitors such as tofacitinib have shown significant efficacy in reducing inflammation in psoriasis and atopic dermatitis (30, 31). In this study, we showed that ruxolitinib, a JAK2 inhibitor, effectively alleviates erythema and inflammation in an LL37-induced mouse model of rosacea. Moreover, preliminary clinical trials indicated that topical ruxolitinib significantly reduces erythema and papules in rosacea patients, highlighting its potential as a novel therapeutic strategy.

Despite these advances, several limitations remain. Due to the non-invasive nature of rosacea diagnosis and the cosmetic concerns associated with skin biopsies, collecting patient-derived skin lesions for histopathological validation is challenging. While our findings were robustly demonstrated in animal models, additional studies are needed to confirm these results in human tissues.

**Figure 8:**
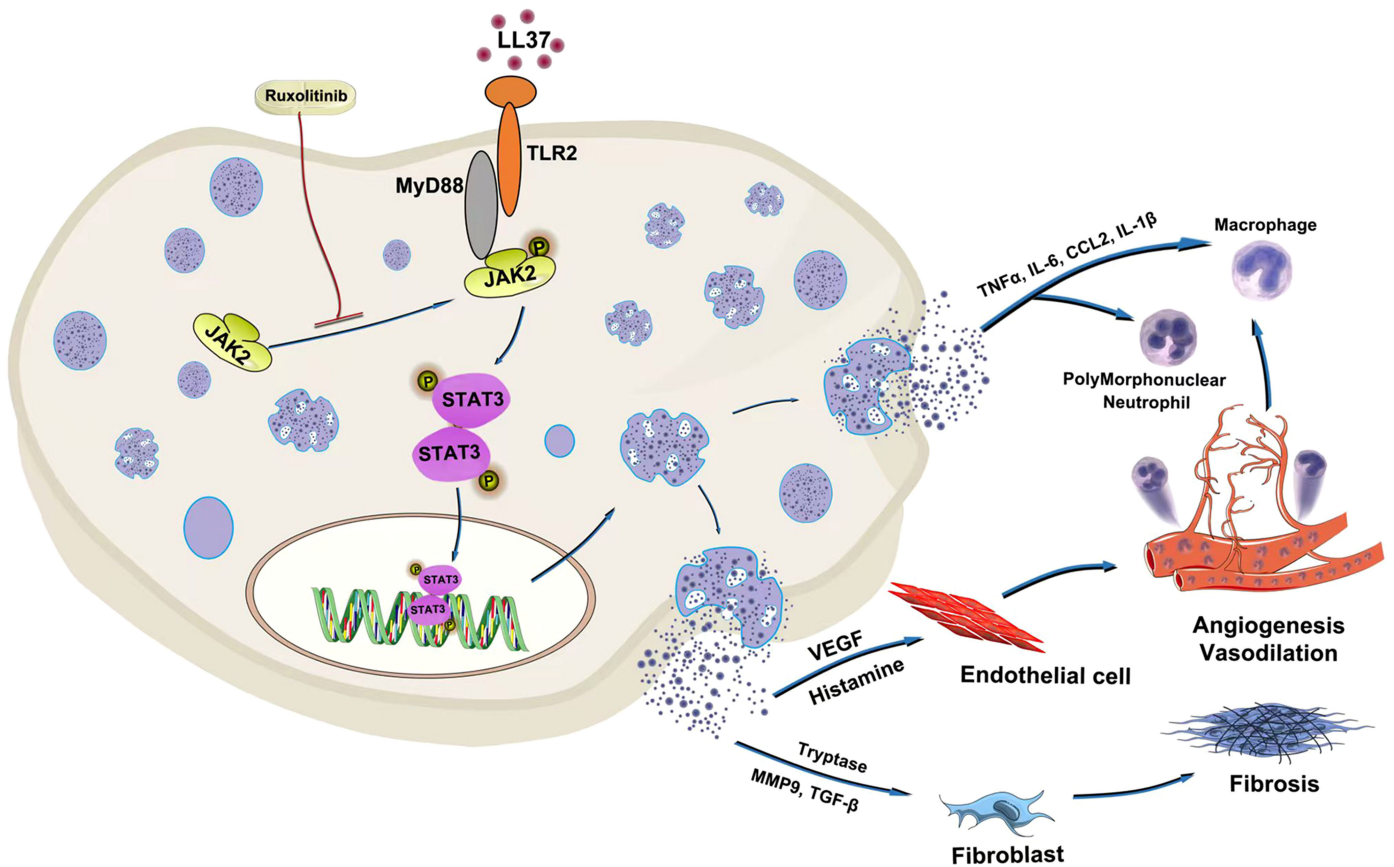
Schematic diagram of LL37-driven mast cell degranulation and inflammation in rosacea. LL37 activates mast cells via direct interaction with TLR2. TLR2/MyD88 signaling regulates JAK2/STAT3 activation in mast cells. The JAK2 inhibitor ruxolitinib demonstrates therapeutic efficacy against LL37-driven mast cell degranulation.

In summary, our study identifies the TLR2/MyD88/JAK2/STAT3 signaling pathway as a critical mediator of mast cell activation and degranulation in rosacea (**Figure 8**). These findings provide novel insights into the molecular mechanisms driving rosacea pathogenesis and support the development of targeted therapies. The therapeutic potential of JAK inhibitors, such as ruxolitinib, represents a promising direction for future research and clinical application.

## Data Availability

The raw sequence data reported in this paper have been deposited in the Genome Sequence Archive (Genomics, Proteomics & Bioinformatics 2025) in National Genomics Data Center (Nucleic Acids Res 2025), China National Center for Bioinformation / Beijing Institute of Genomics, Chinese Academy of Sciences (GSA: CRA033605) that are publicly accessible at https://ngdc.cncb.ac.cn/gsa.
