# Supplementary figures and images for: LL37-driven mast cell degranulation and inflammation in rosacea via TLR2/JAK2/STAT3 axis
Source: Front Immunol. 2025 Nov 26;16:1672021. doi: 10.3389/fimmu.2025.1672021 (PMC12689332; doi:10.3389/fimmu.2025.1672021)

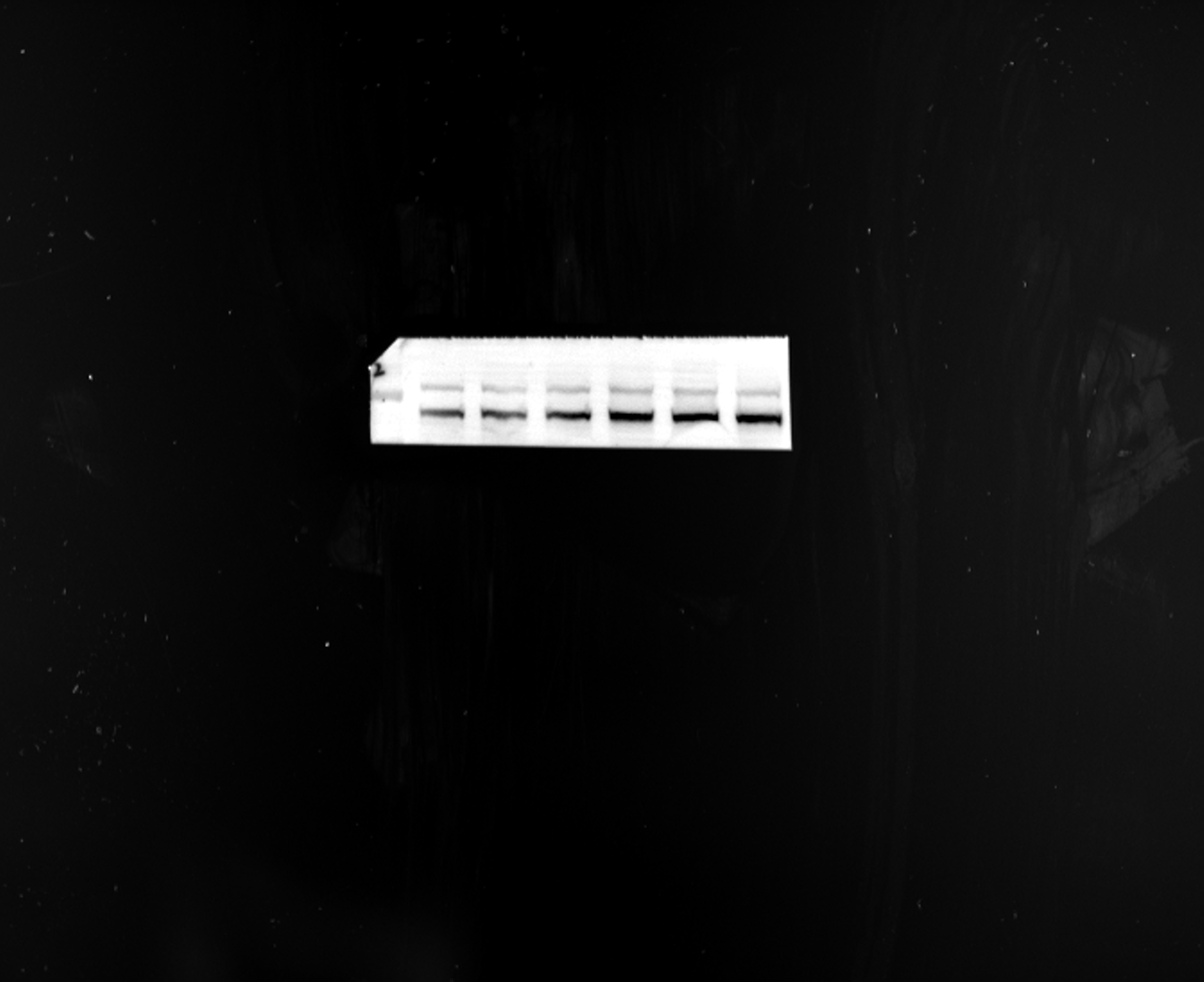

Supplement: Supplementary file 1 [file DataSheet1.zip › raw data traces-Figure 2E/JAK2/JAK2.Tif]

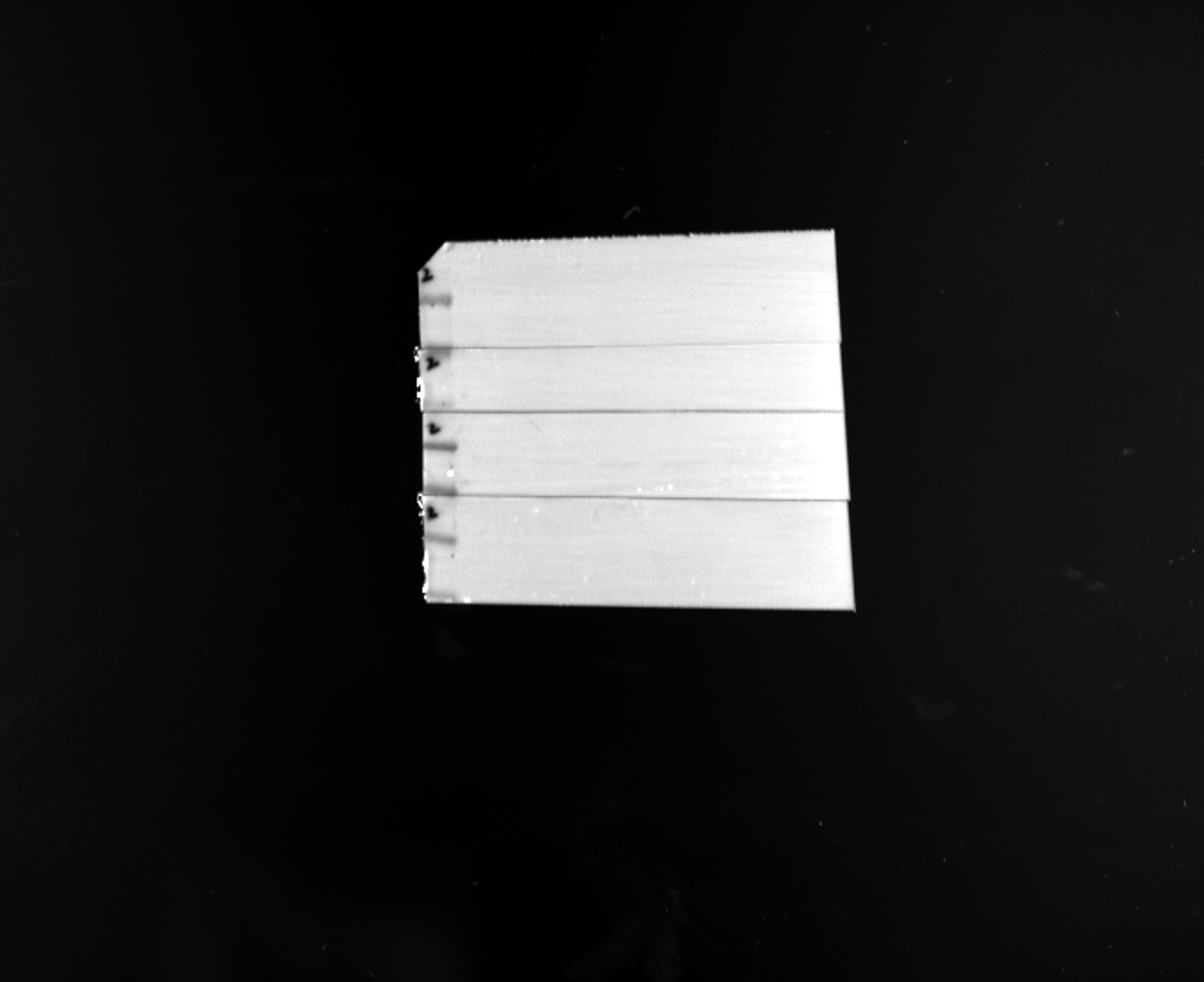

Supplement: Supplementary file 1 [file DataSheet1.zip › raw data traces-Figure 2E/JAK2/Uncropped images of blots.Tif]

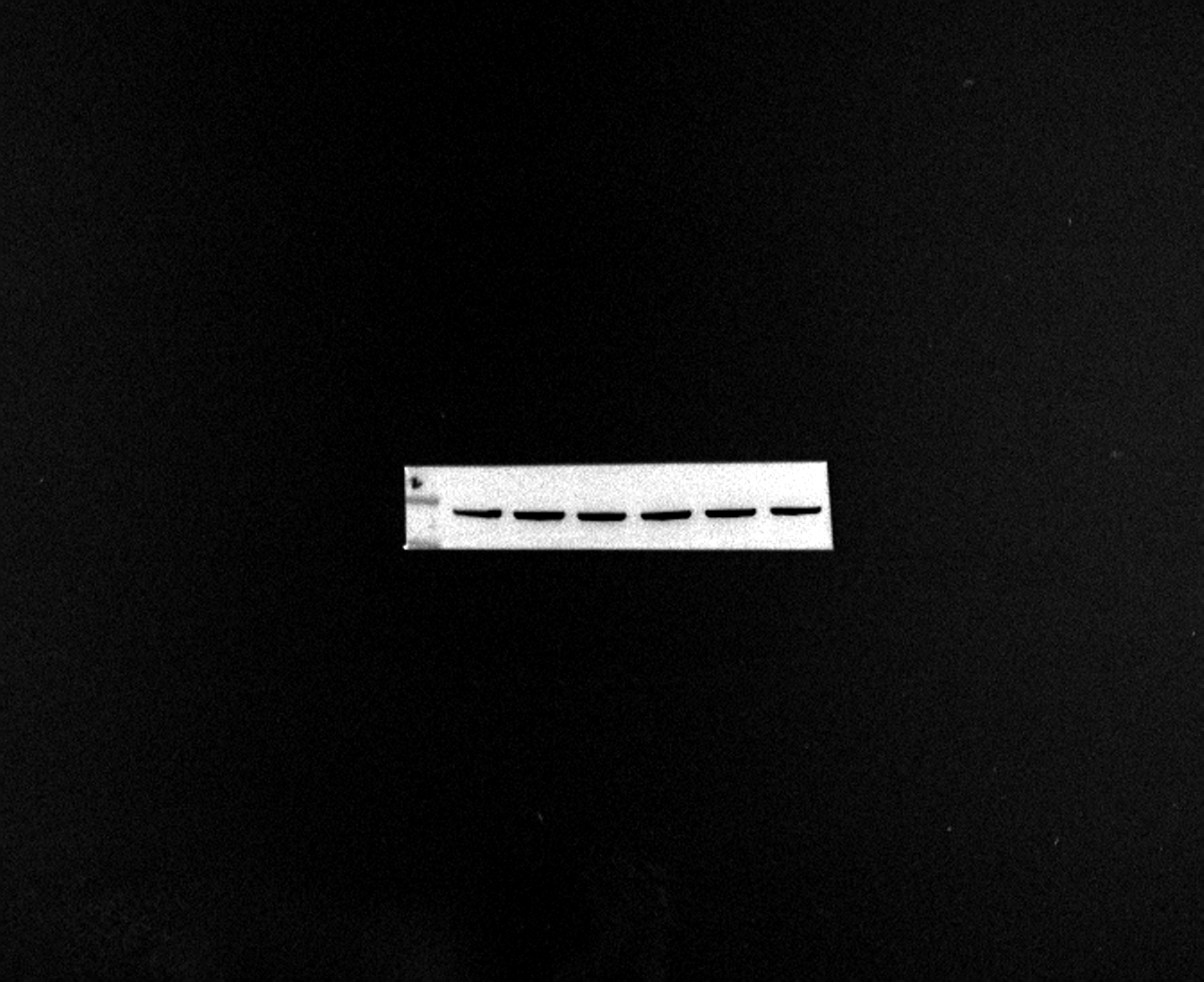

Supplement: Supplementary file 1 [file DataSheet1.zip › raw data traces-Figure 2E/JAK2/α-tubulin.Tif]

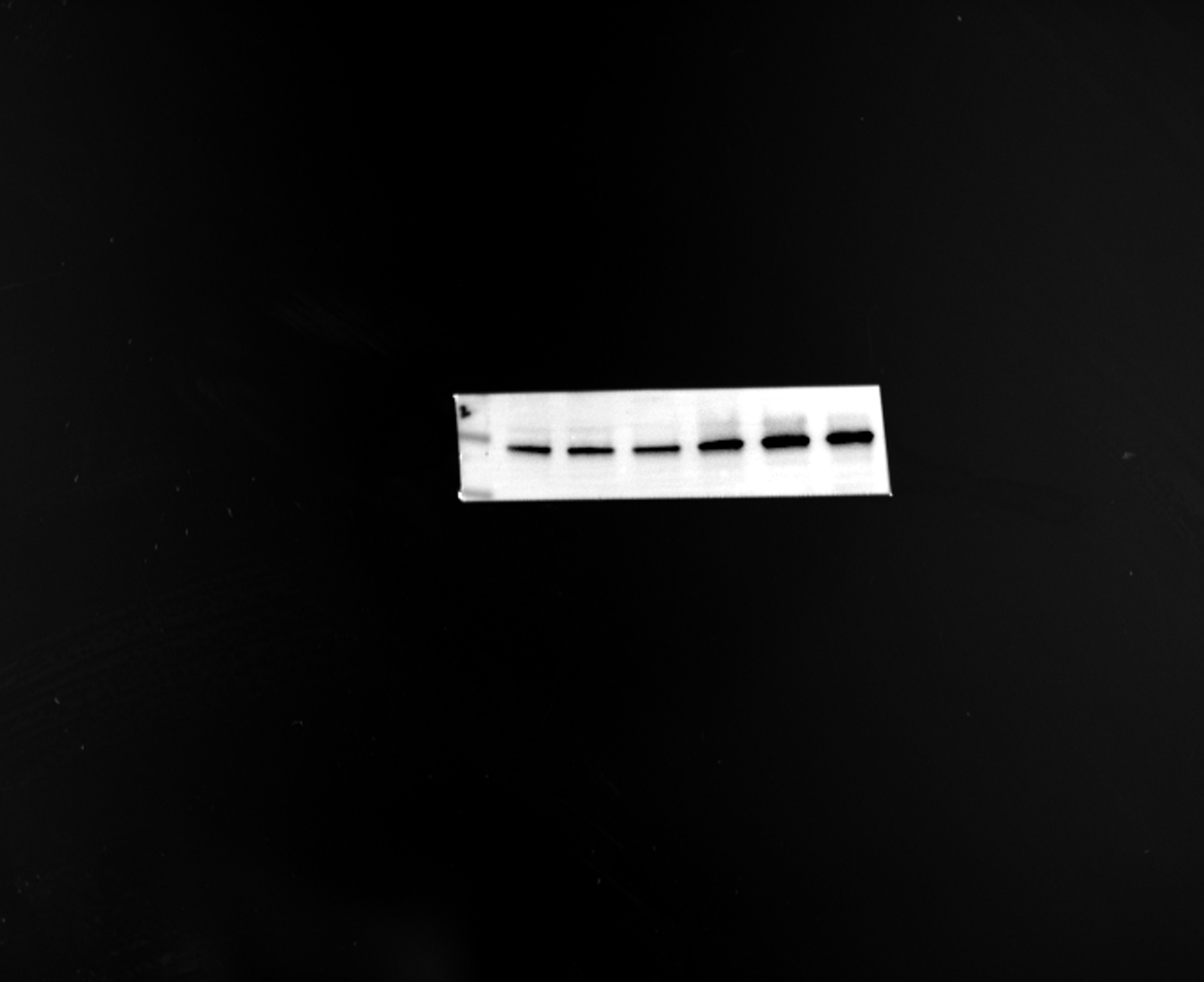

Supplement: Supplementary file 1 [file DataSheet1.zip › raw data traces-Figure 2E/MyD88/MyD88.Tif]

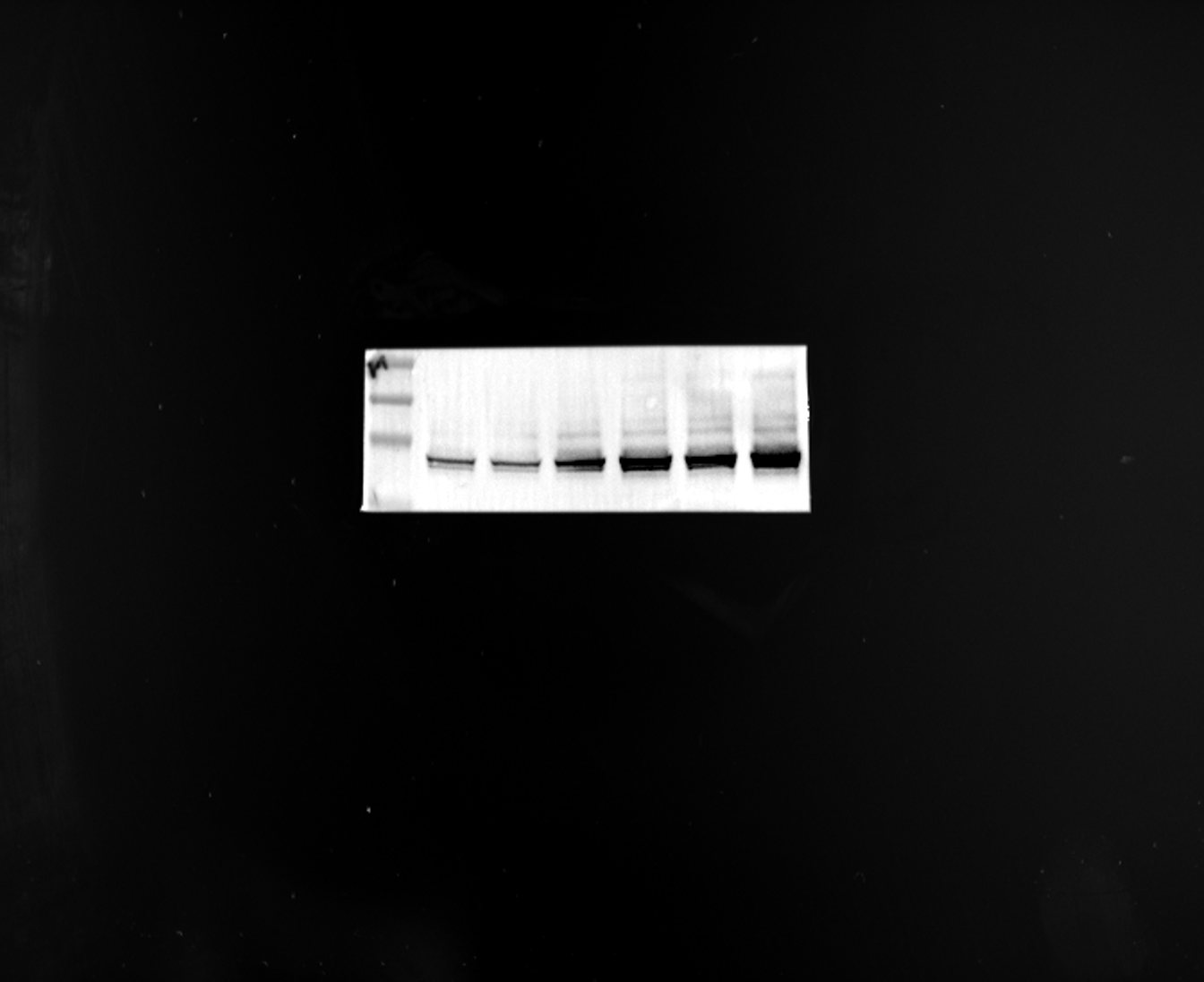

Supplement: Supplementary file 1 [file DataSheet1.zip › raw data traces-Figure 2E/STAT3/STAT3.Tif]

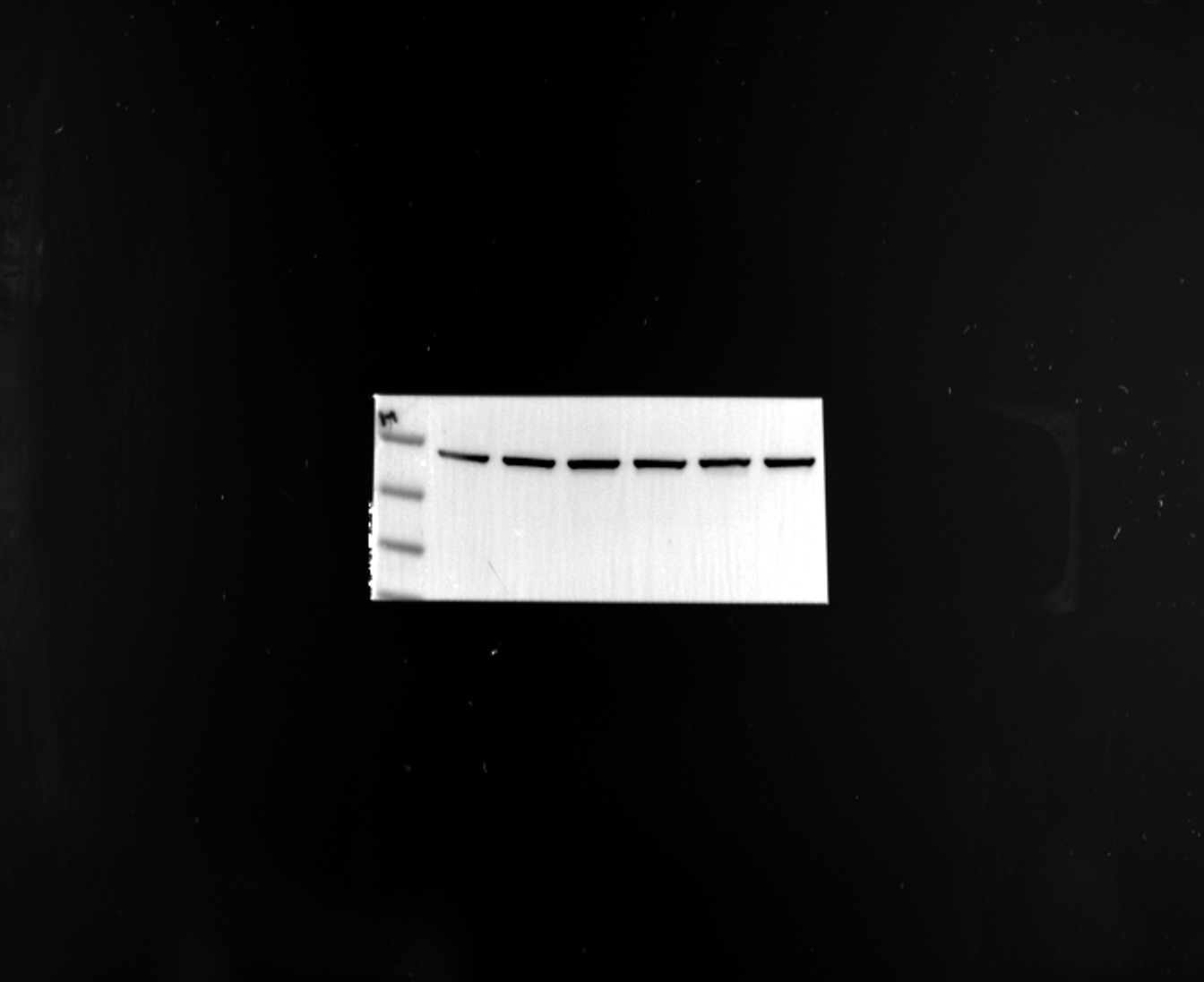

Supplement: Supplementary file 1 [file DataSheet1.zip › raw data traces-Figure 2E/STAT3/tubulin.Tif]

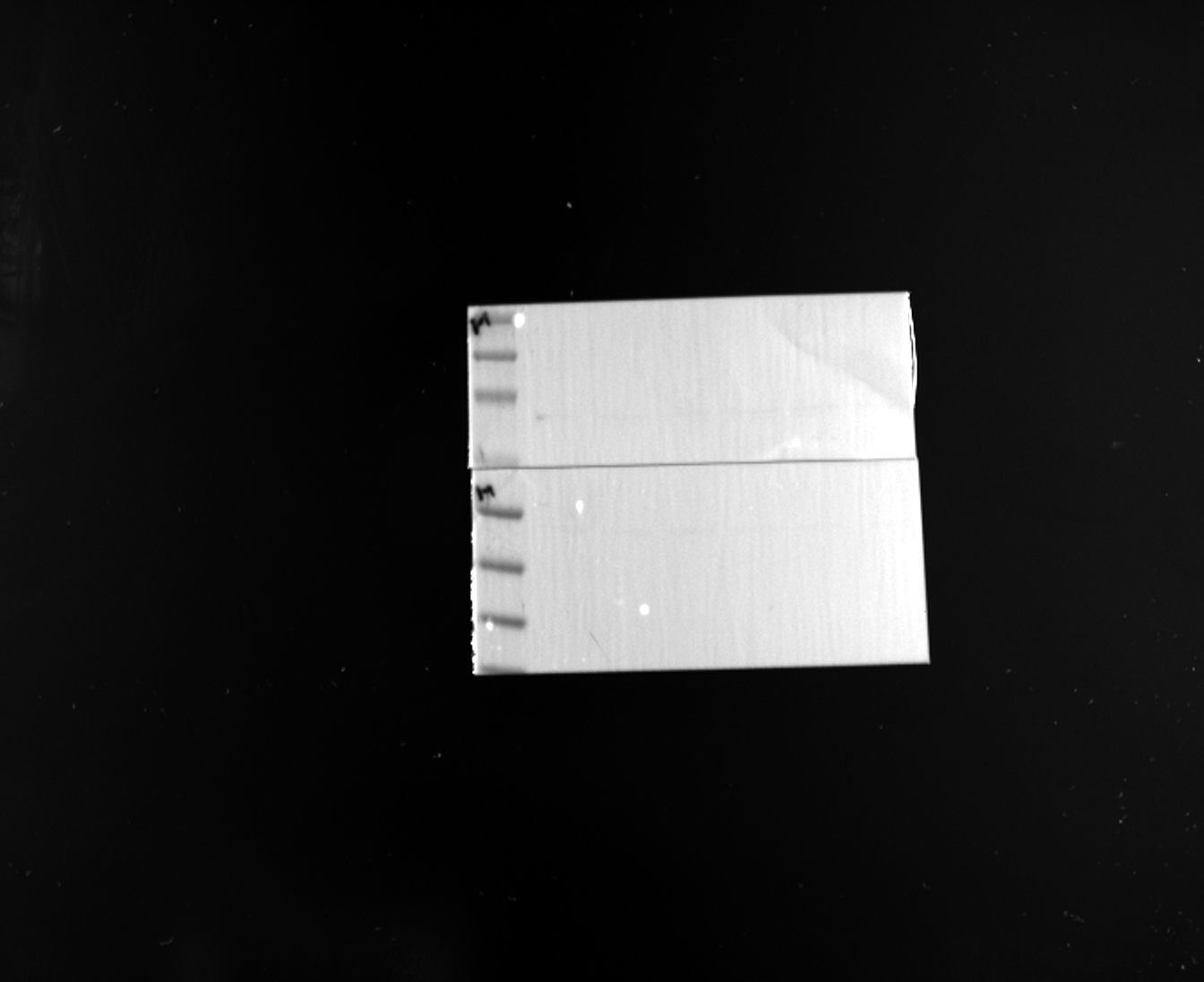

Supplement: Supplementary file 1 [file DataSheet1.zip › raw data traces-Figure 2E/STAT3/Uncropped images of blots.Tif]

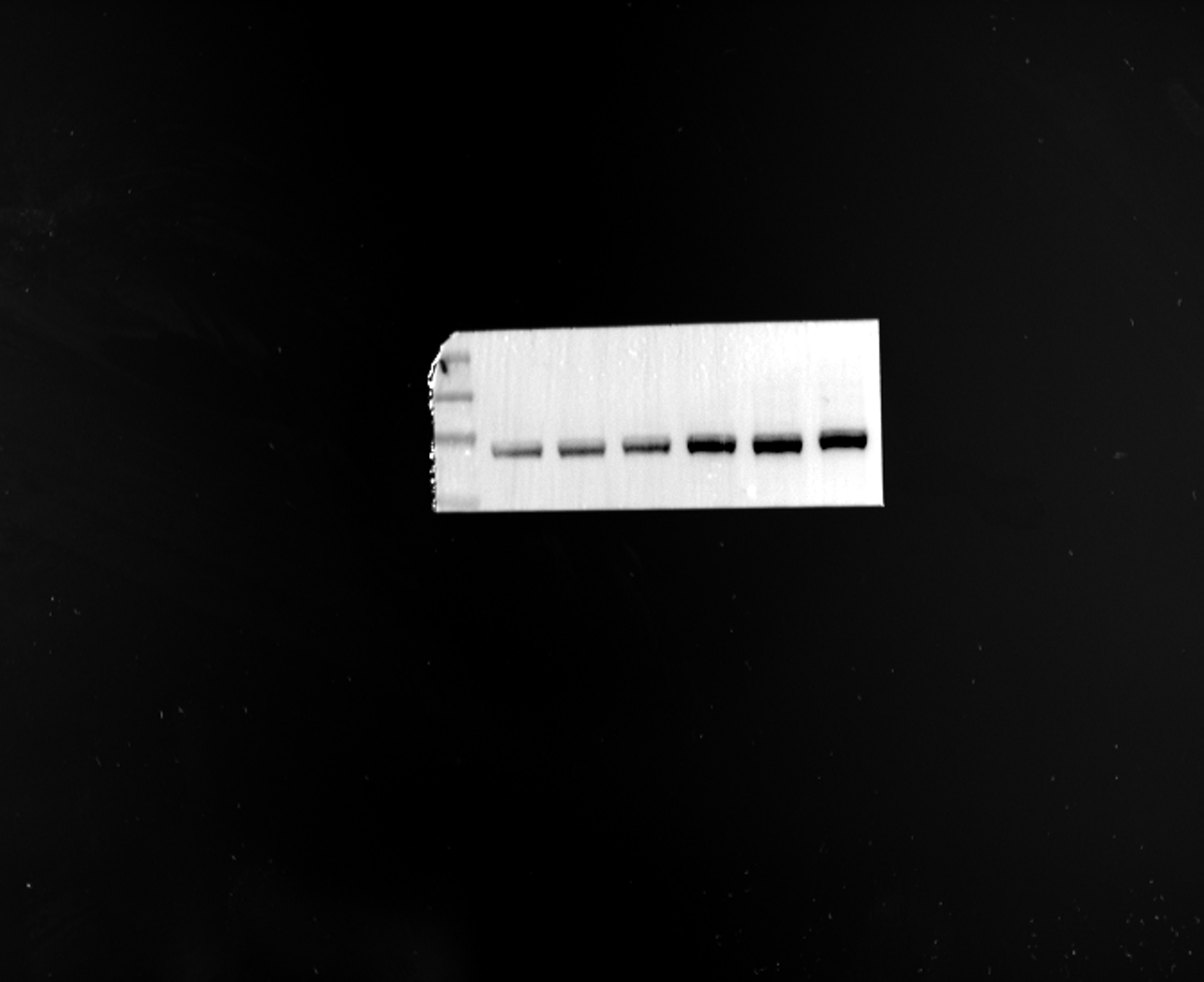

Supplement: Supplementary file 1 [file DataSheet1.zip › raw data traces-Figure 2E/TLR2/TLR2.Tif]

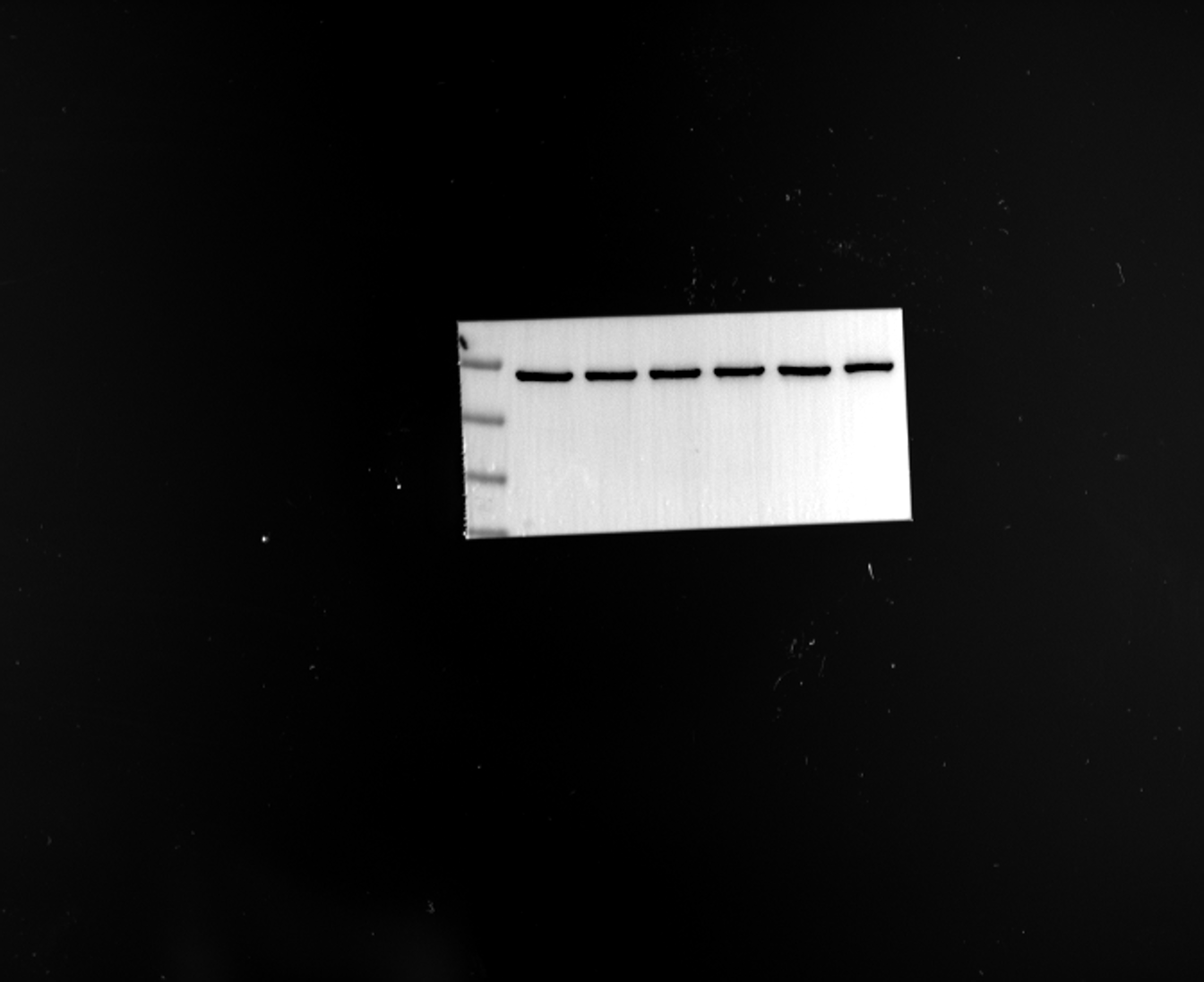

Supplement: Supplementary file 1 [file DataSheet1.zip › raw data traces-Figure 2E/TLR2/tubulin.Tif]

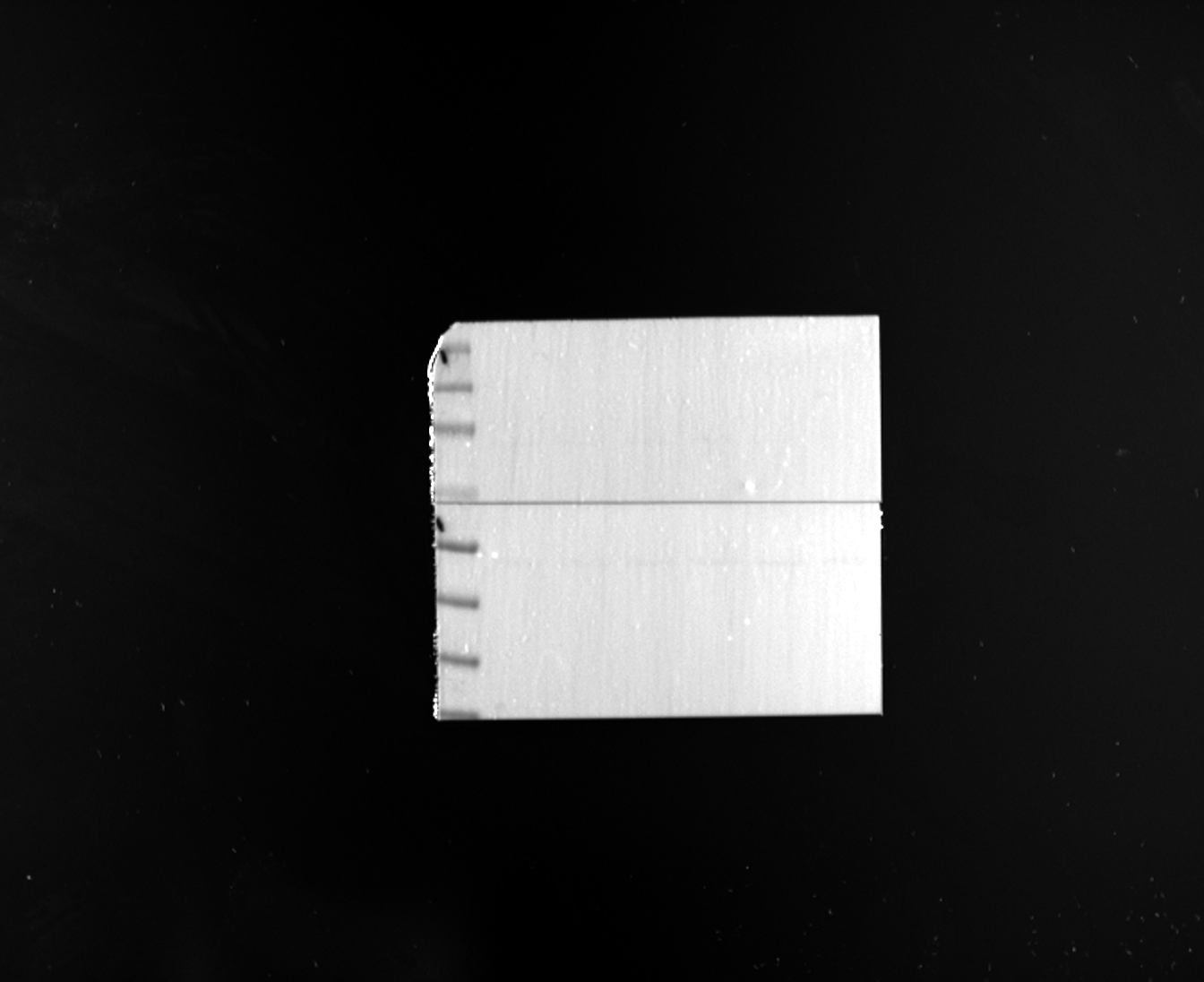

Supplement: Supplementary file 1 [file DataSheet1.zip › raw data traces-Figure 2E/TLR2/Uncropped images of blots.Tif]

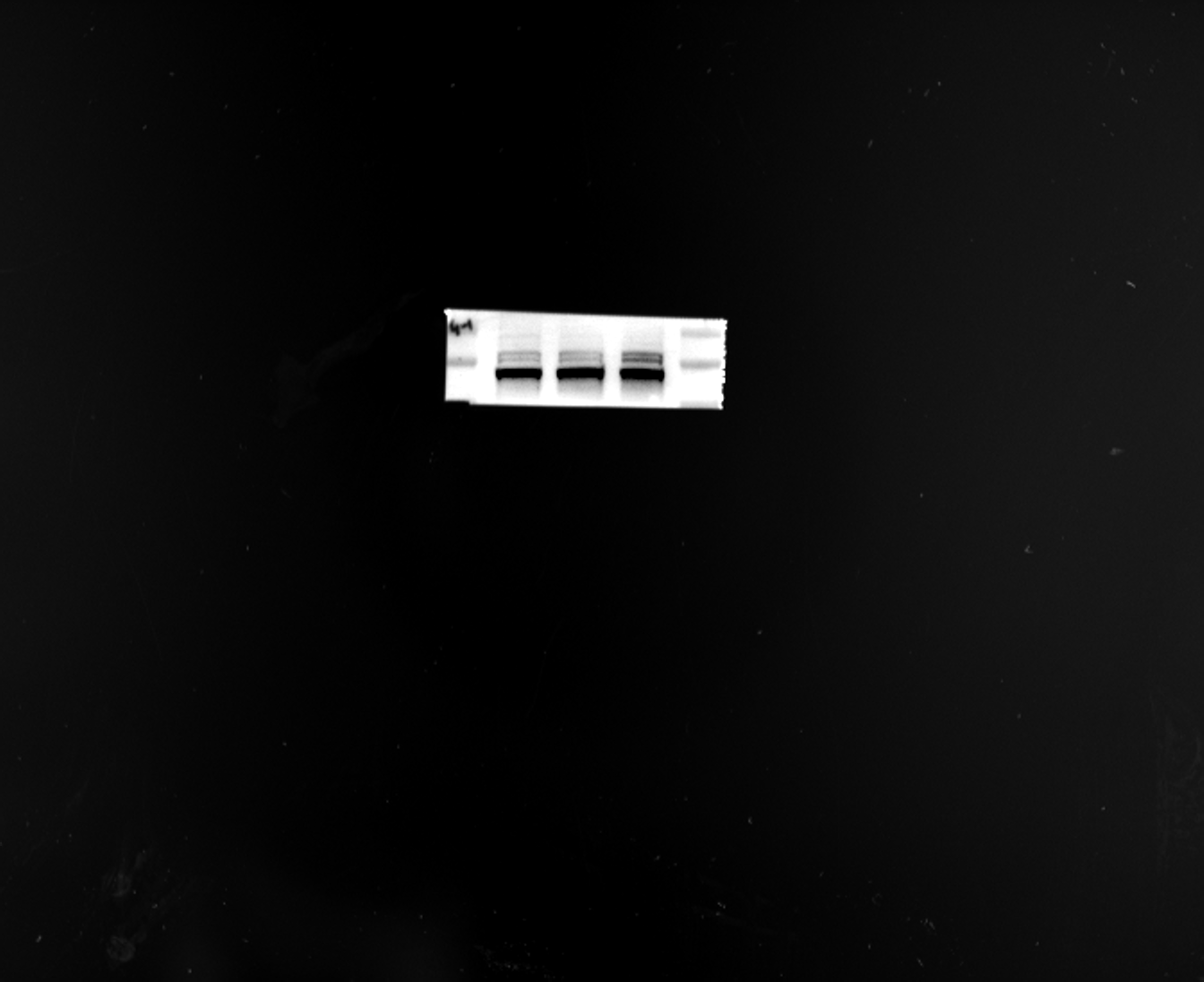

Supplement: Supplementary file 2 [file DataSheet2.zip › raw data traces-Figure 4E/JAK2/JAK2.Tif]

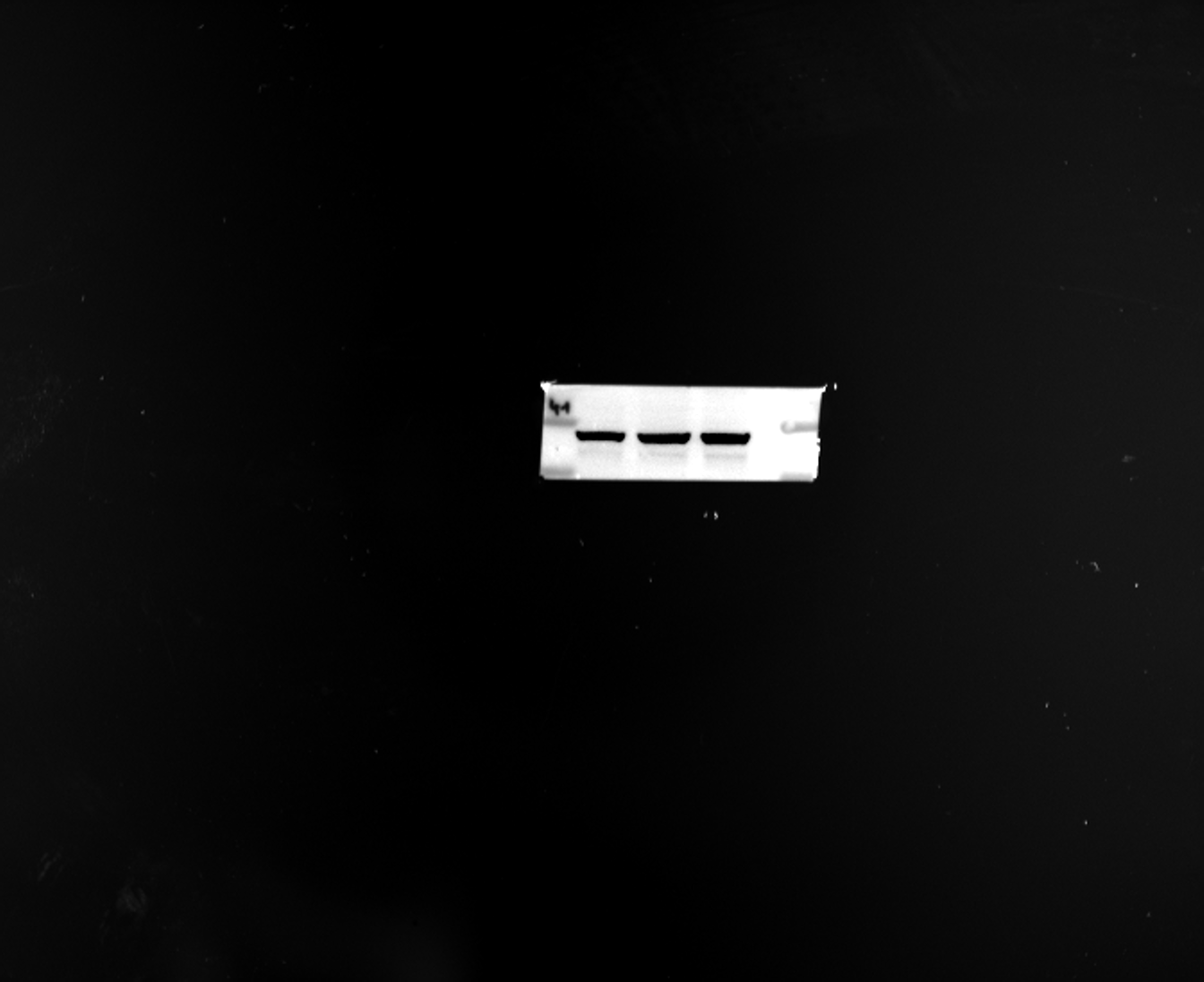

Supplement: Supplementary file 2 [file DataSheet2.zip › raw data traces-Figure 4E/JAK2/tubulin.Tif]

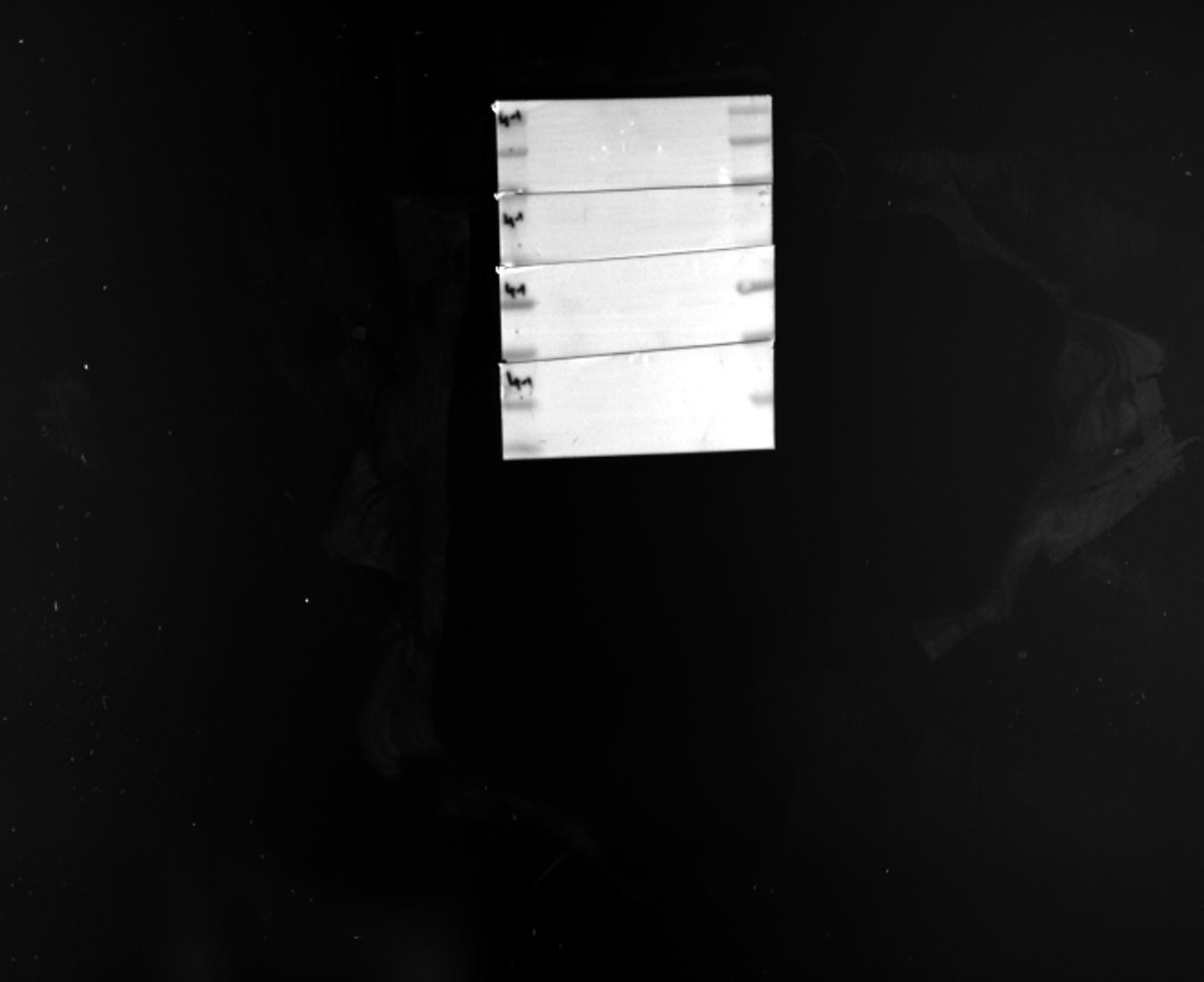

Supplement: Supplementary file 2 [file DataSheet2.zip › raw data traces-Figure 4E/JAK2/Uncropped images of blots.Tif]

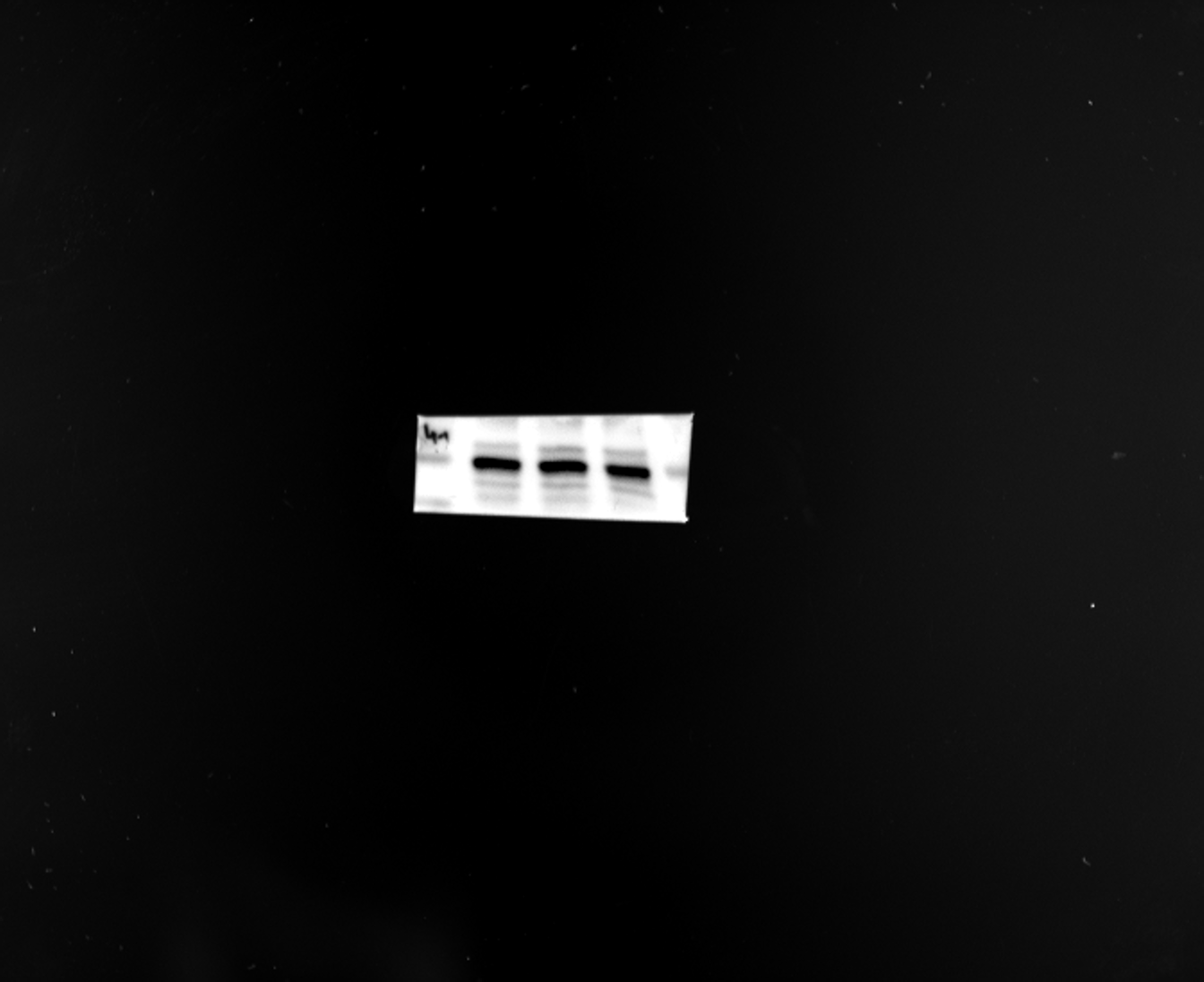

Supplement: Supplementary file 2 [file DataSheet2.zip › raw data traces-Figure 4E/MyD88/MyD88.Tif]

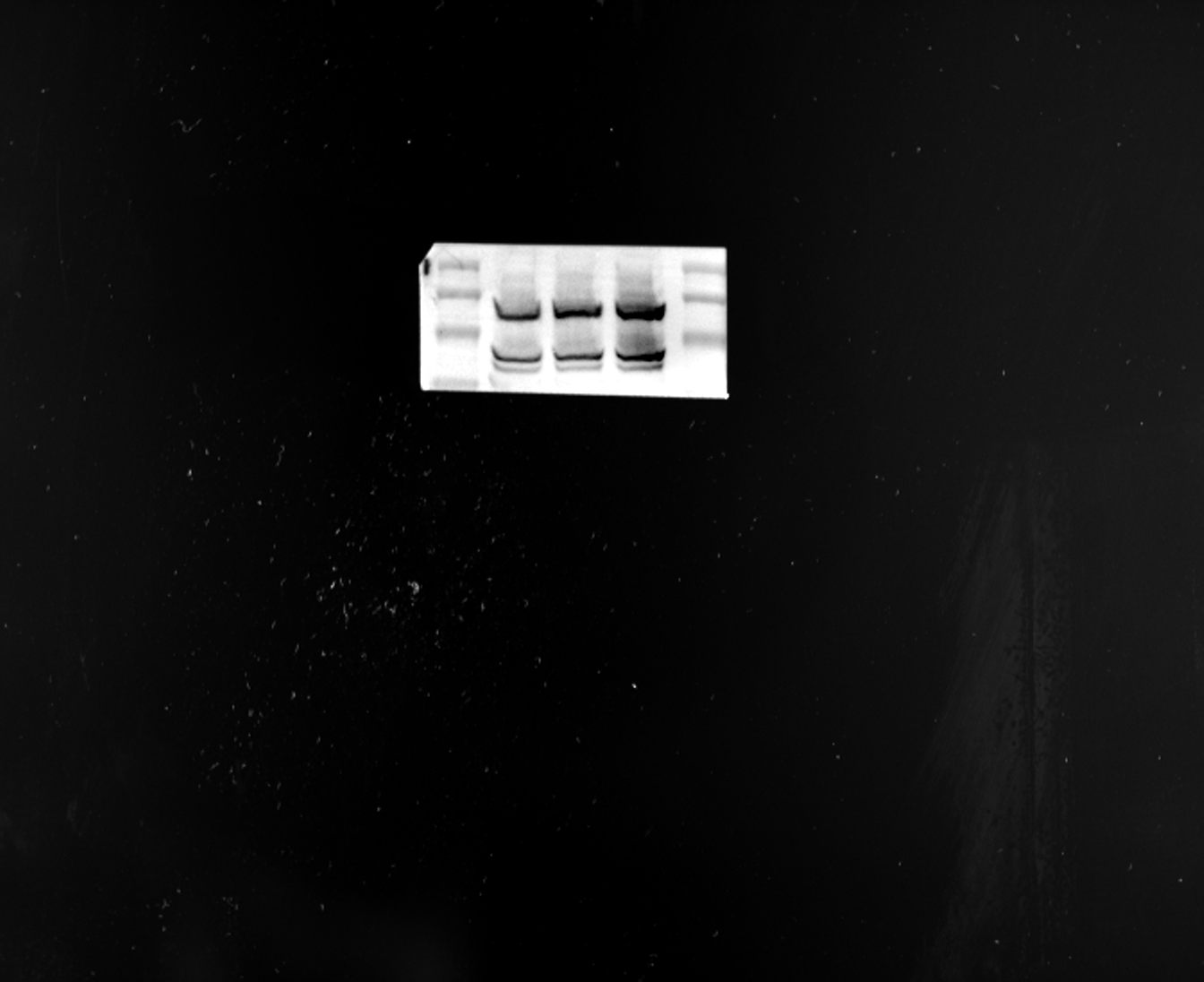

Supplement: Supplementary file 2 [file DataSheet2.zip › raw data traces-Figure 4E/P-JAK2/P-JAK2.Tif]

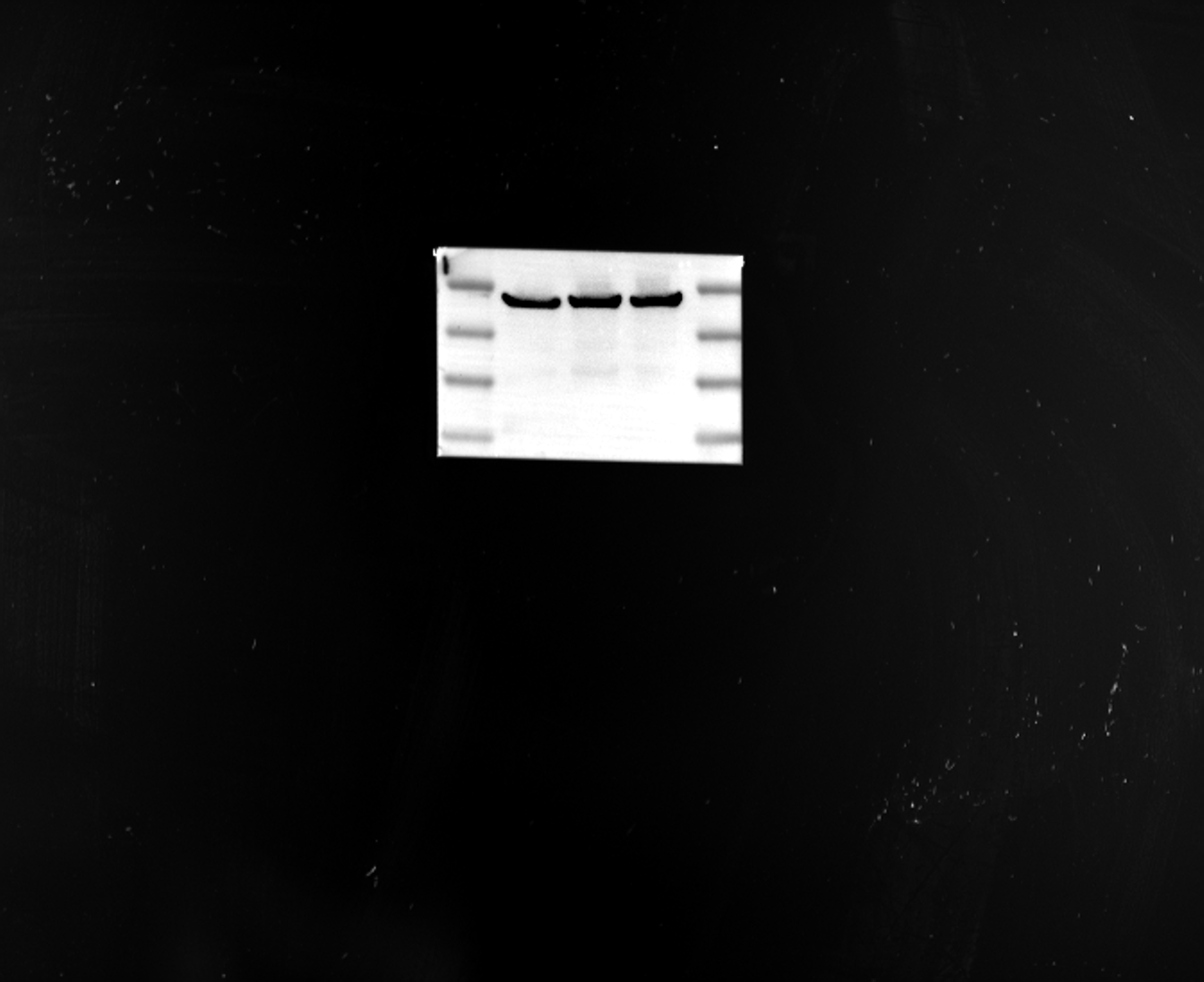

Supplement: Supplementary file 2 [file DataSheet2.zip › raw data traces-Figure 4E/P-JAK2/tubulin.Tif]

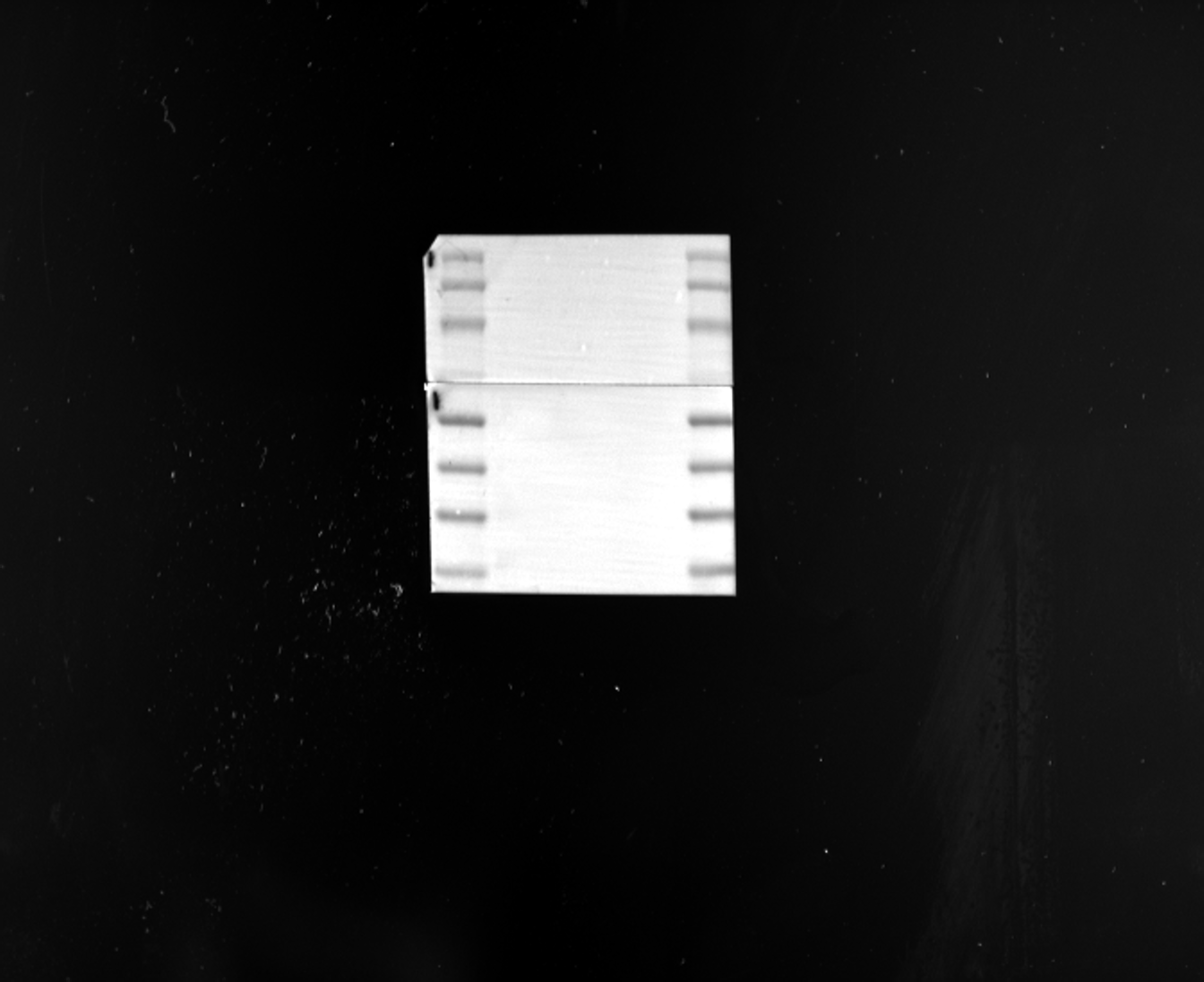

Supplement: Supplementary file 2 [file DataSheet2.zip › raw data traces-Figure 4E/P-JAK2/Uncropped images of blots.Tif]

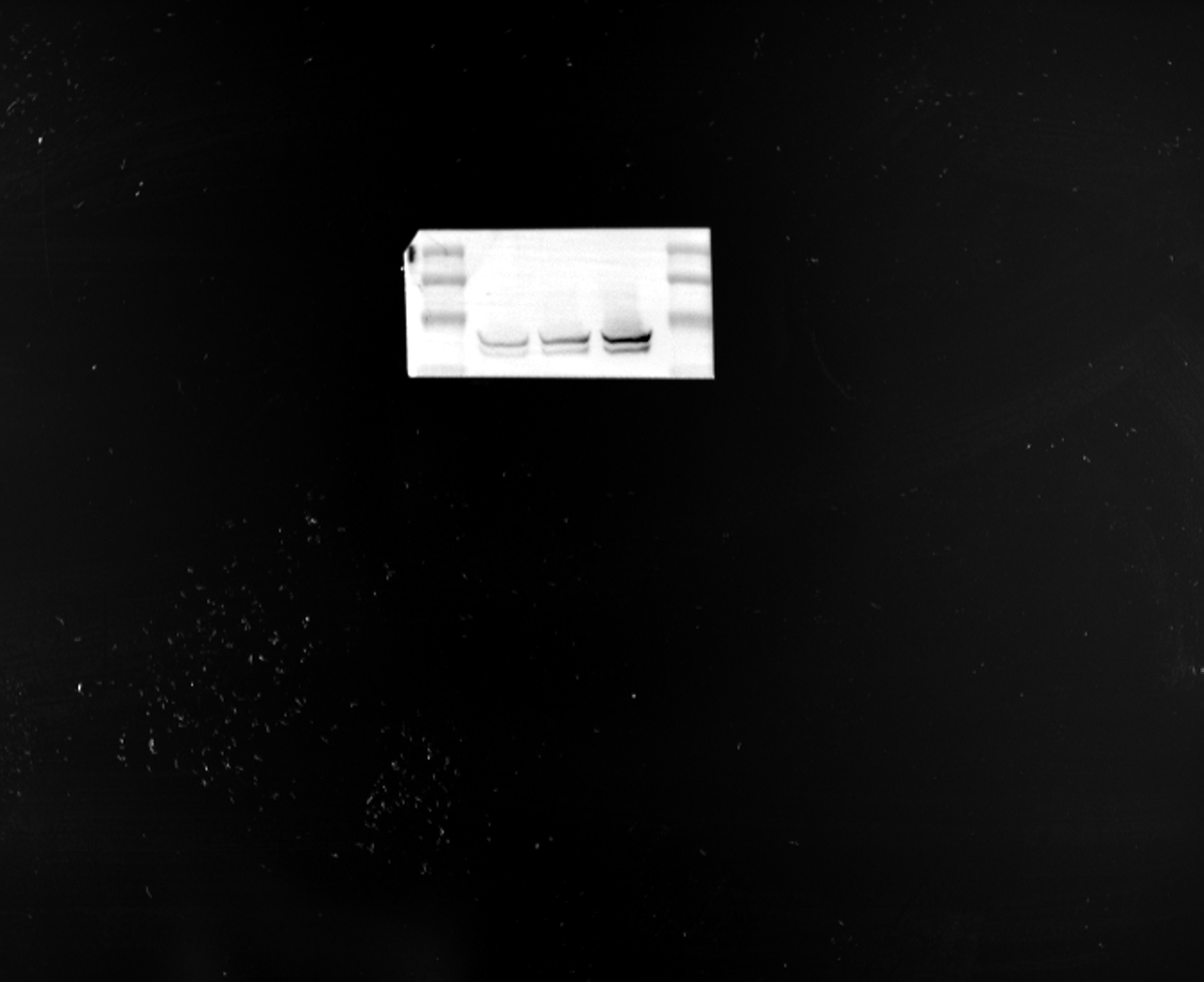

Supplement: Supplementary file 2 [file DataSheet2.zip › raw data traces-Figure 4E/p-STAT3/p-STAT3.Tif]

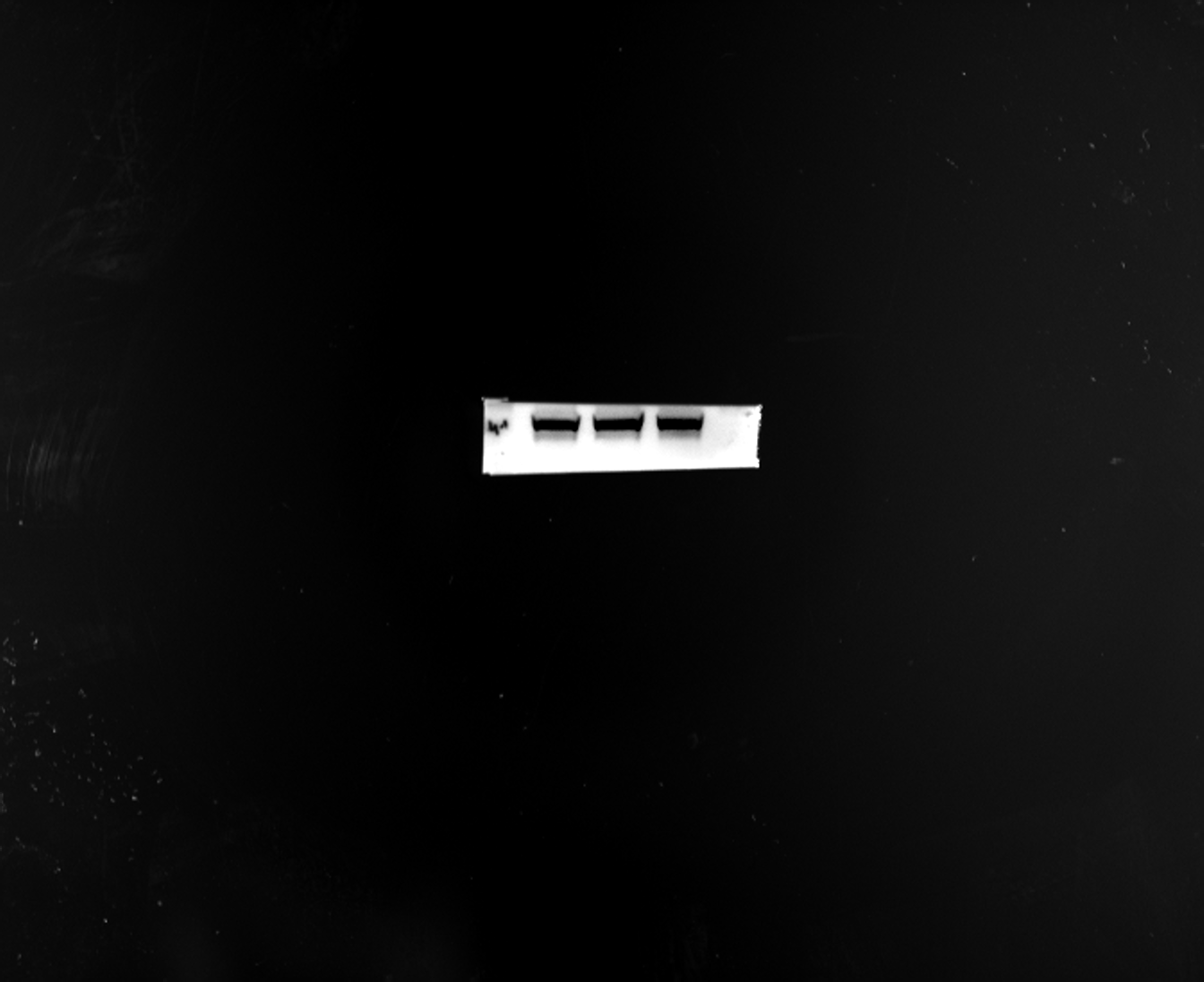

Supplement: Supplementary file 2 [file DataSheet2.zip › raw data traces-Figure 4E/STAT3/STAT3.Tif]

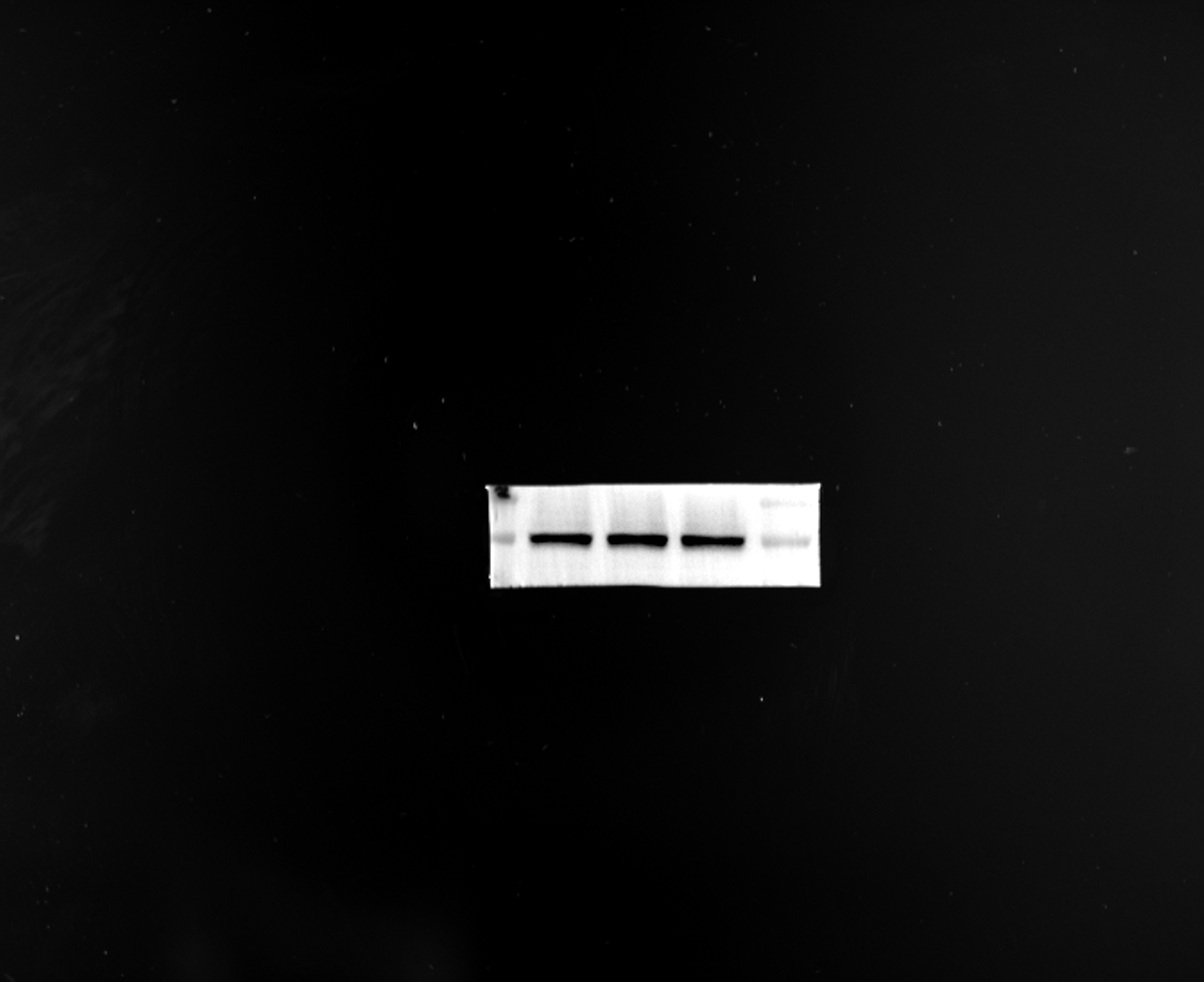

Supplement: Supplementary file 2 [file DataSheet2.zip › raw data traces-Figure 4E/TLR2/TLR2.Tif]

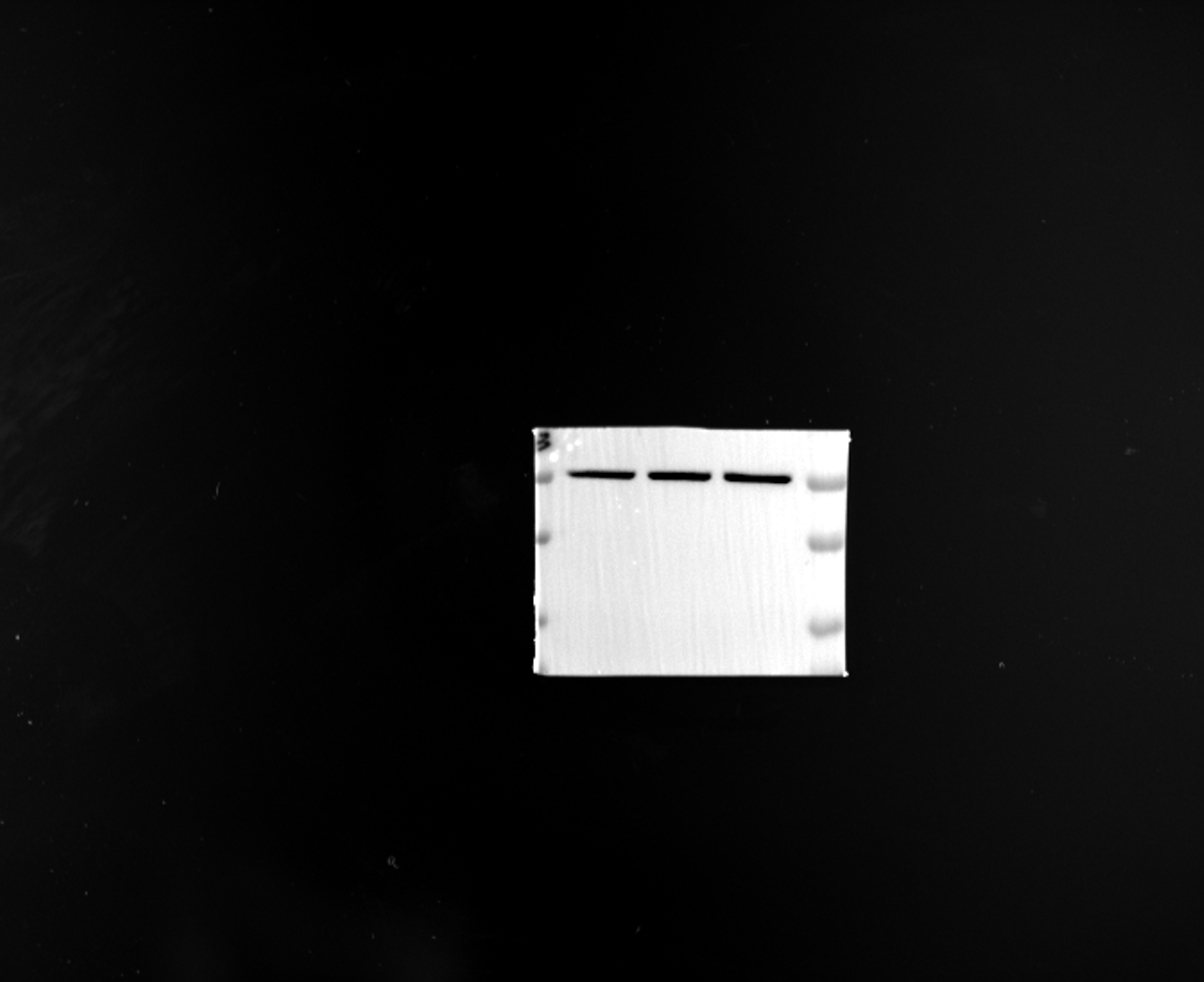

Supplement: Supplementary file 2 [file DataSheet2.zip › raw data traces-Figure 4E/TLR2/tubulin.Tif]

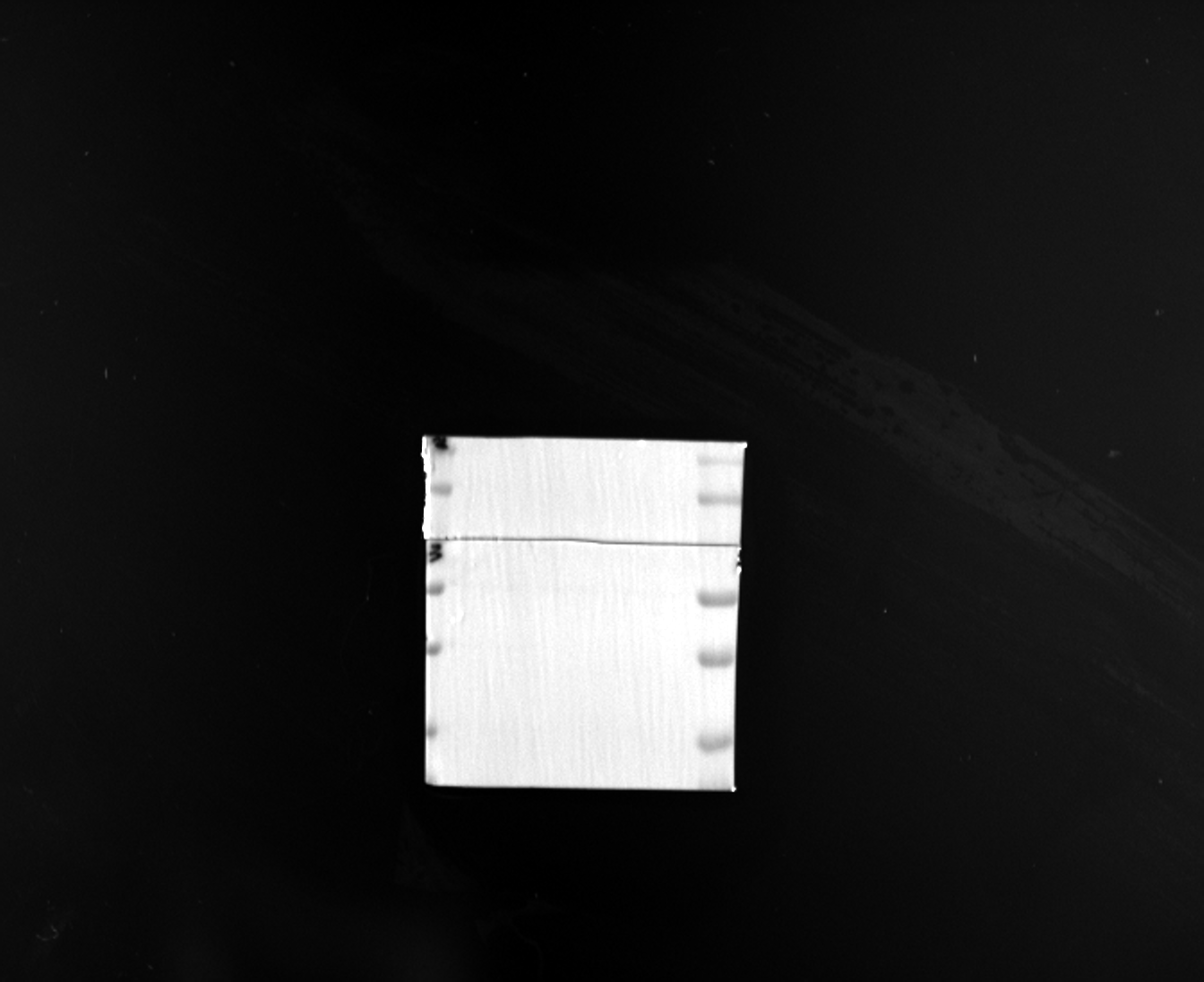

Supplement: Supplementary file 2 [file DataSheet2.zip › raw data traces-Figure 4E/TLR2/Uncropped images of blots.Tif]

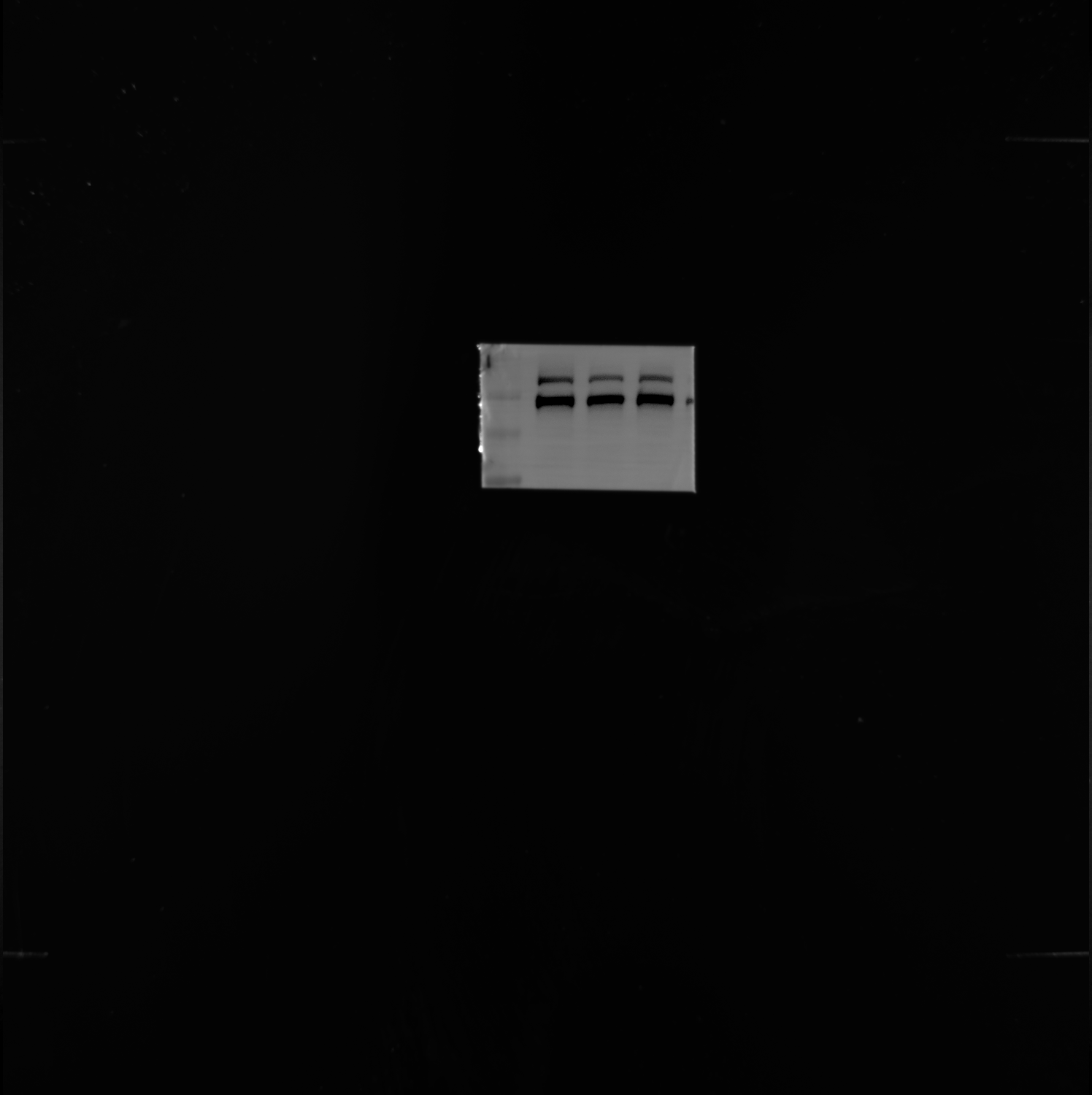

Supplement: Supplementary file 3 [file DataSheet3.zip › raw data traces-Figure 4F/JAK2/JAK2.tiff]

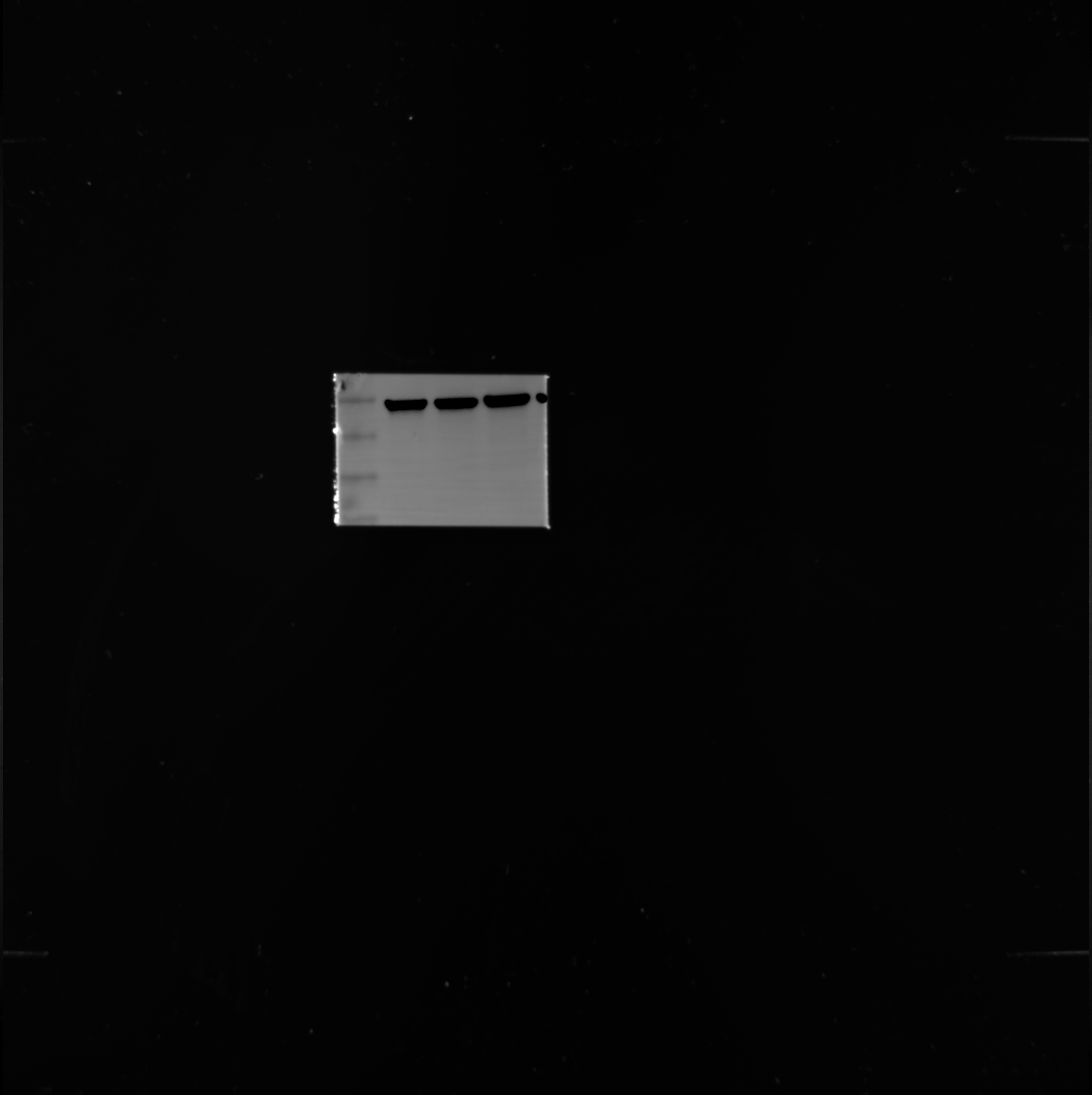

Supplement: Supplementary file 3 [file DataSheet3.zip › raw data traces-Figure 4F/JAK2/tubulin.tiff]

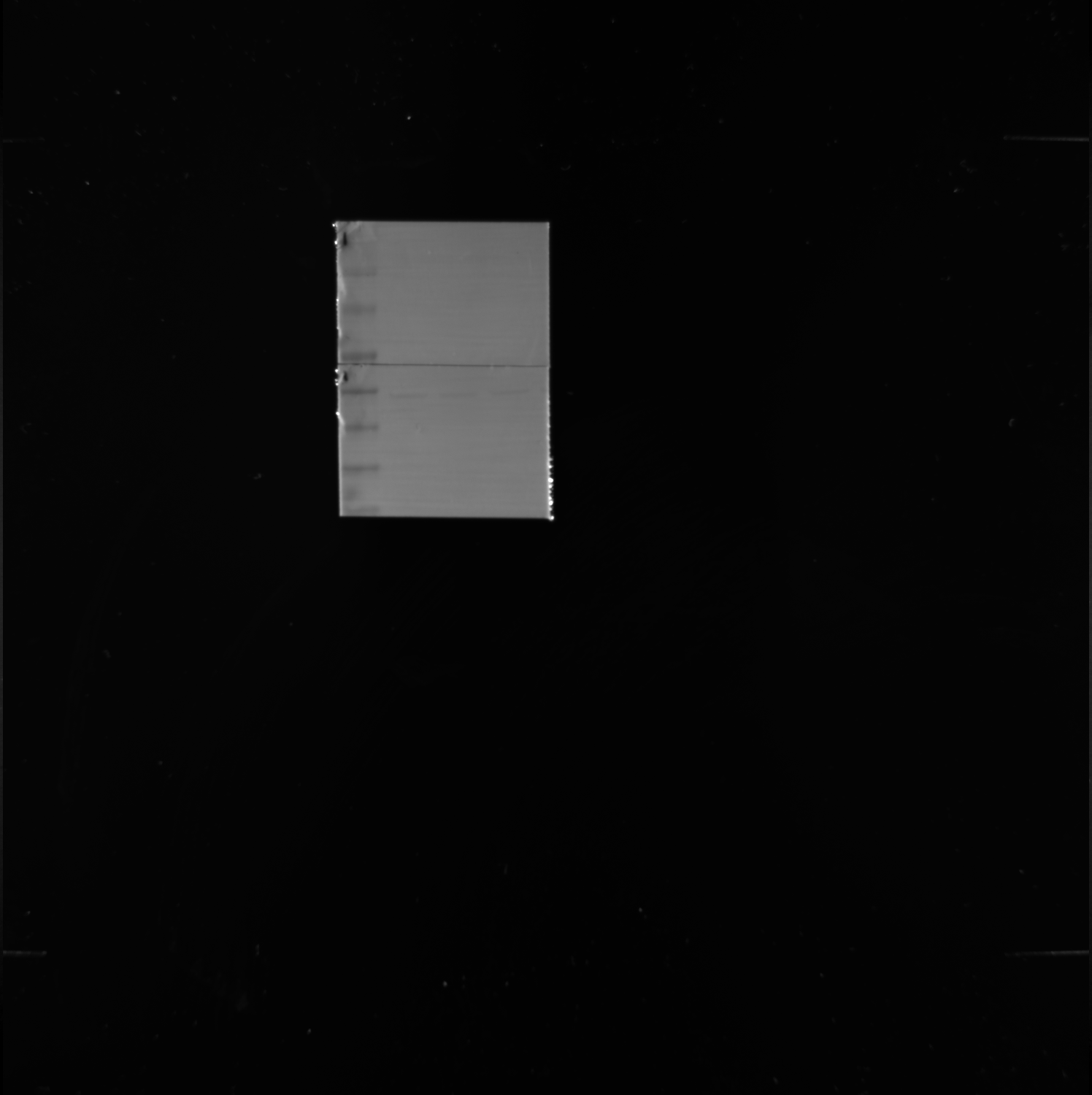

Supplement: Supplementary file 3 [file DataSheet3.zip › raw data traces-Figure 4F/JAK2/Uncropped images of blots.tiff]

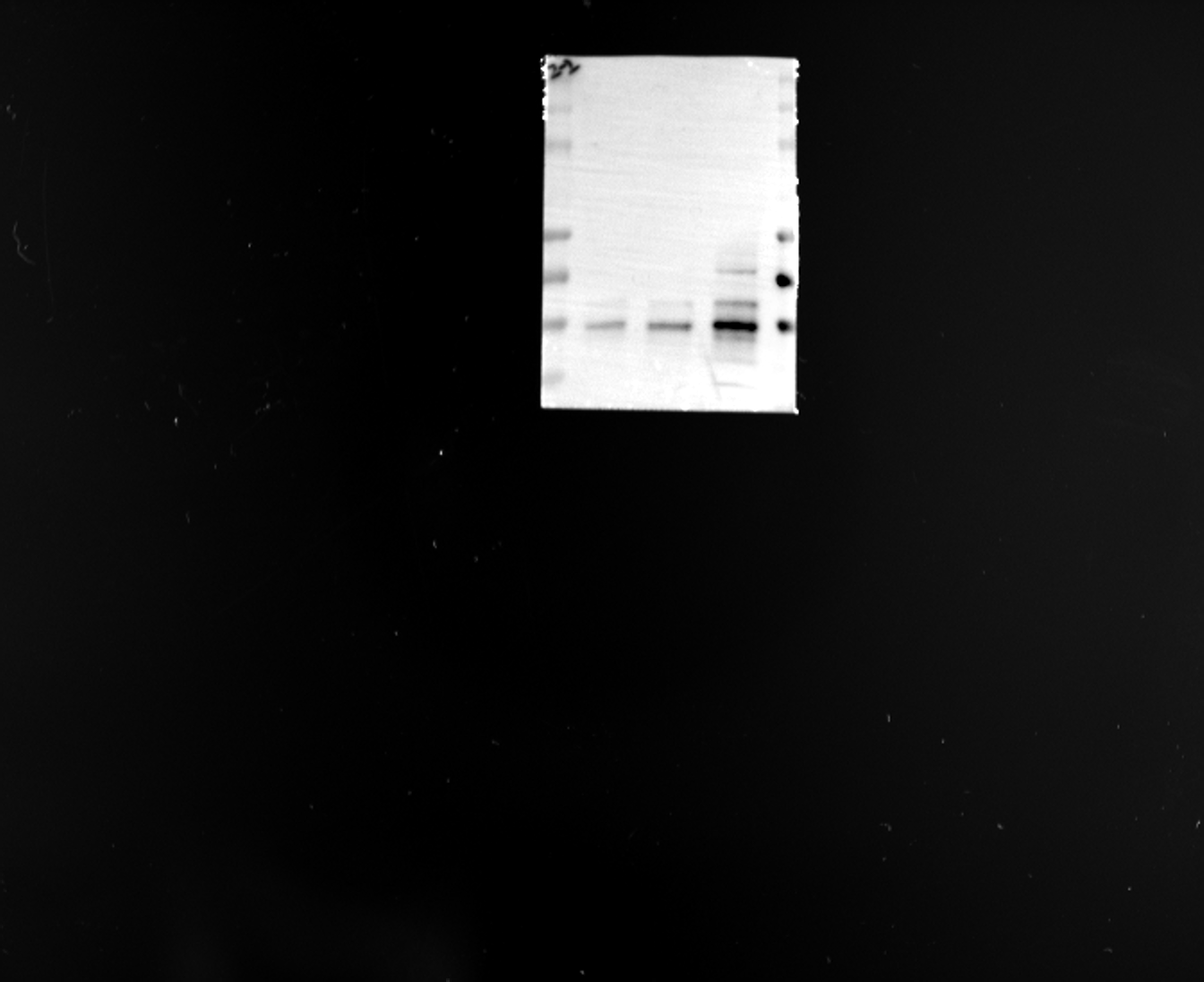

Supplement: Supplementary file 3 [file DataSheet3.zip › raw data traces-Figure 4F/MyD88/MyD88.Tif]

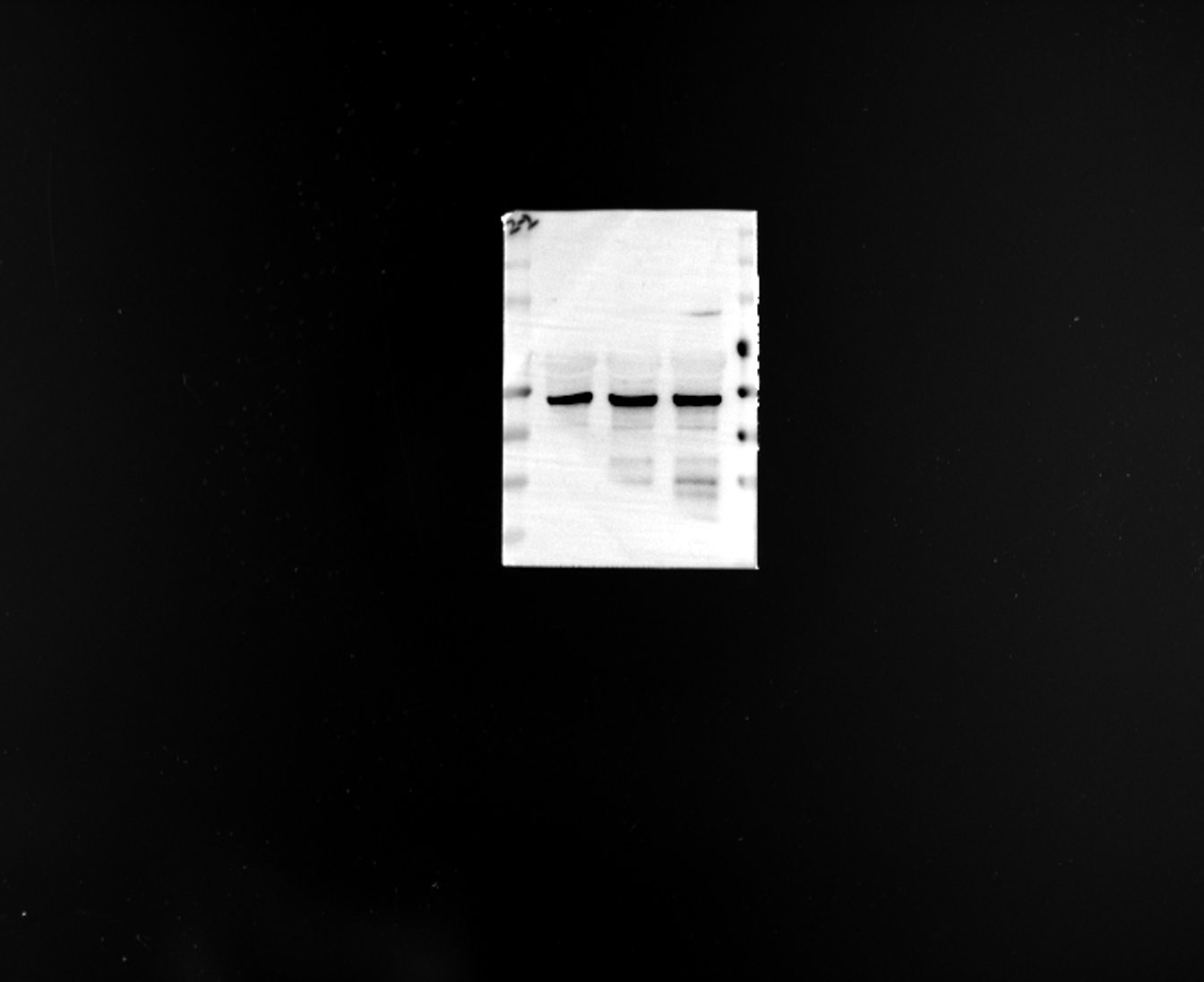

Supplement: Supplementary file 3 [file DataSheet3.zip › raw data traces-Figure 4F/MyD88/tubulin.Tif]

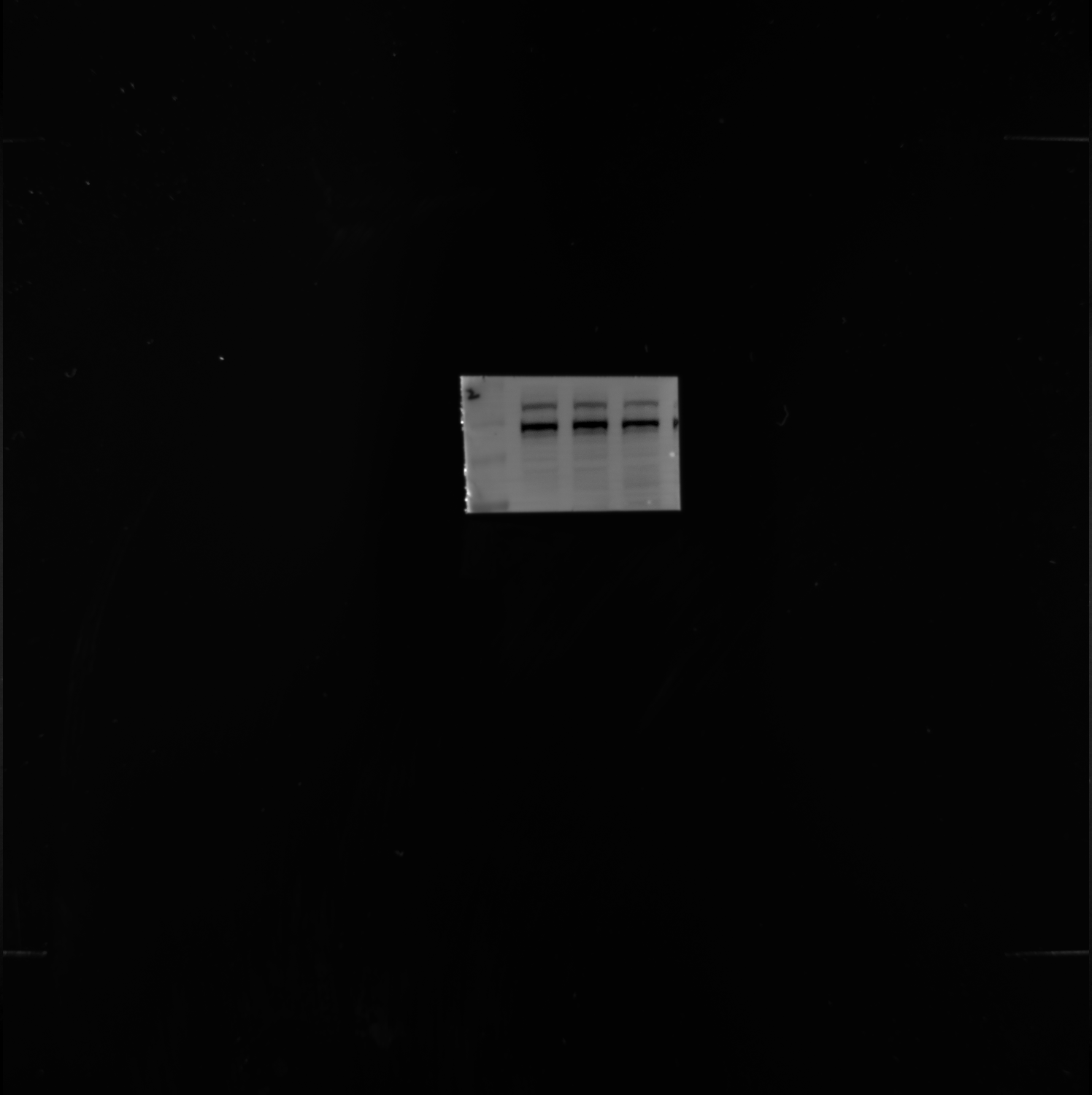

Supplement: Supplementary file 3 [file DataSheet3.zip › raw data traces-Figure 4F/p-JAK2/p-JAK2.tiff]

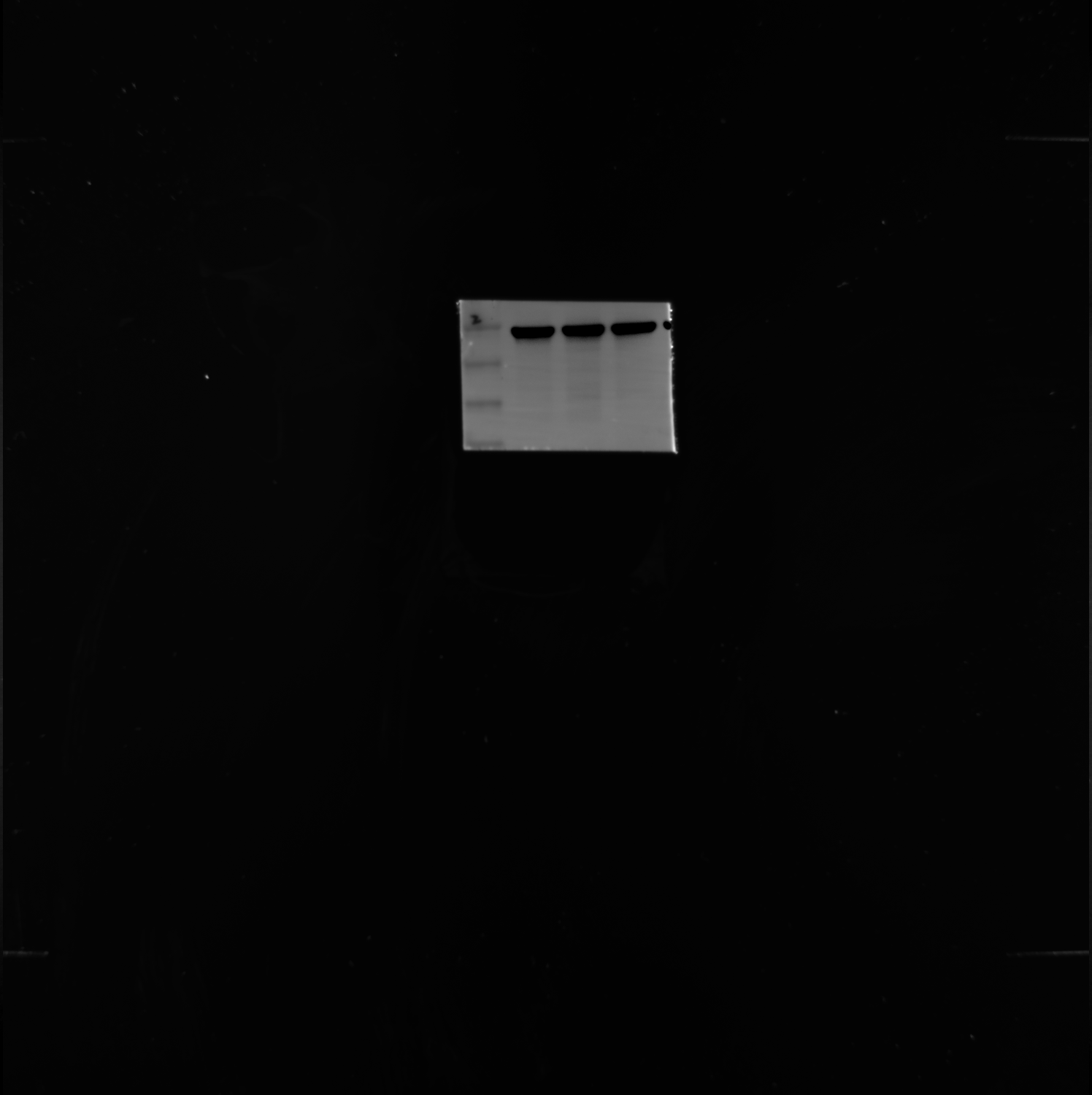

Supplement: Supplementary file 3 [file DataSheet3.zip › raw data traces-Figure 4F/p-JAK2/tubulin.tiff]

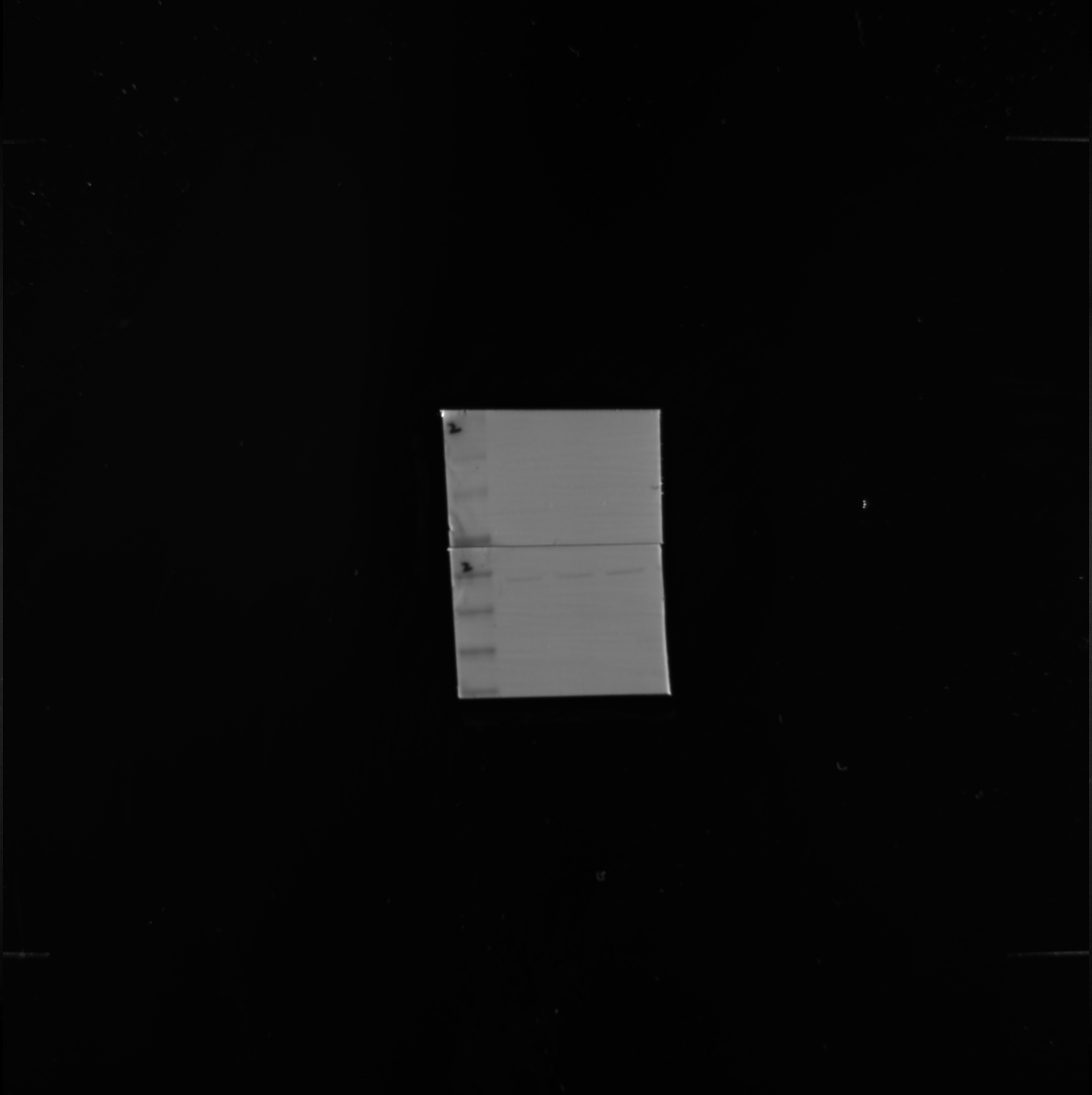

Supplement: Supplementary file 3 [file DataSheet3.zip › raw data traces-Figure 4F/p-JAK2/Uncropped images of blots.tiff]

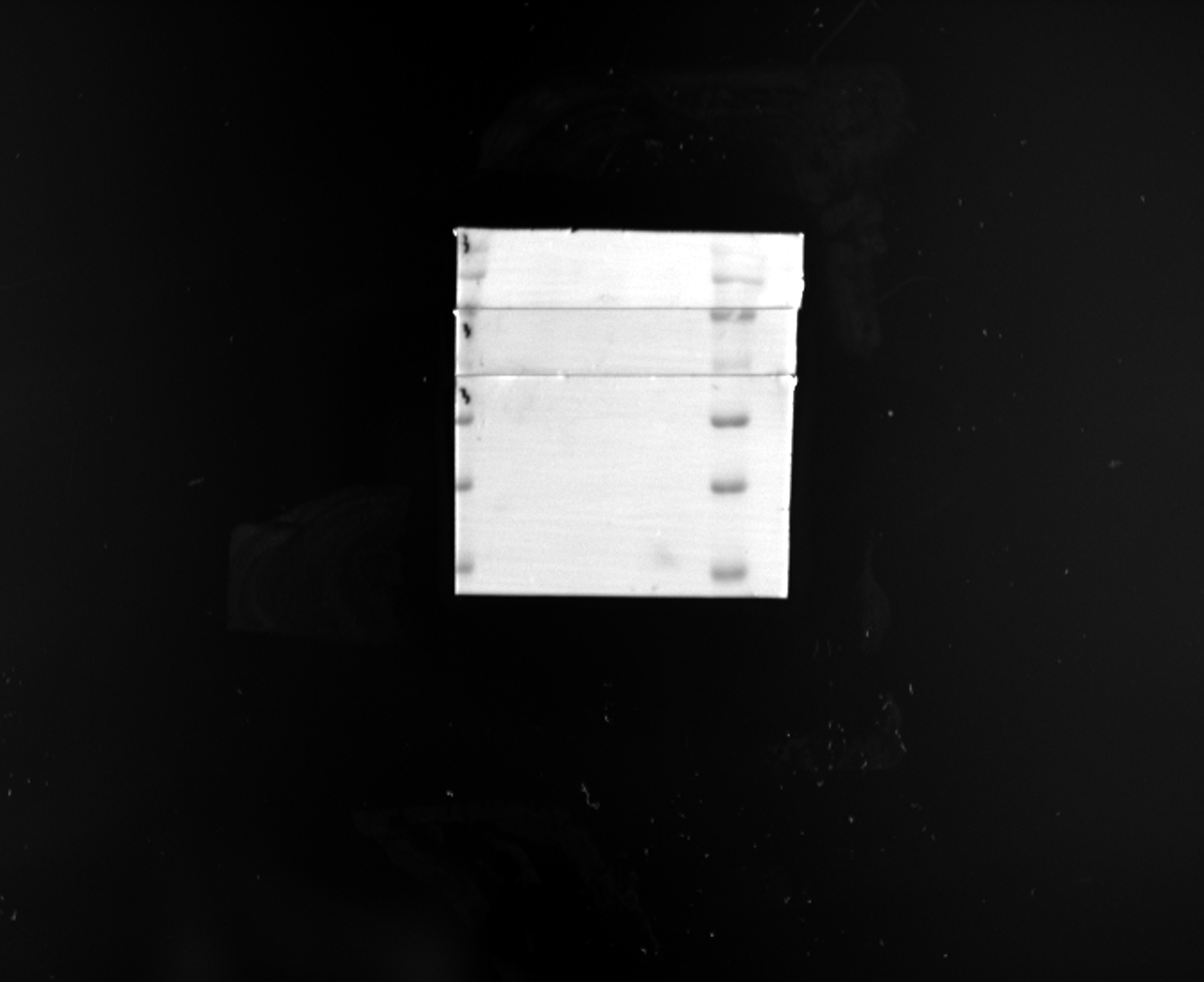

Supplement: Supplementary file 3 [file DataSheet3.zip › raw data traces-Figure 4F/P-STAT3/3-全膜.Tif]

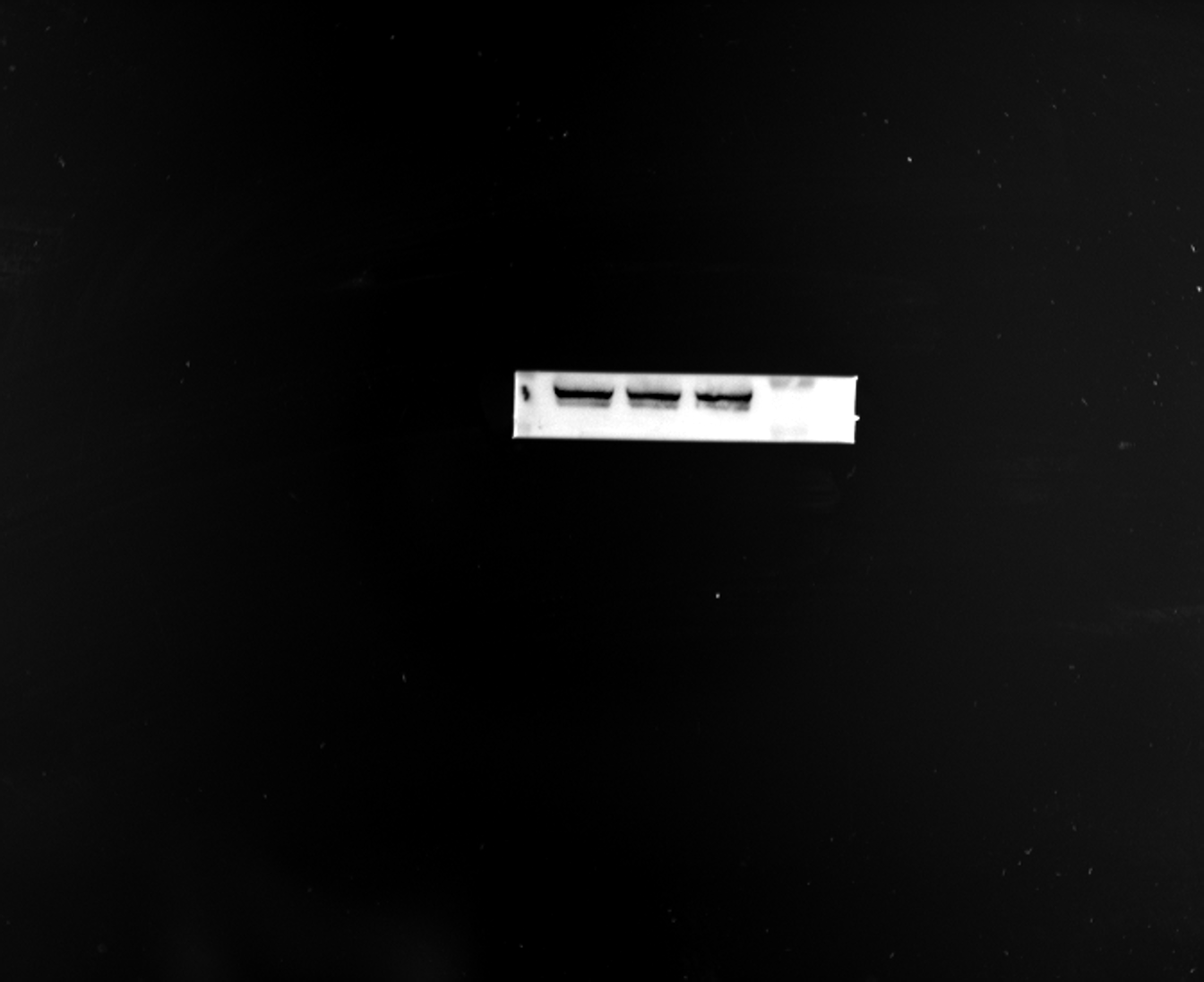

Supplement: Supplementary file 3 [file DataSheet3.zip › raw data traces-Figure 4F/P-STAT3/p-STAT3.Tif]

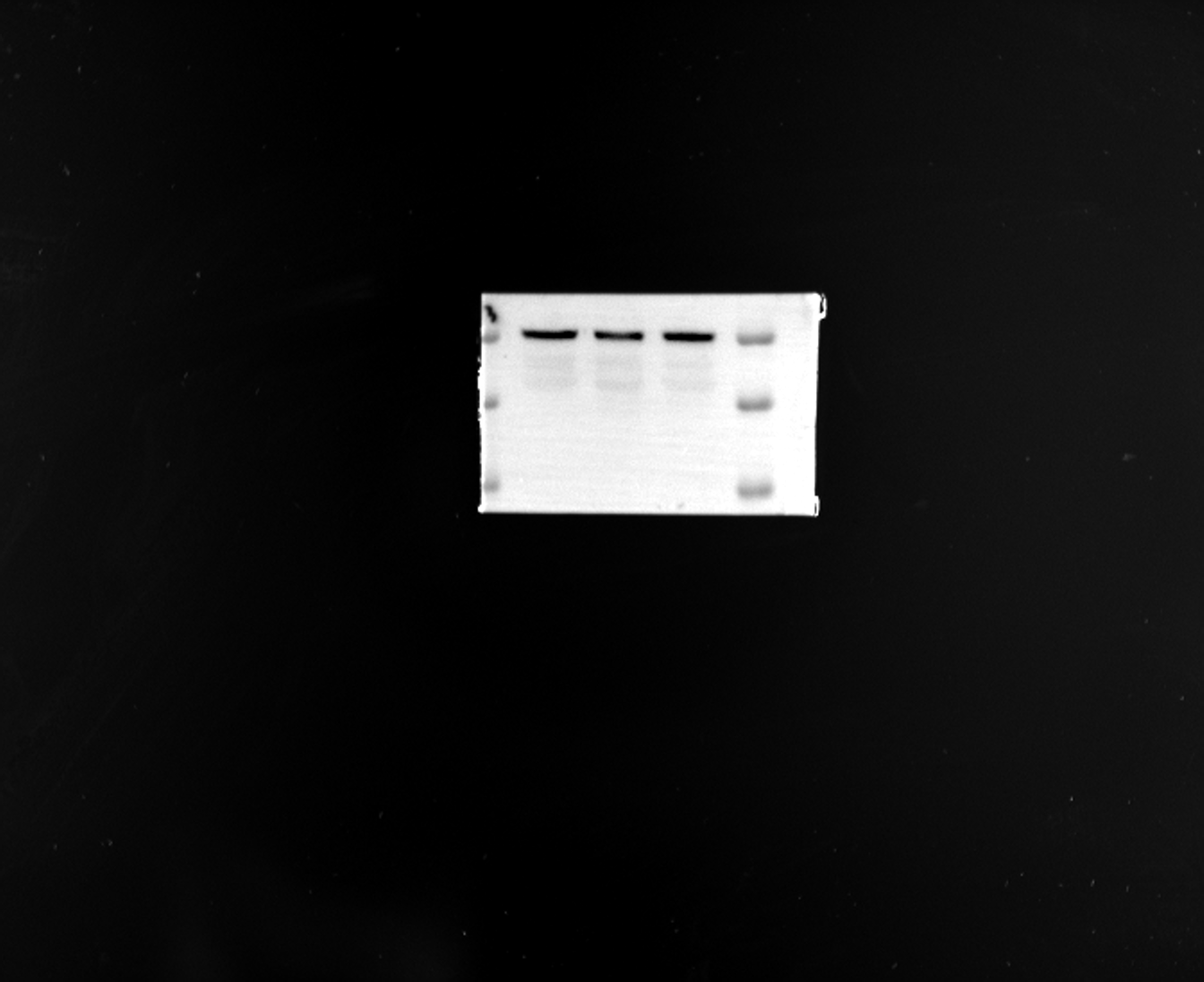

Supplement: Supplementary file 3 [file DataSheet3.zip › raw data traces-Figure 4F/P-STAT3/tubulin.Tif]

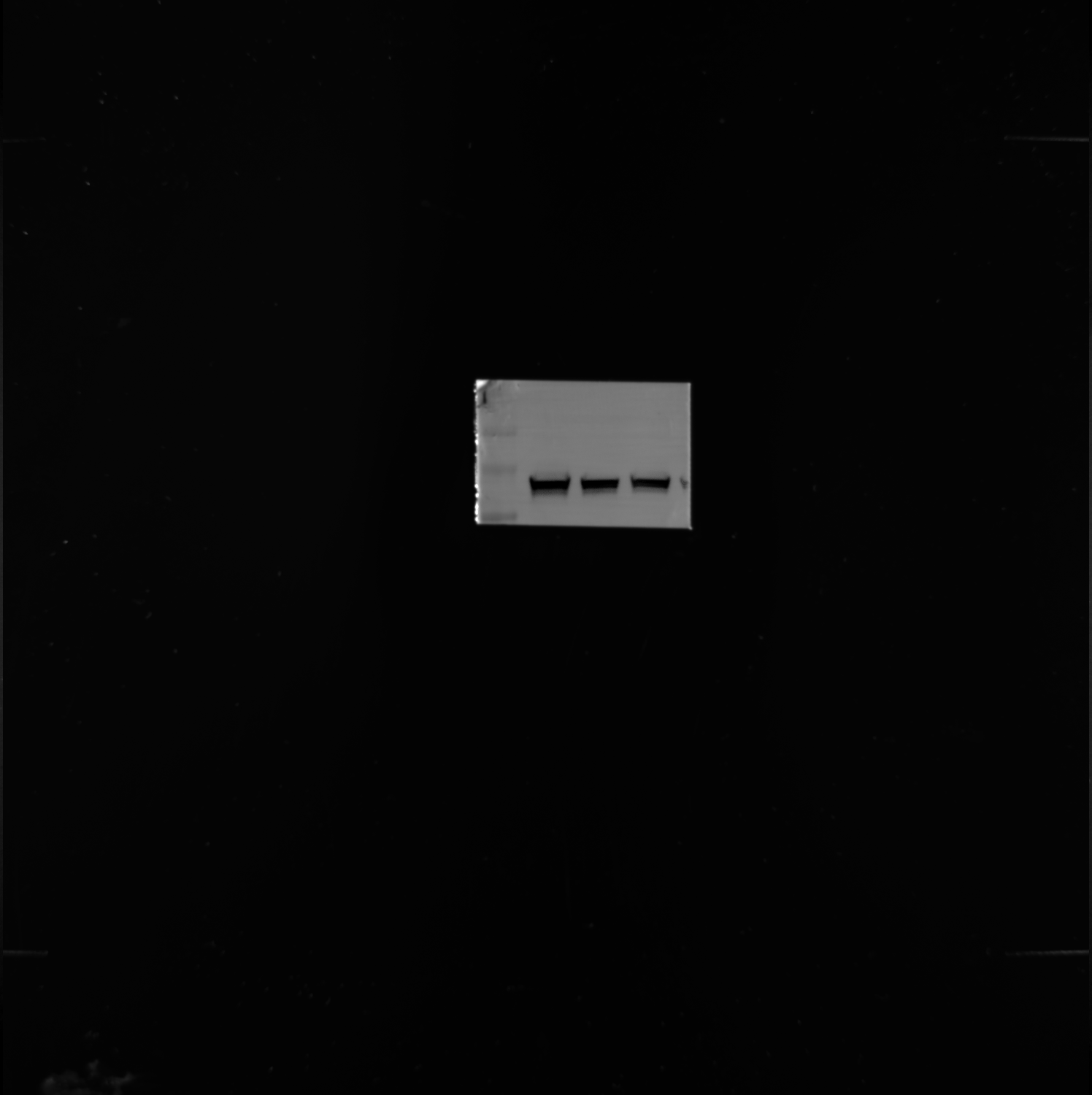

Supplement: Supplementary file 3 [file DataSheet3.zip › raw data traces-Figure 4F/STAT3/STAT3.tiff]

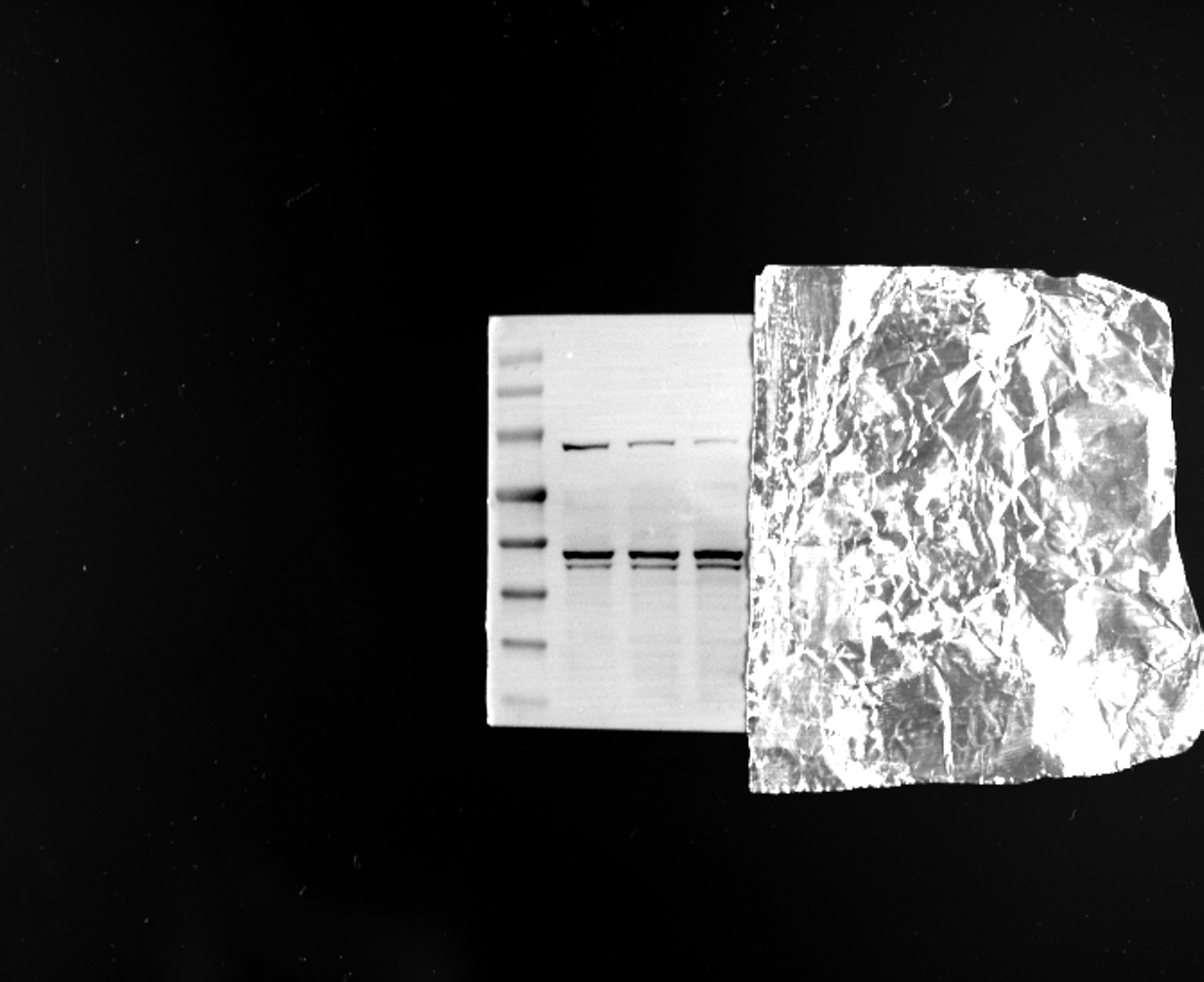

Supplement: Supplementary file 3 [file DataSheet3.zip › raw data traces-Figure 4F/TLR2/1-1tubuu.Tif]

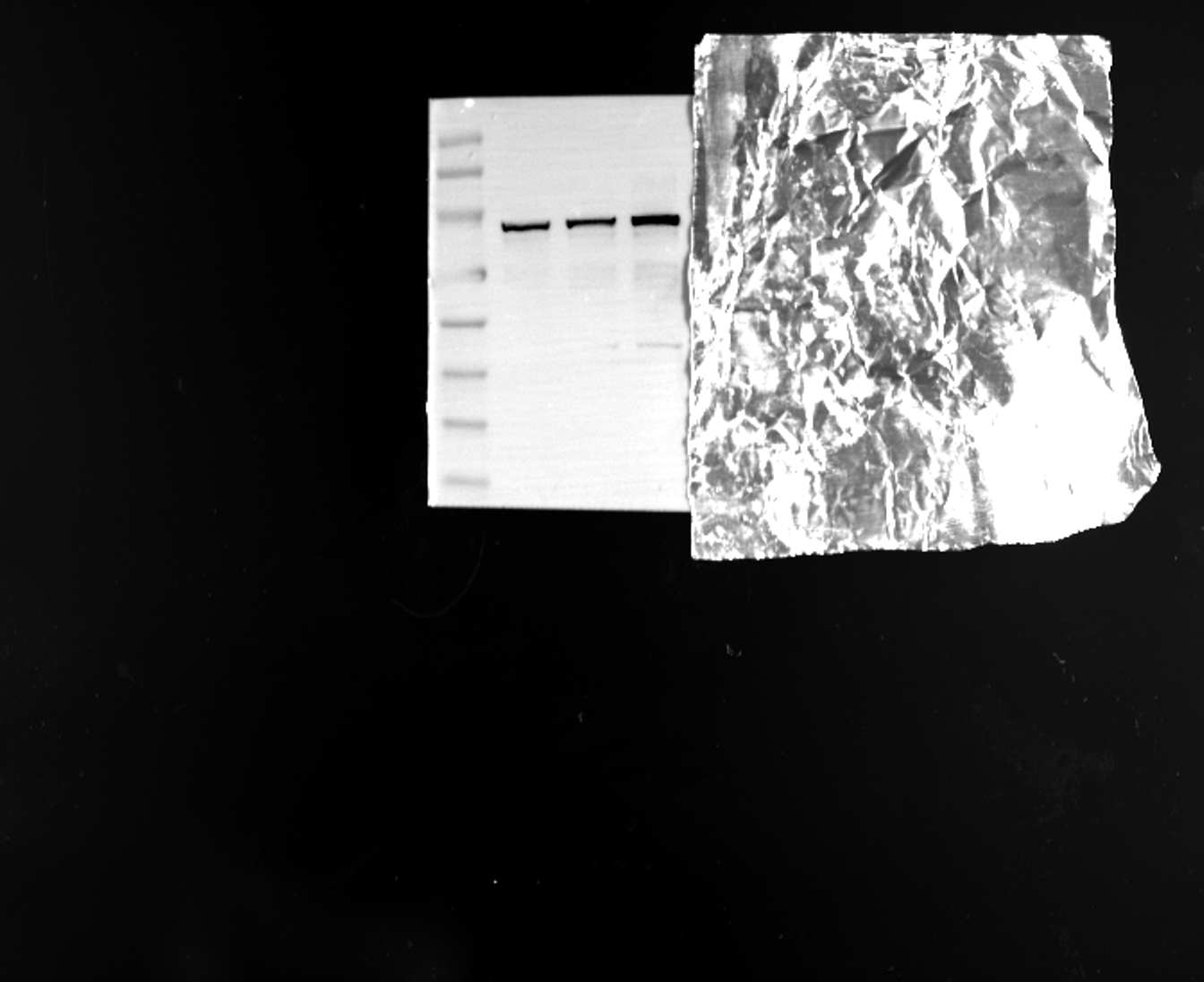

Supplement: Supplementary file 3 [file DataSheet3.zip › raw data traces-Figure 4F/TLR2/TLR2.Tif]

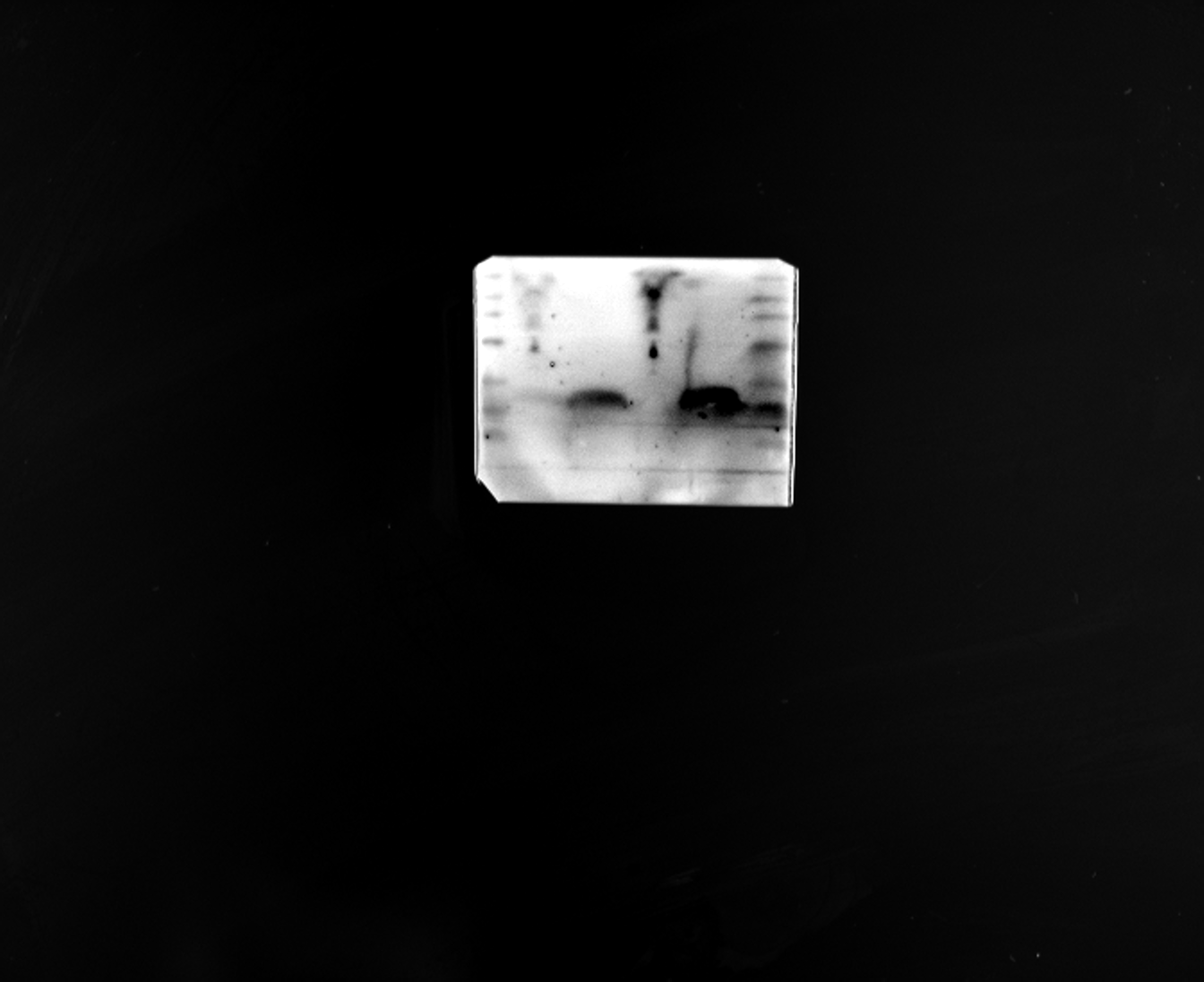

Supplement: Supplementary file 4 [file DataSheet4.zip › raw data traces-Figure 5C/Flag.Tif]

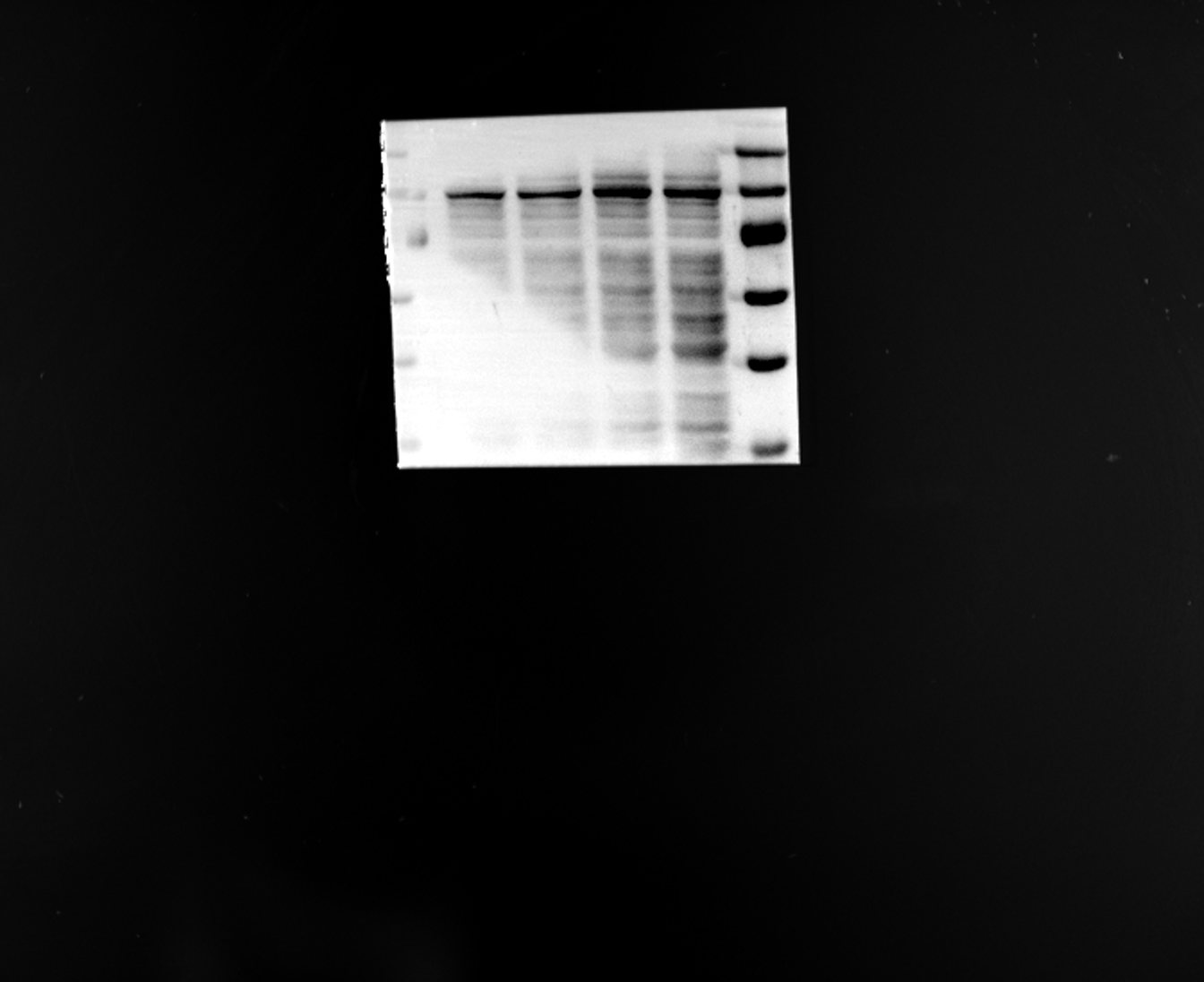

Supplement: Supplementary file 4 [file DataSheet4.zip › raw data traces-Figure 5C/TLR2.Tif]

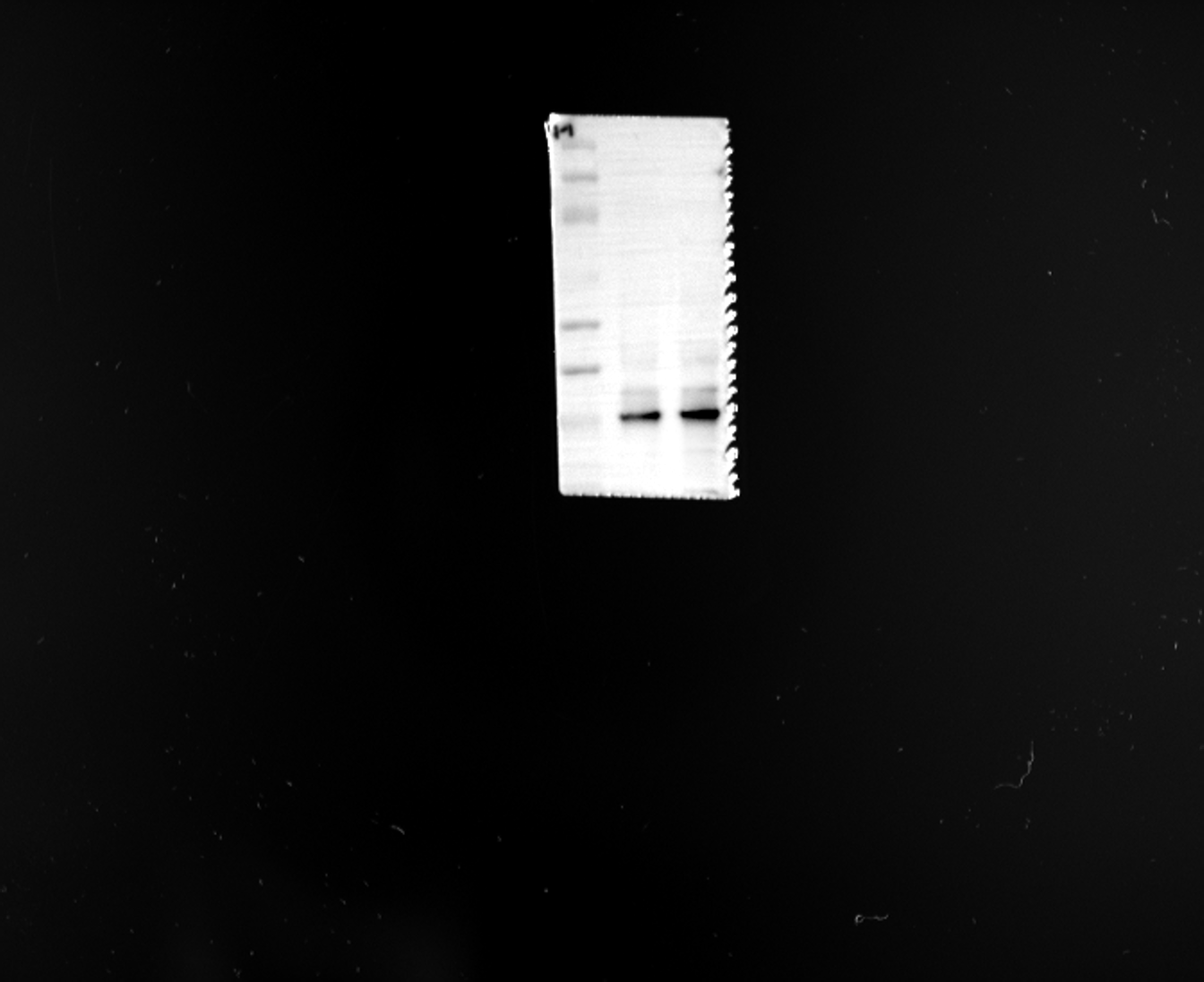

Supplement: Supplementary file 5 [file DataSheet5.zip › raw data traces-Figure 5D/MyD88.Tif]

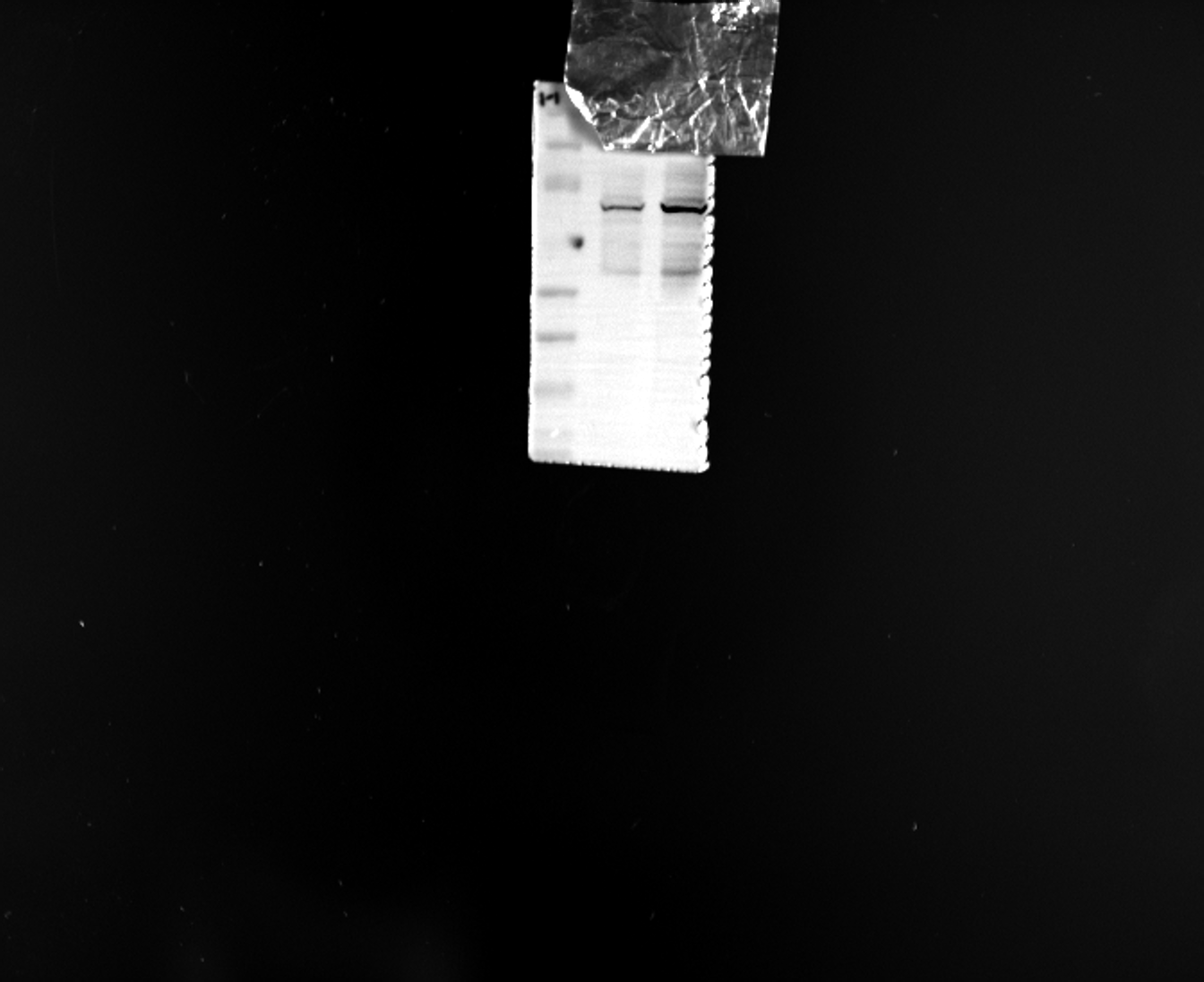

Supplement: Supplementary file 5 [file DataSheet5.zip › raw data traces-Figure 5D/TLR2.Tif]

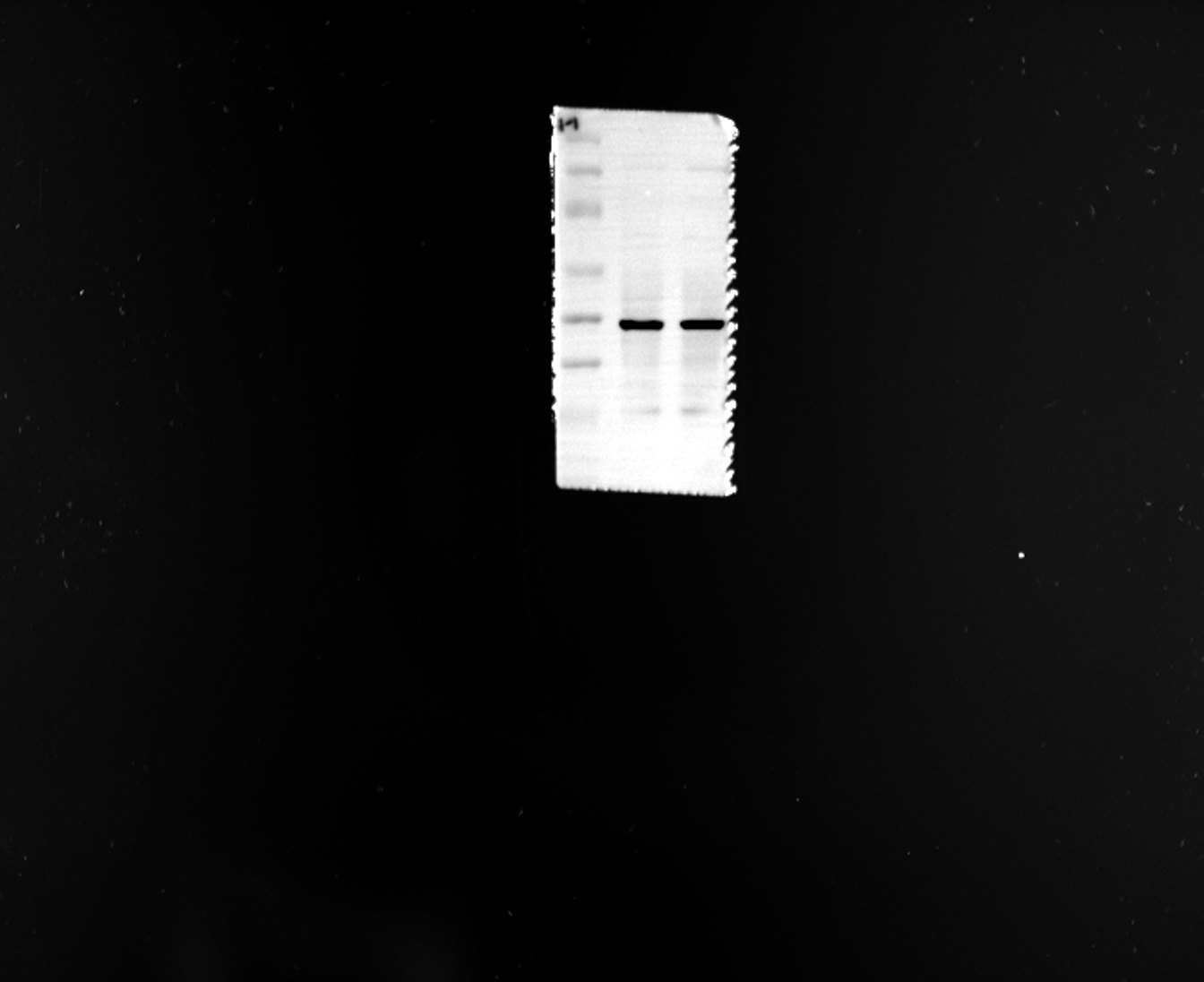

Supplement: Supplementary file 5 [file DataSheet5.zip › raw data traces-Figure 5D/tubulin.Tif]

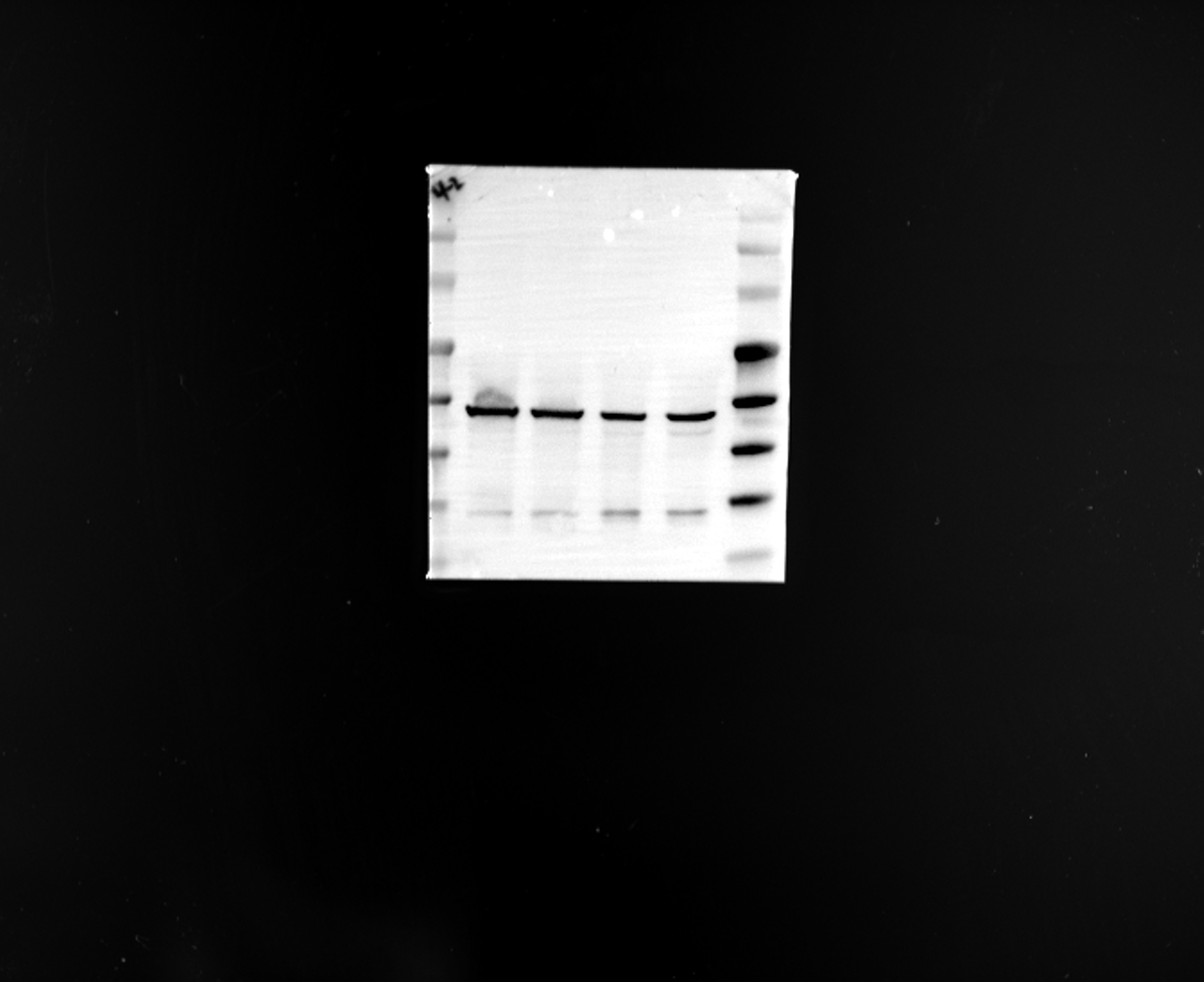

Supplement: Supplementary file 6 [file DataSheet6.zip › raw data traces-Figure 5E/MyD88/4-2tubu.Tif]

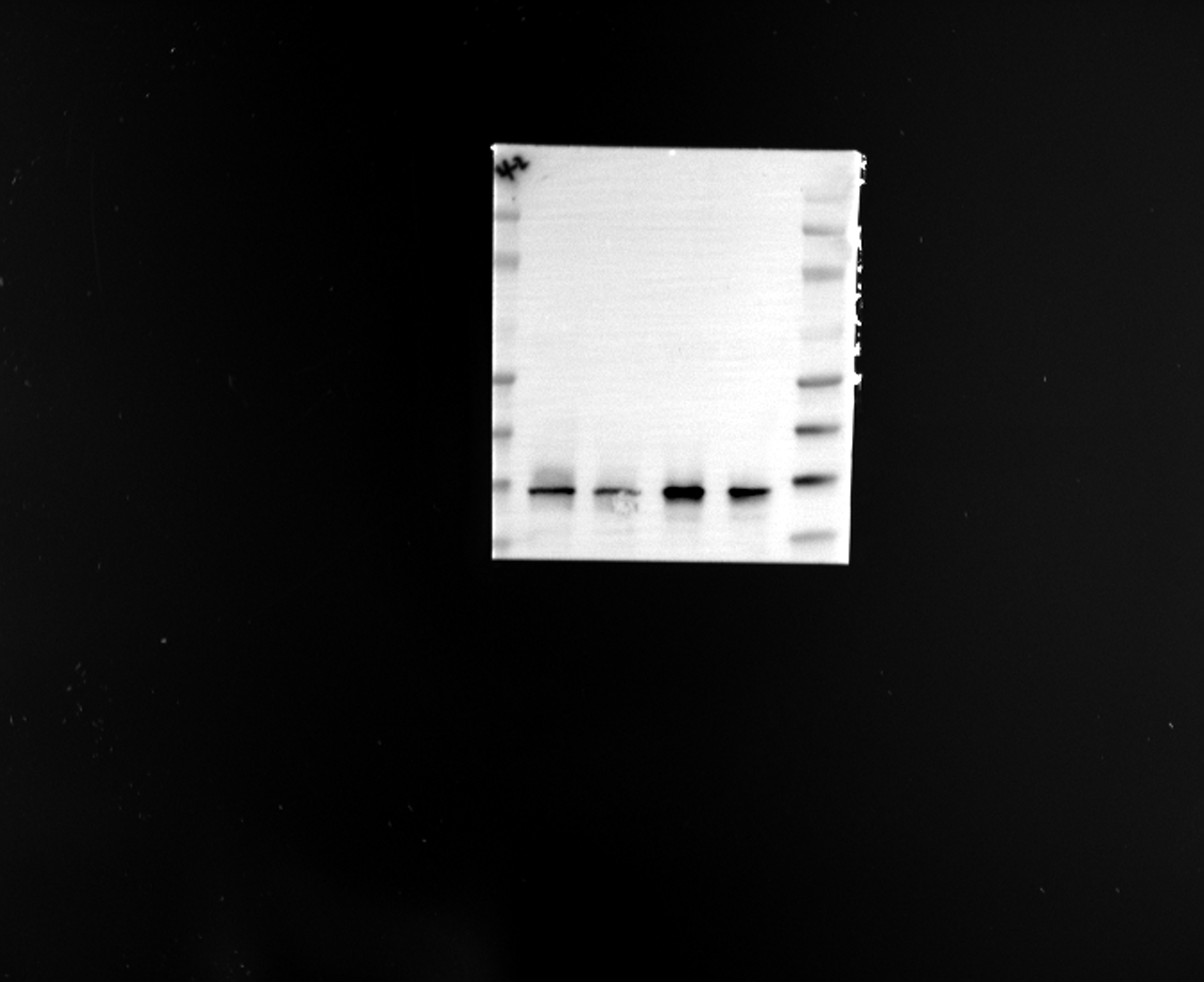

Supplement: Supplementary file 6 [file DataSheet6.zip › raw data traces-Figure 5E/MyD88/MyD88.Tif]

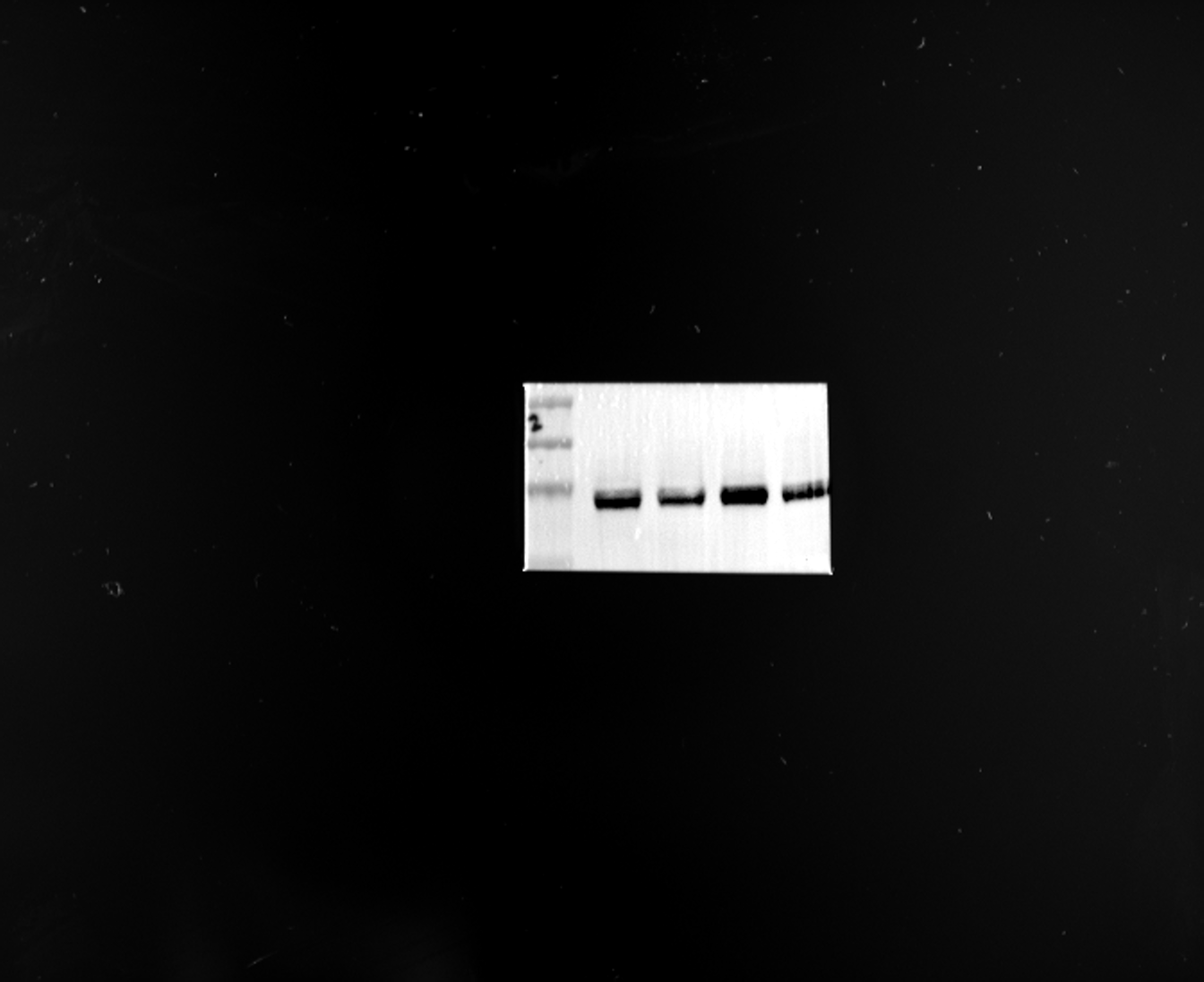

Supplement: Supplementary file 6 [file DataSheet6.zip › raw data traces-Figure 5E/TLR2/TLR2.Tif]

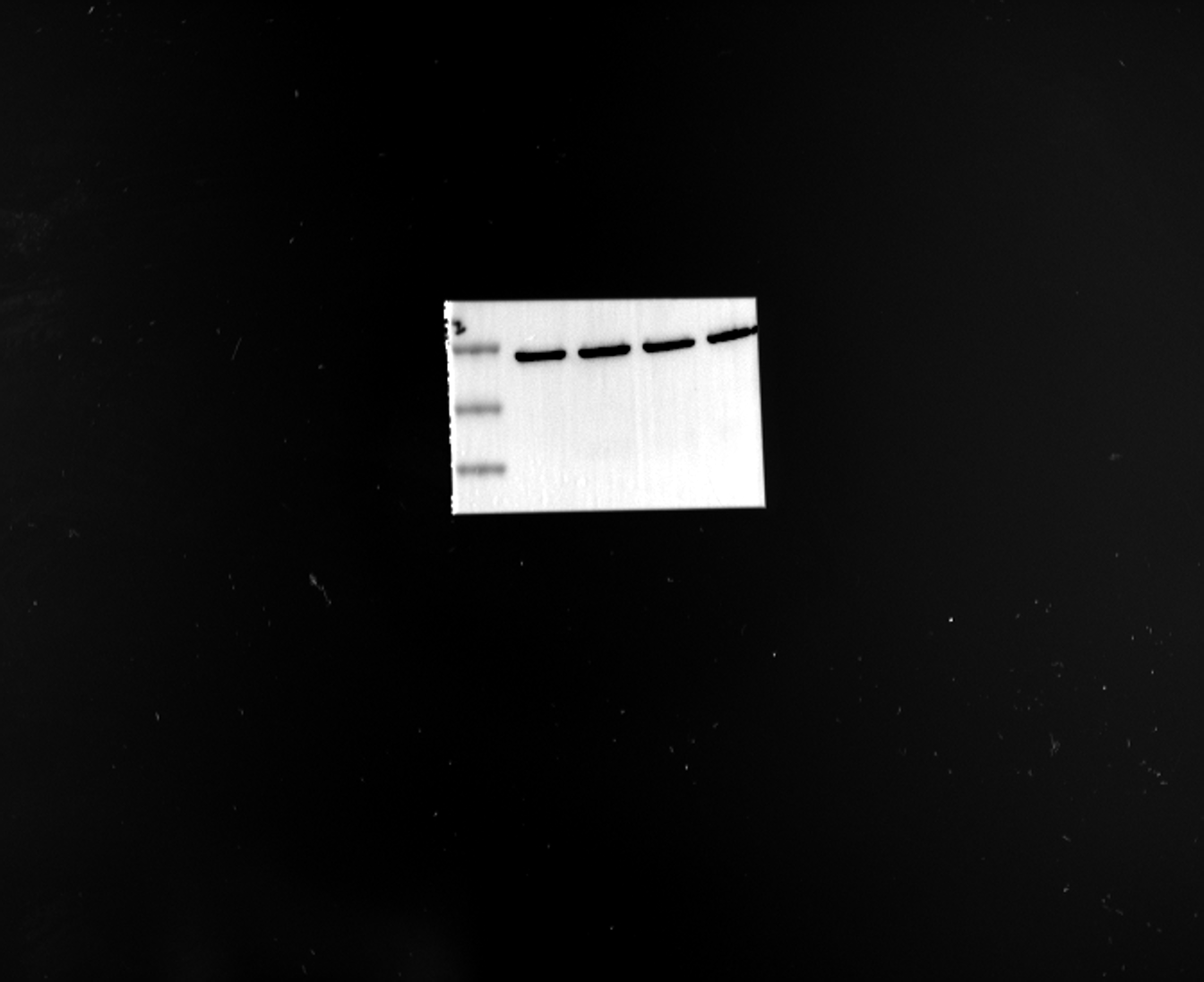

Supplement: Supplementary file 6 [file DataSheet6.zip › raw data traces-Figure 5E/TLR2/tubulin.Tif]

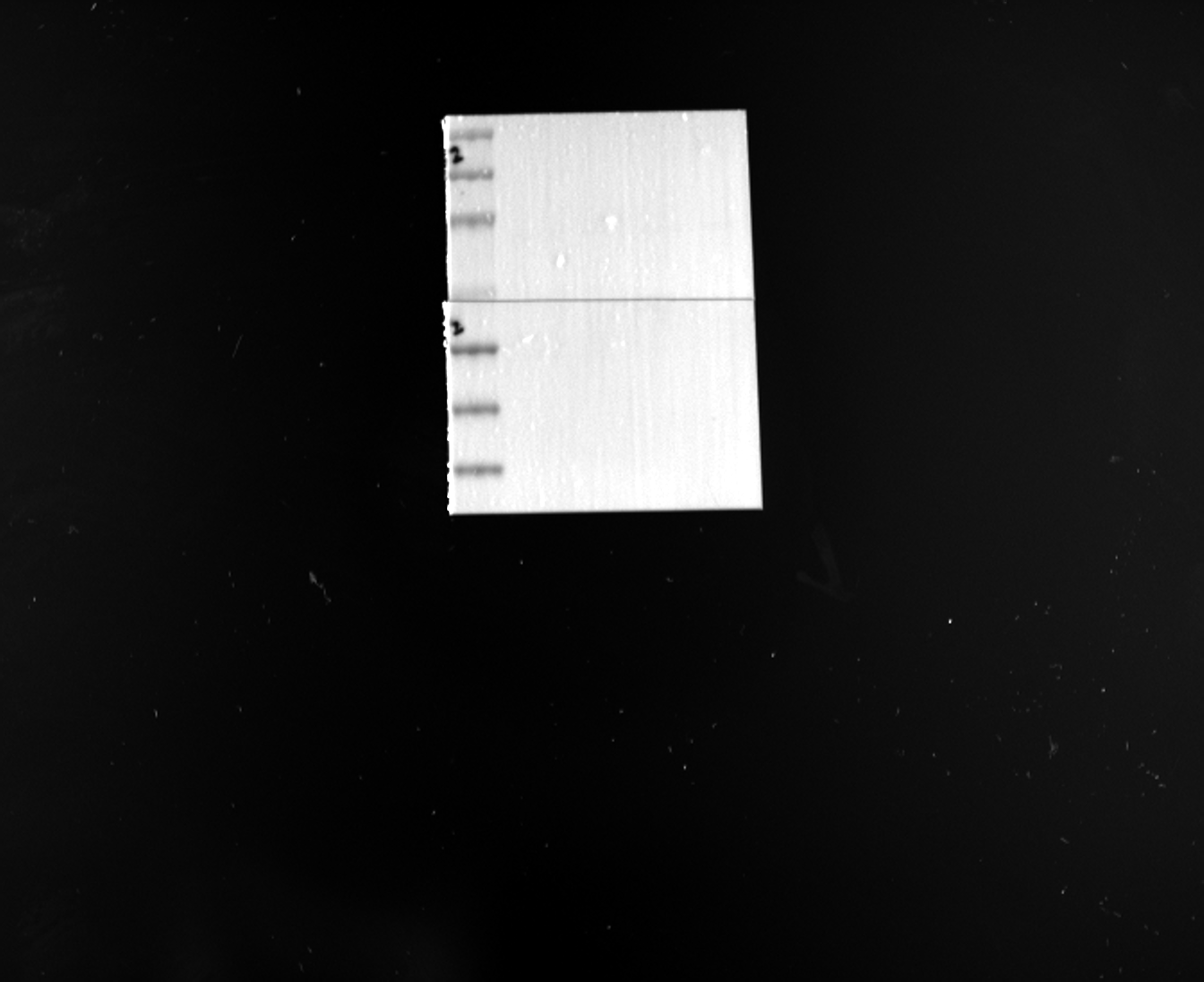

Supplement: Supplementary file 6 [file DataSheet6.zip › raw data traces-Figure 5E/TLR2/Uncropped images of blots.Tif]

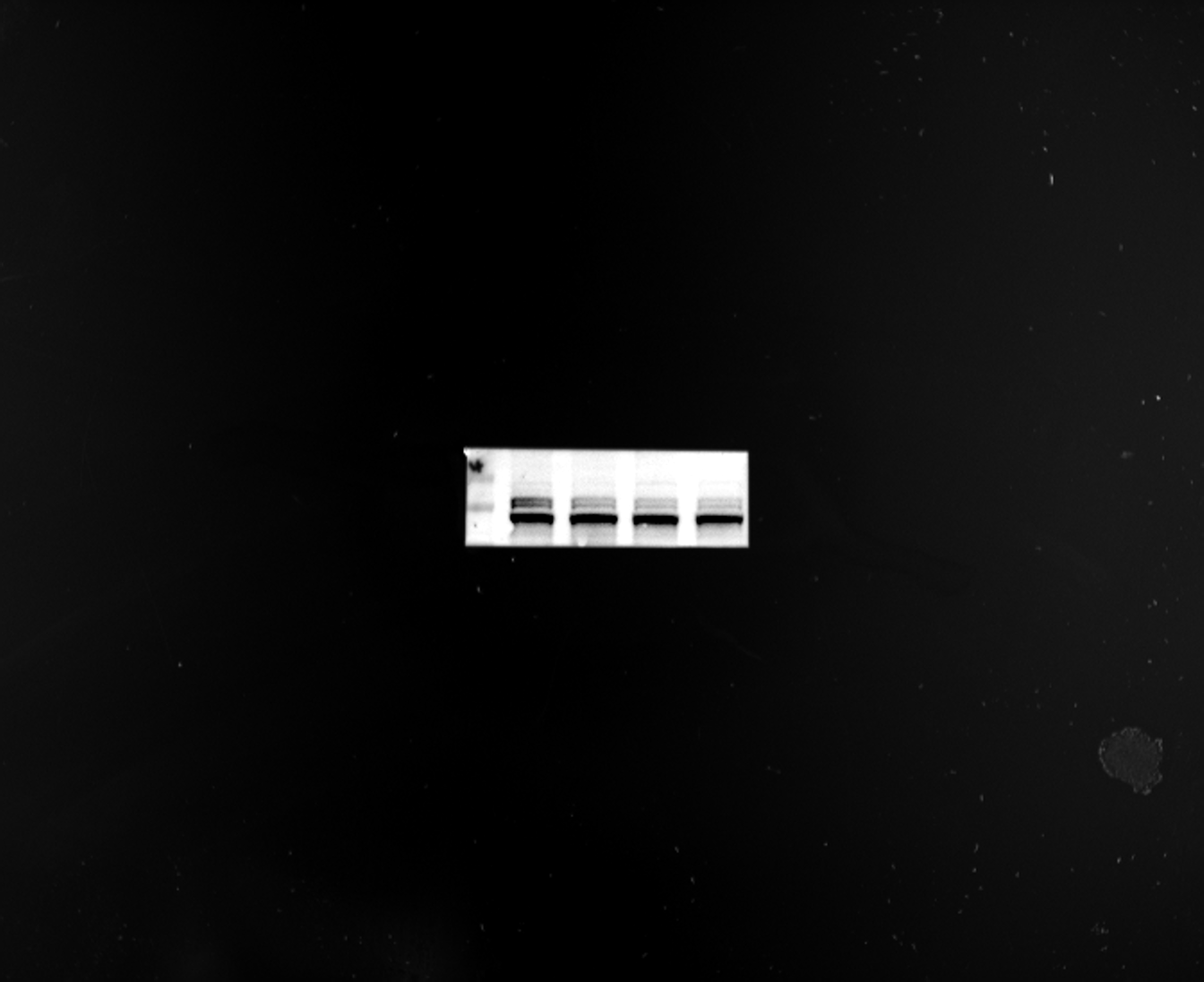

Supplement: Supplementary file 7 [file DataSheet7.zip › raw data traces-Figure 6A/JAK2/JAK2.Tif]

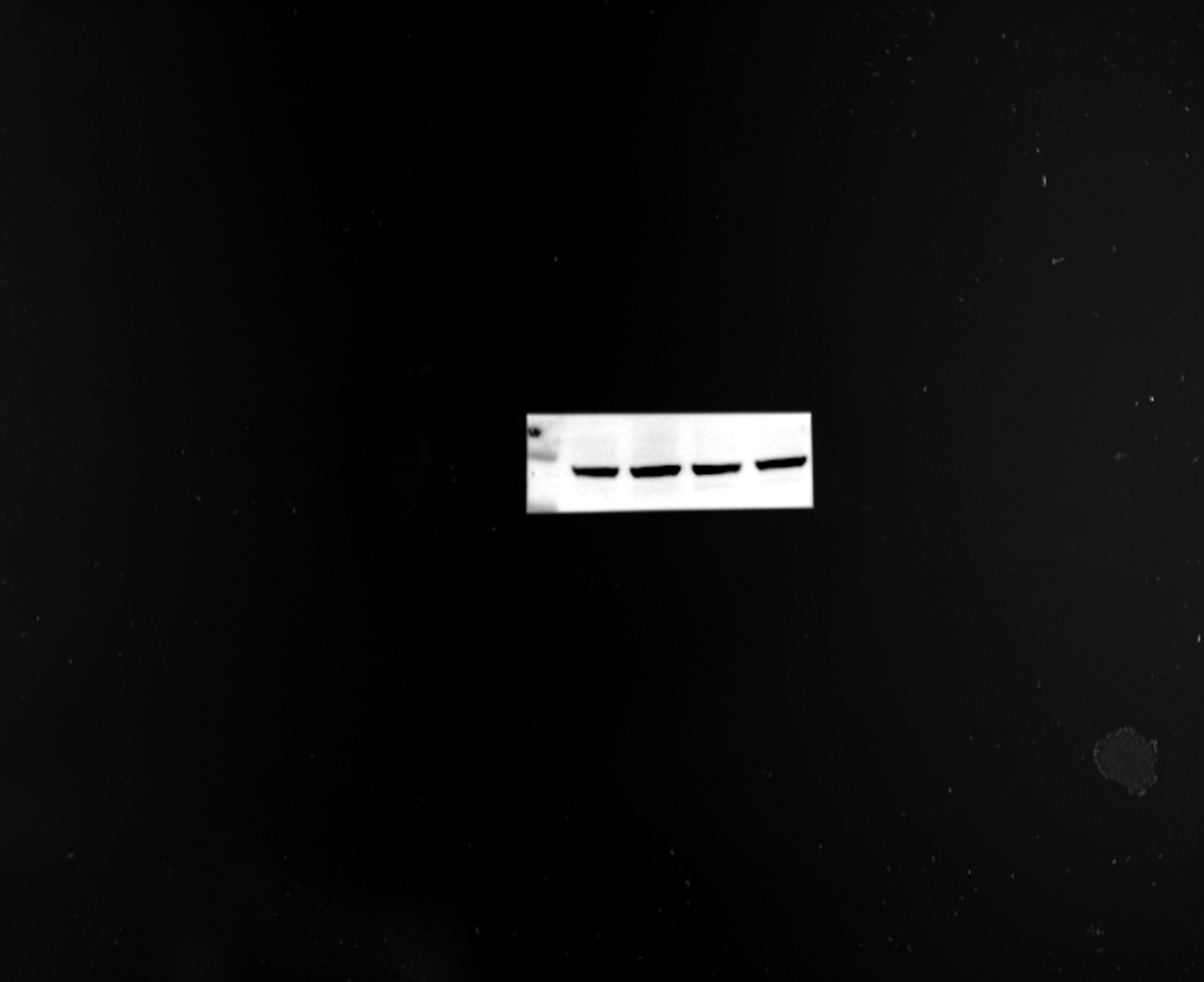

Supplement: Supplementary file 7 [file DataSheet7.zip › raw data traces-Figure 6A/JAK2/tubulin.Tif]

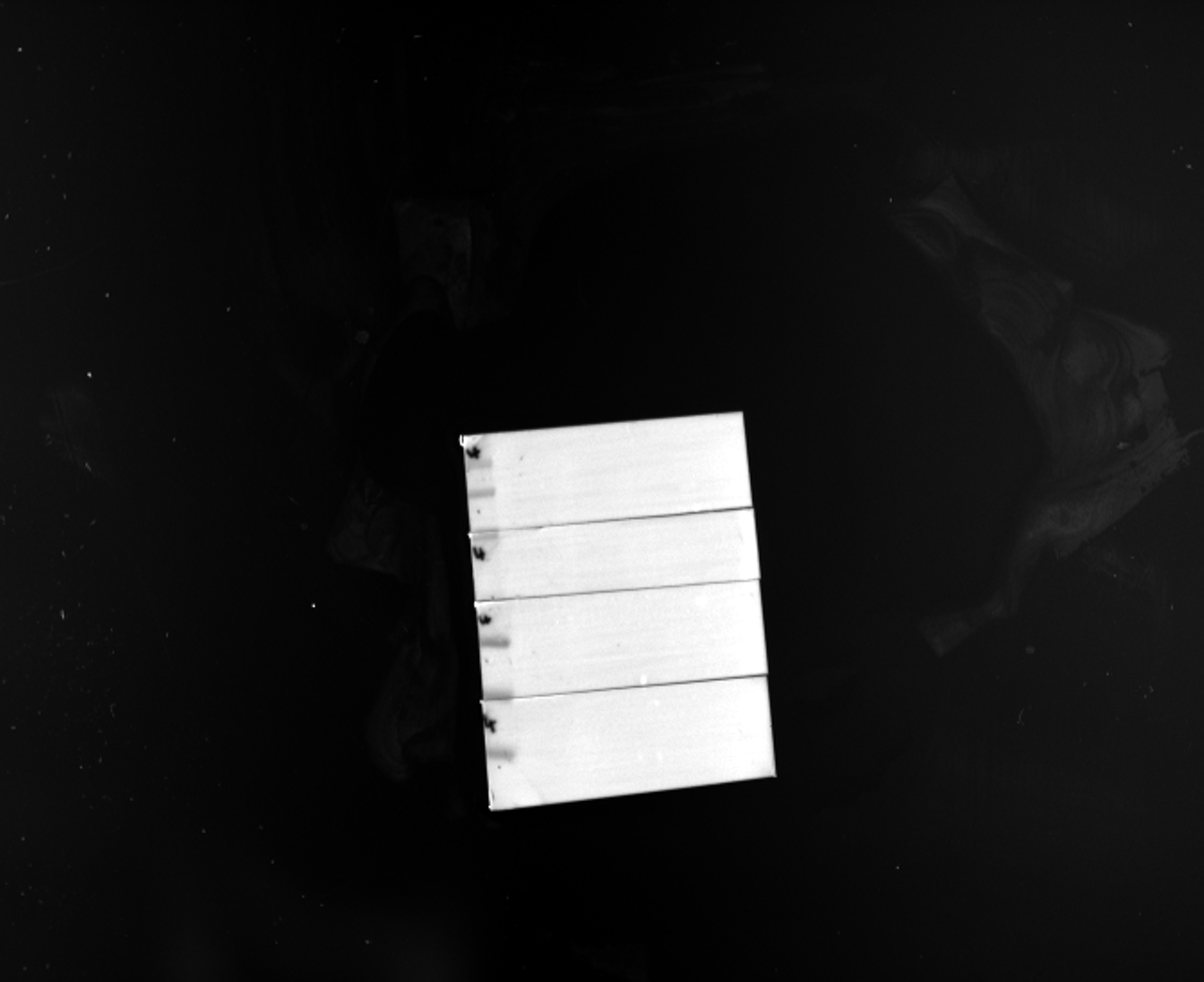

Supplement: Supplementary file 7 [file DataSheet7.zip › raw data traces-Figure 6A/JAK2/Uncropped images of blots.Tif]

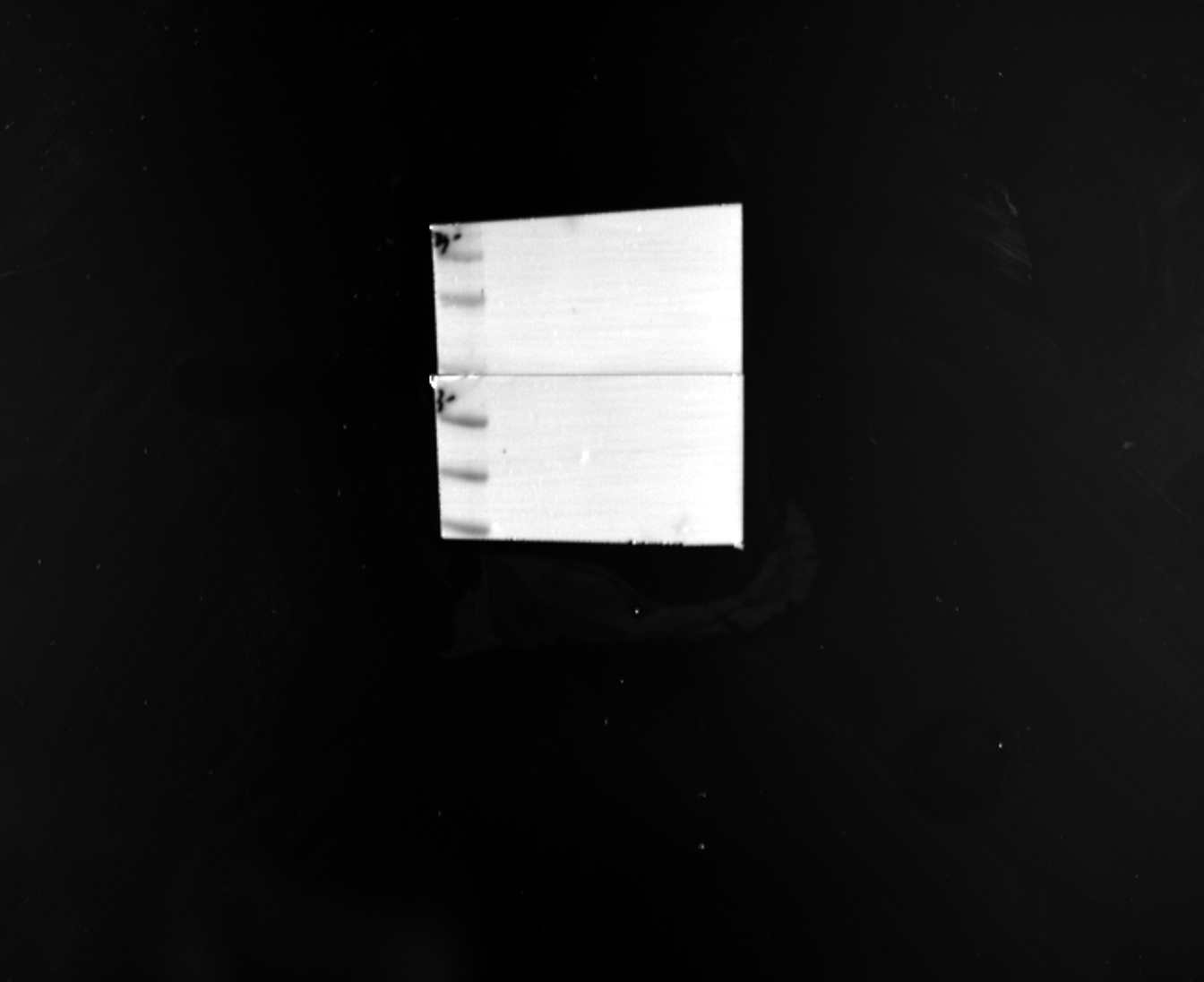

Supplement: Supplementary file 7 [file DataSheet7.zip › raw data traces-Figure 6A/p-JAK2/3--全图.Tif]

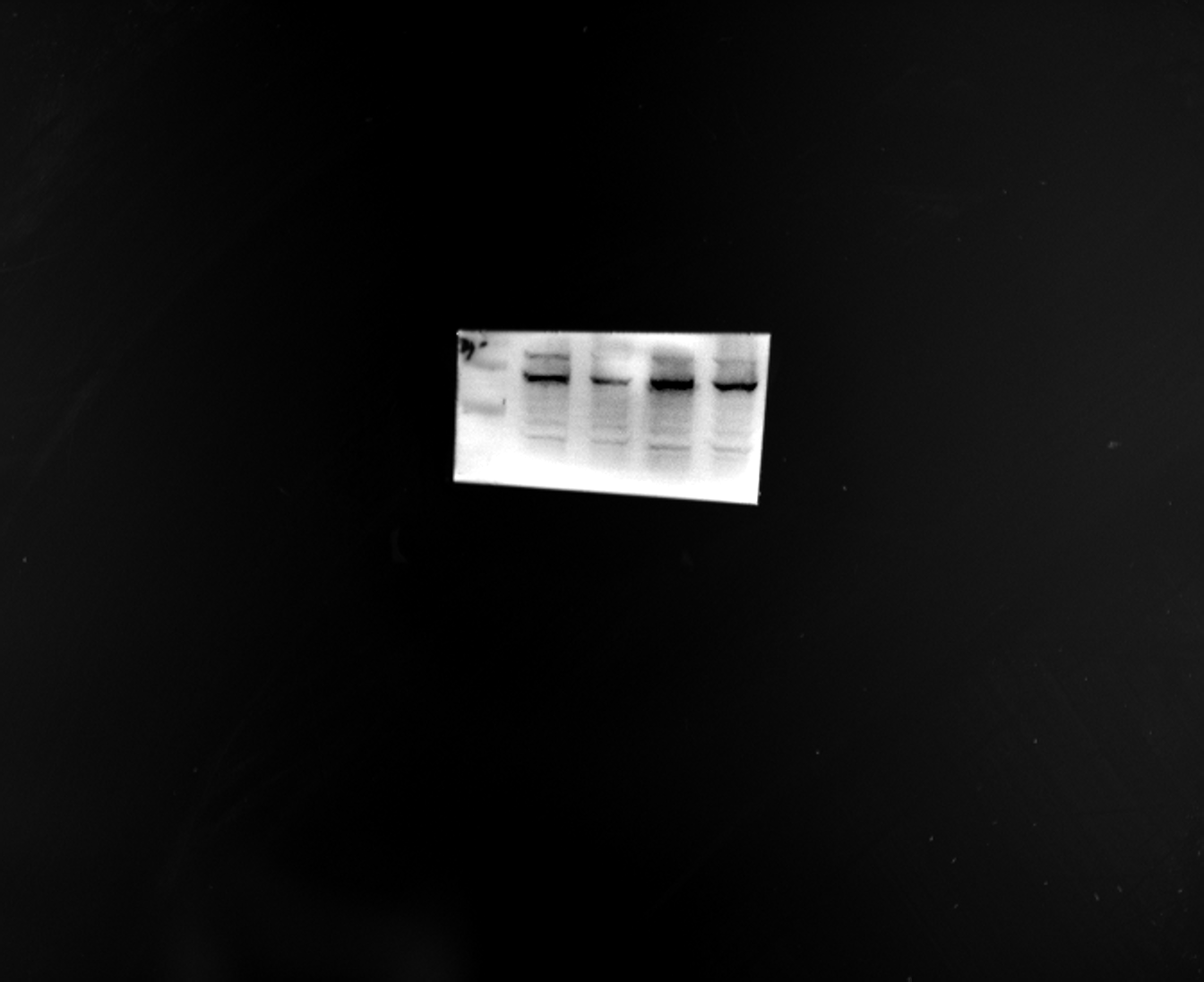

Supplement: Supplementary file 7 [file DataSheet7.zip › raw data traces-Figure 6A/p-JAK2/p-JAK2.Tif]

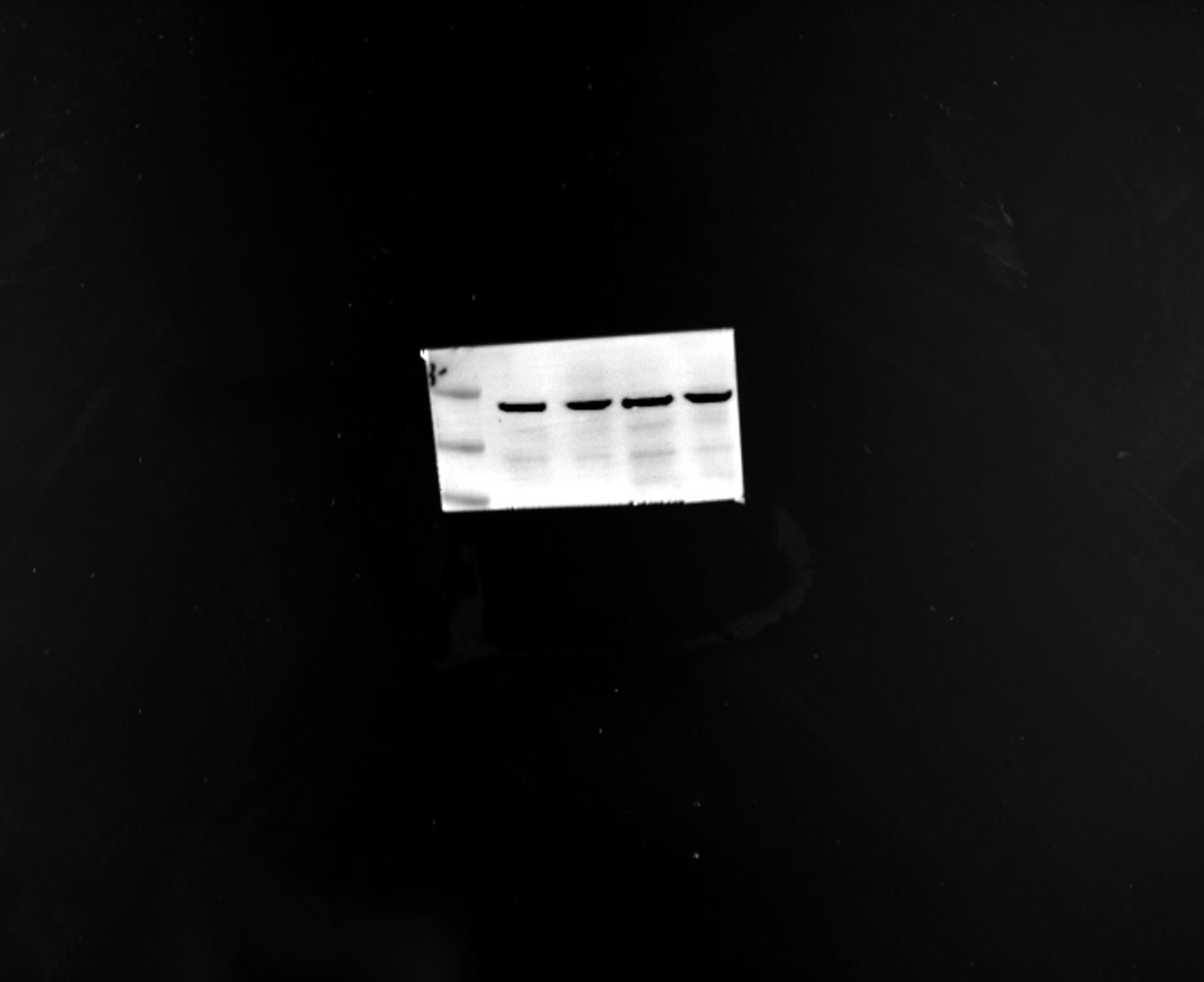

Supplement: Supplementary file 7 [file DataSheet7.zip › raw data traces-Figure 6A/p-JAK2/tubulin.Tif]

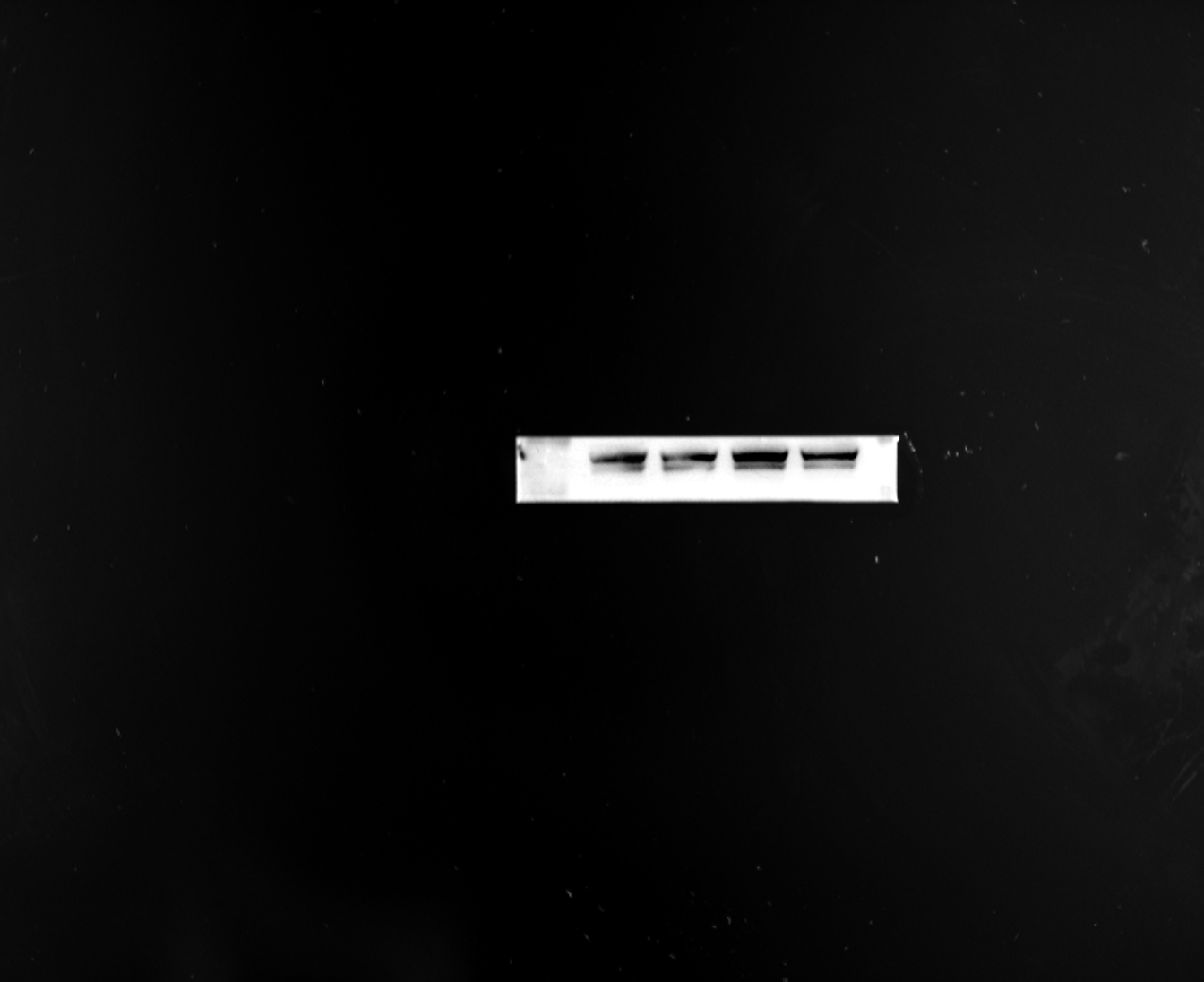

Supplement: Supplementary file 7 [file DataSheet7.zip › raw data traces-Figure 6A/p-STAT3/1-STAT33.Tif]

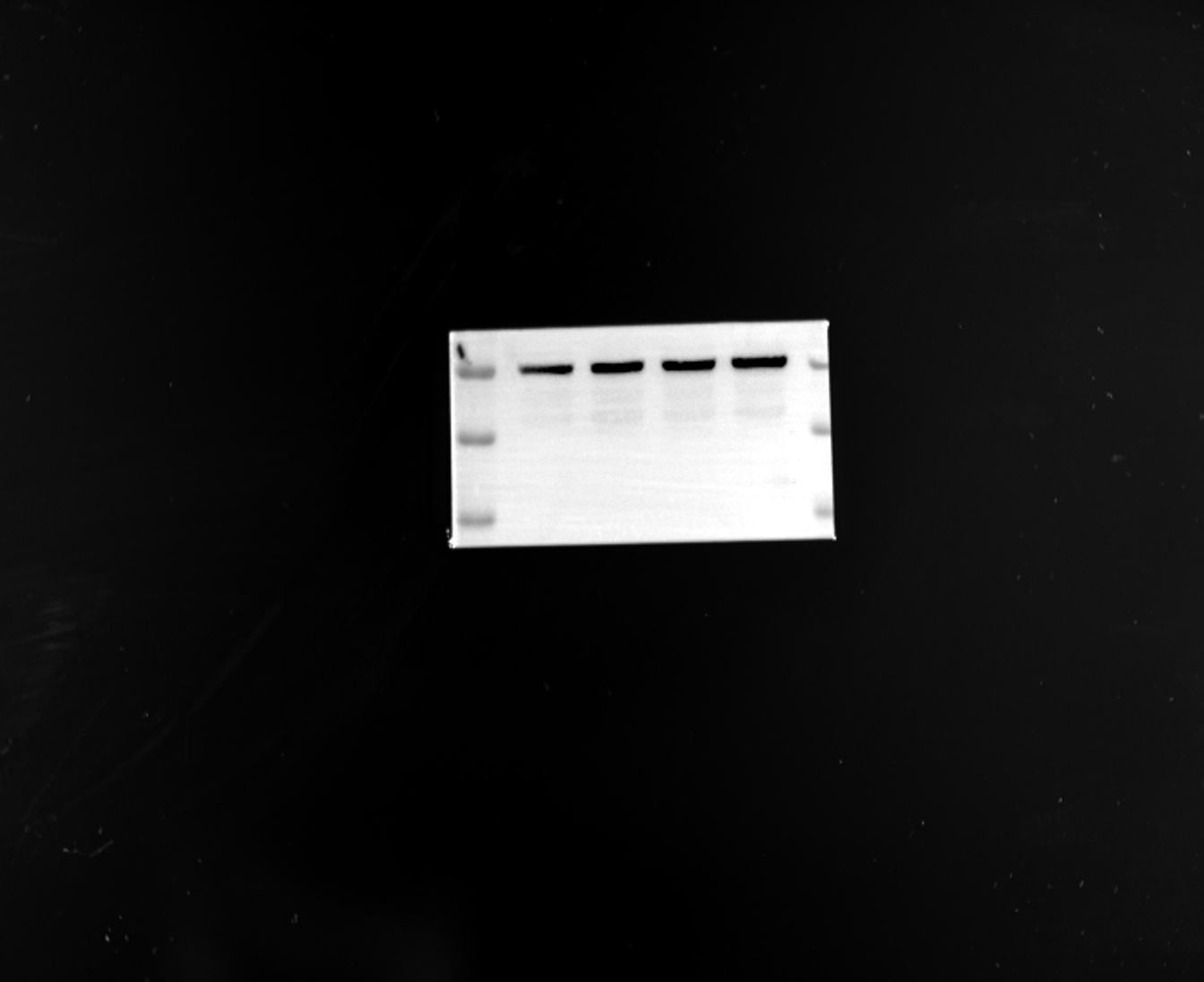

Supplement: Supplementary file 7 [file DataSheet7.zip › raw data traces-Figure 6A/p-STAT3/tubulin.Tif]

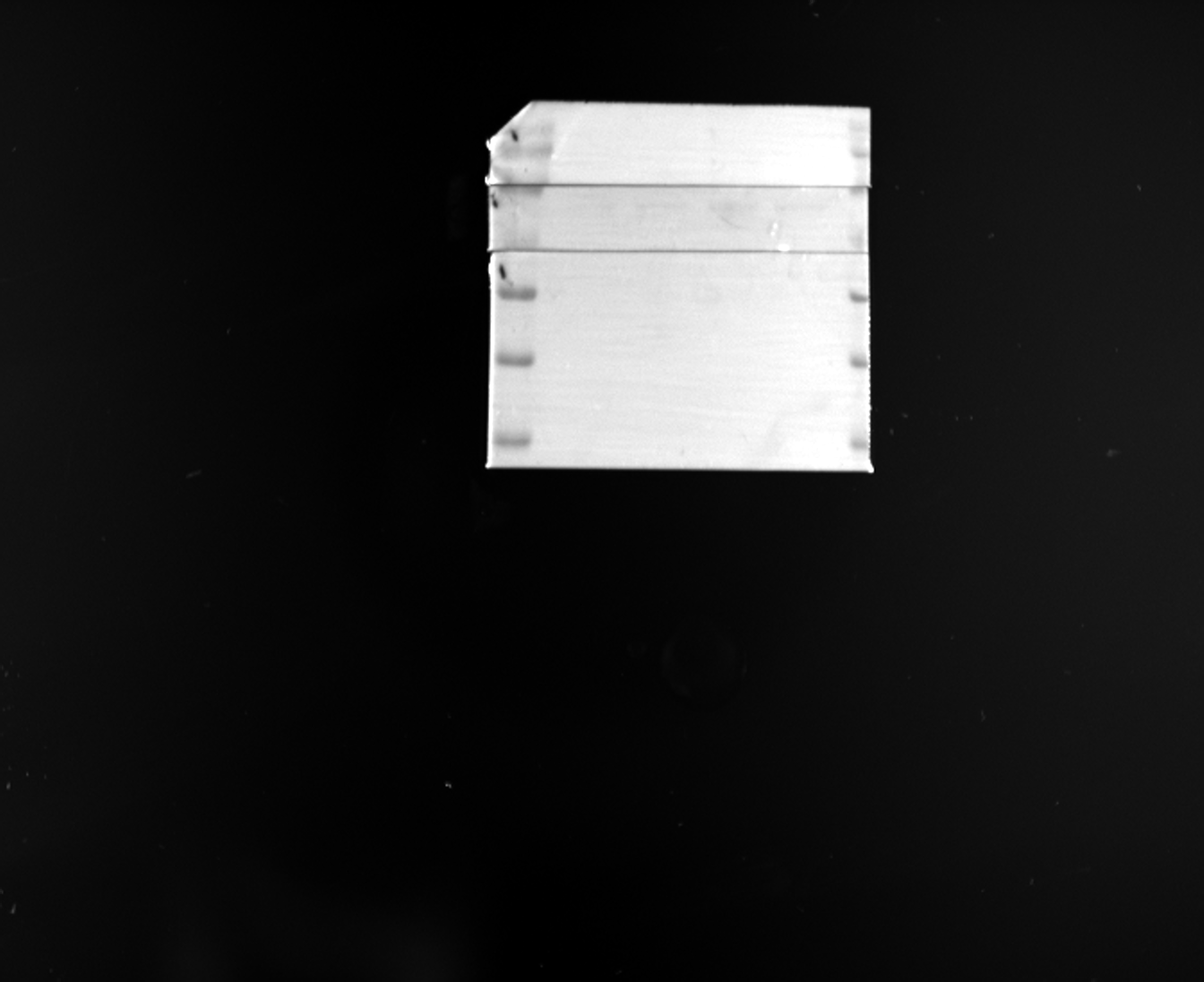

Supplement: Supplementary file 7 [file DataSheet7.zip › raw data traces-Figure 6A/p-STAT3/Uncropped images of blots.Tif]

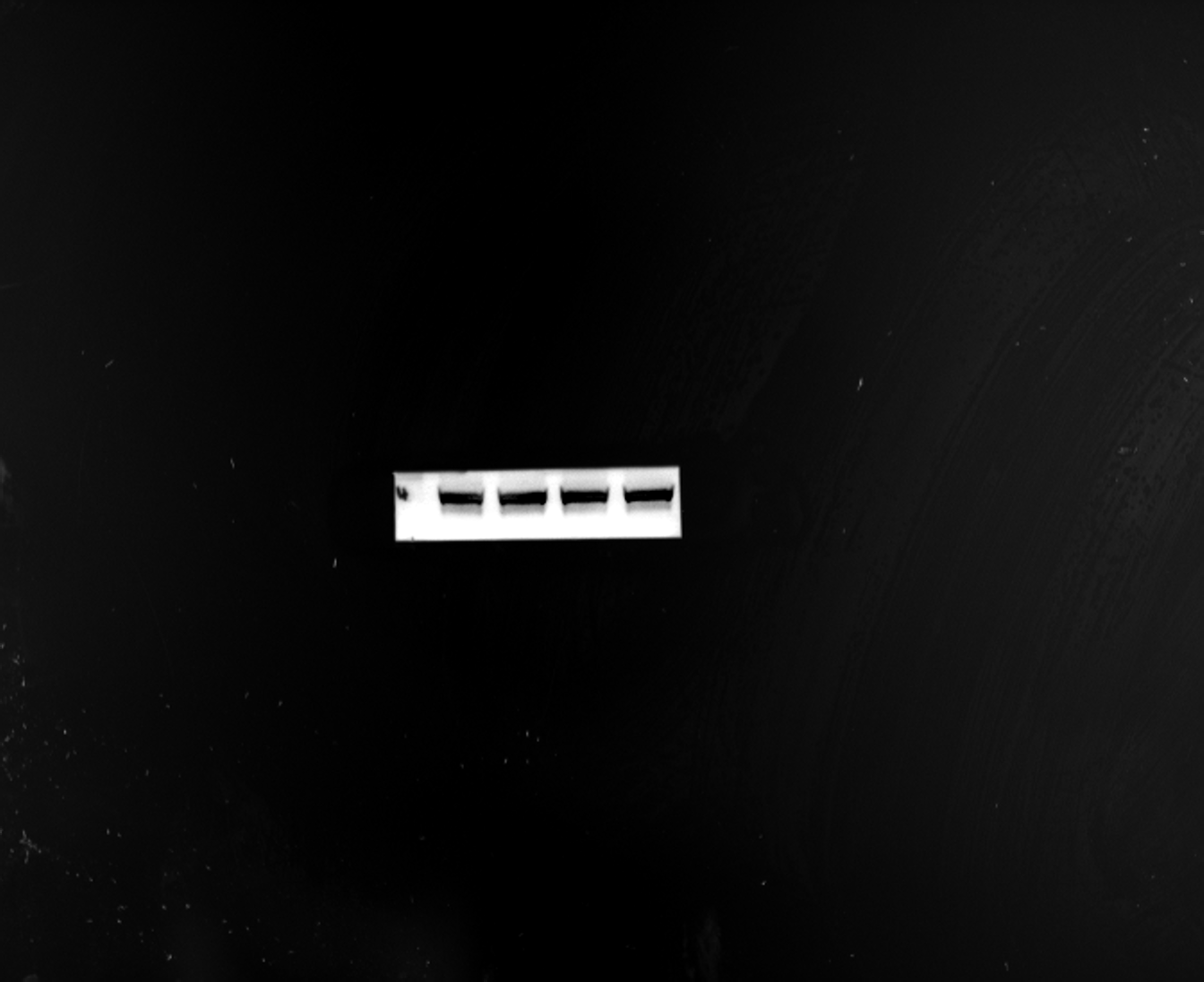

Supplement: Supplementary file 7 [file DataSheet7.zip › raw data traces-Figure 6A/STAT3/STAT3.Tif]

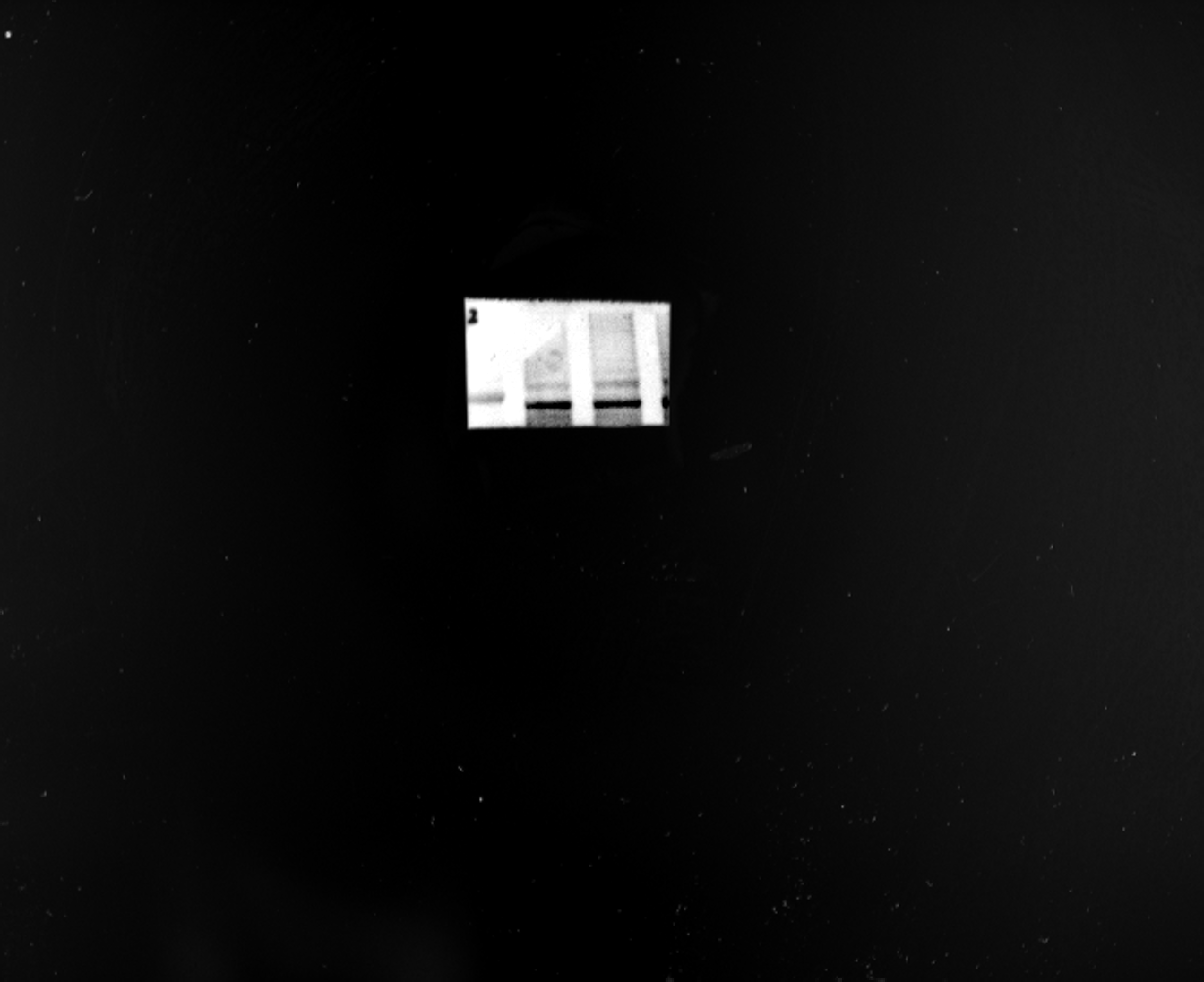

Supplement: Supplementary file 8 [file DataSheet8.zip › raw data traces-Figure 6D/JAK2/2-TJAK2.Tif]

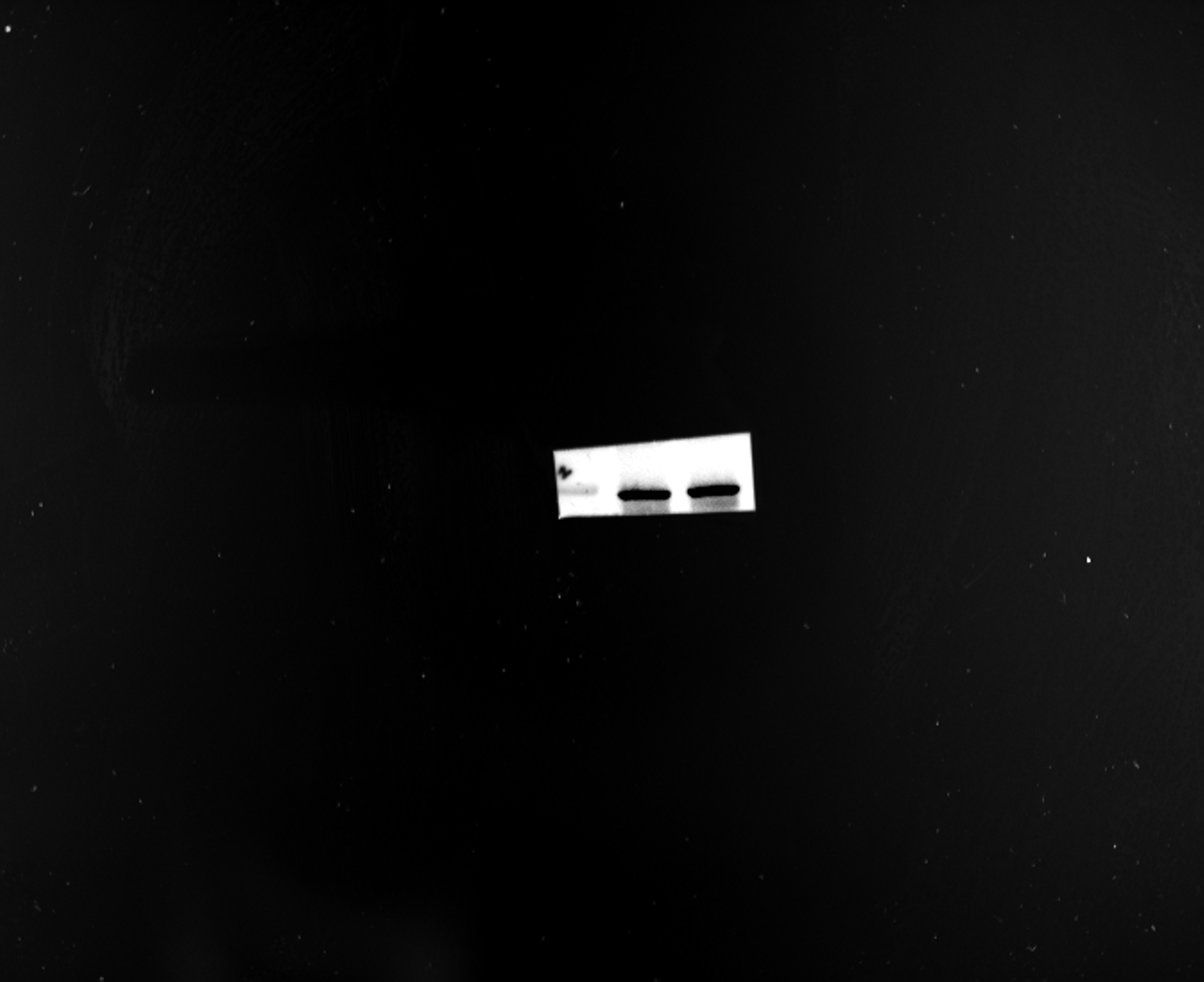

Supplement: Supplementary file 8 [file DataSheet8.zip › raw data traces-Figure 6D/JAK2/tubulin.Tif]

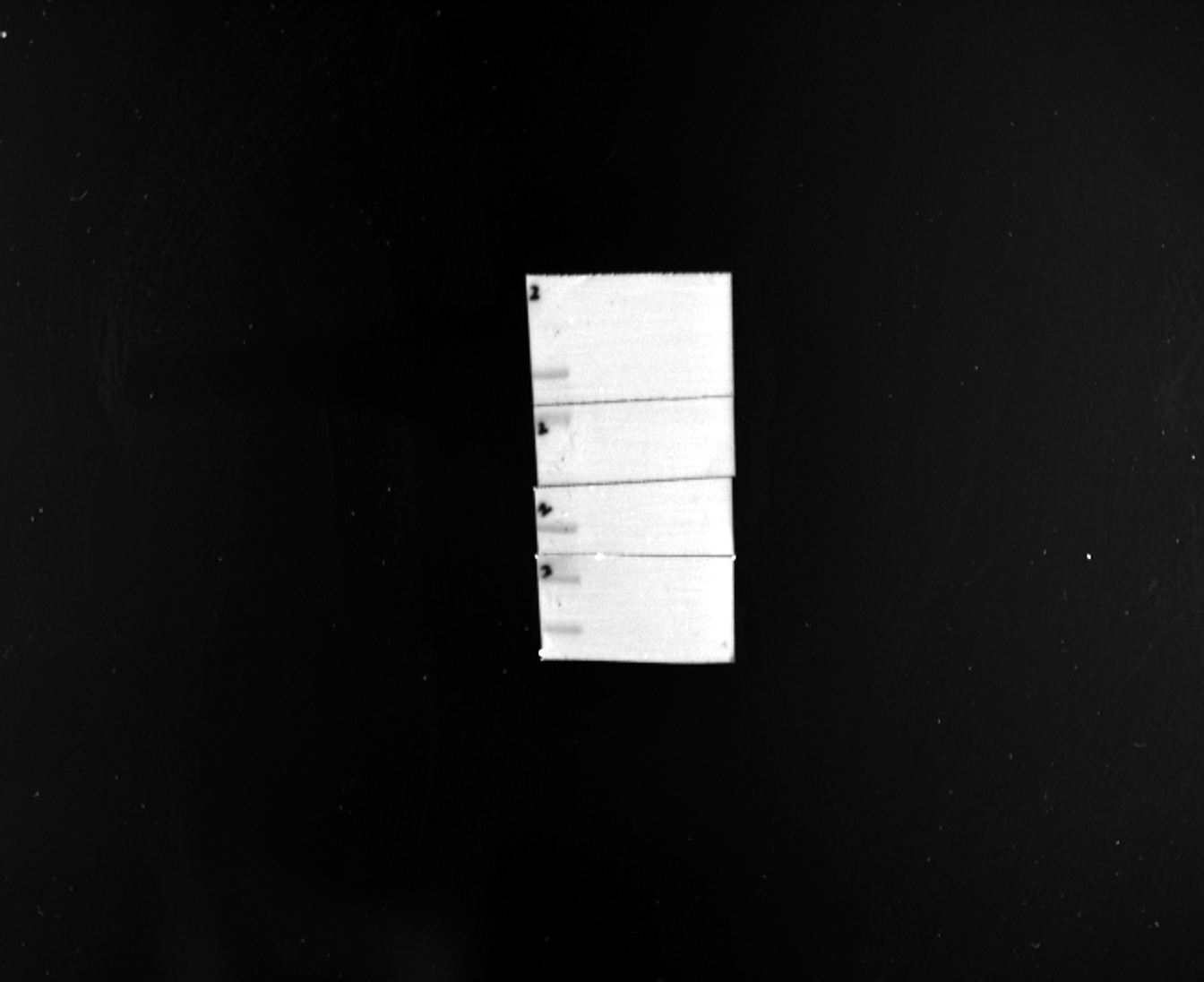

Supplement: Supplementary file 8 [file DataSheet8.zip › raw data traces-Figure 6D/JAK2/Uncropped images of blots.Tif]

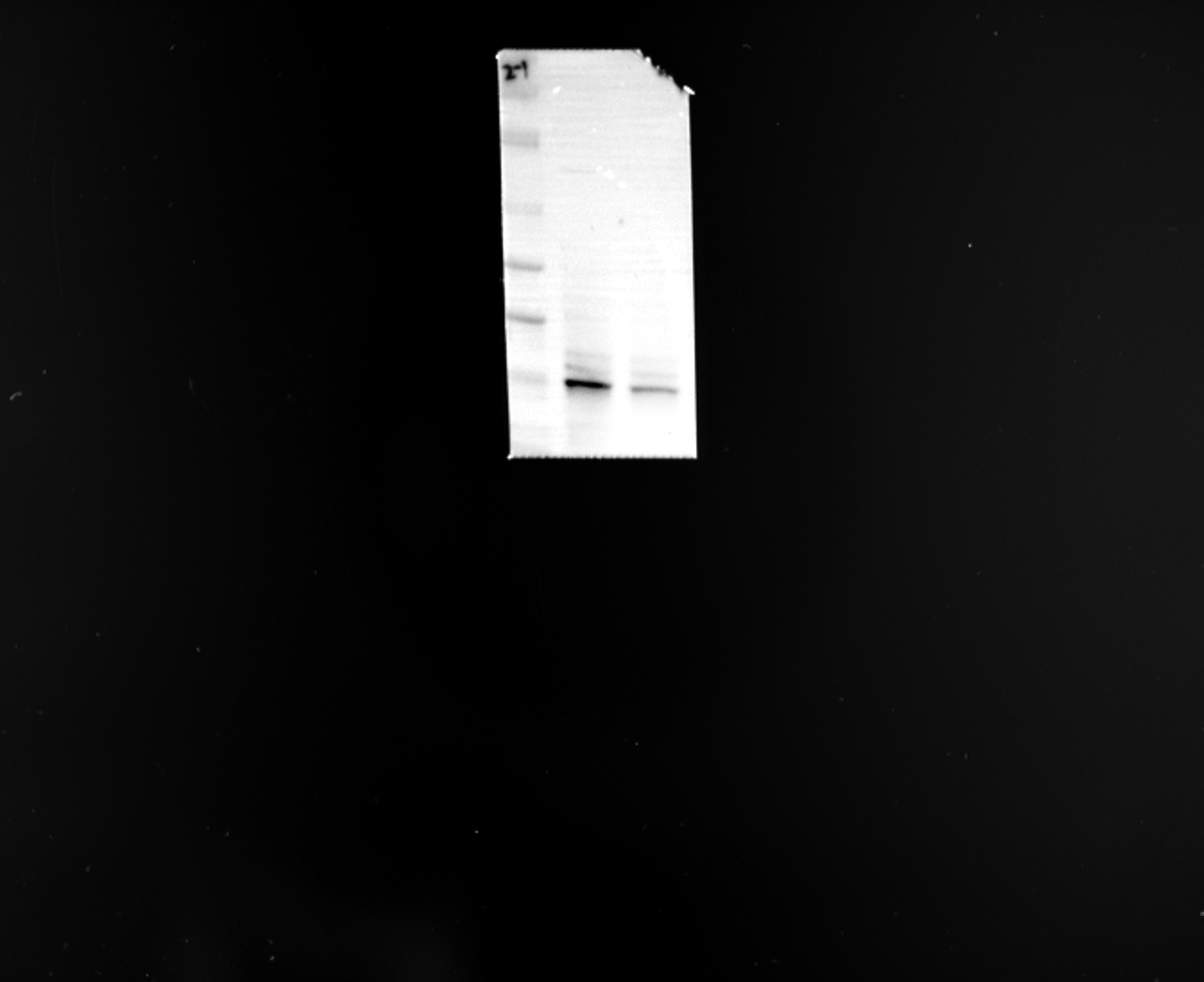

Supplement: Supplementary file 8 [file DataSheet8.zip › raw data traces-Figure 6D/MyD88/MyD88.Tif]

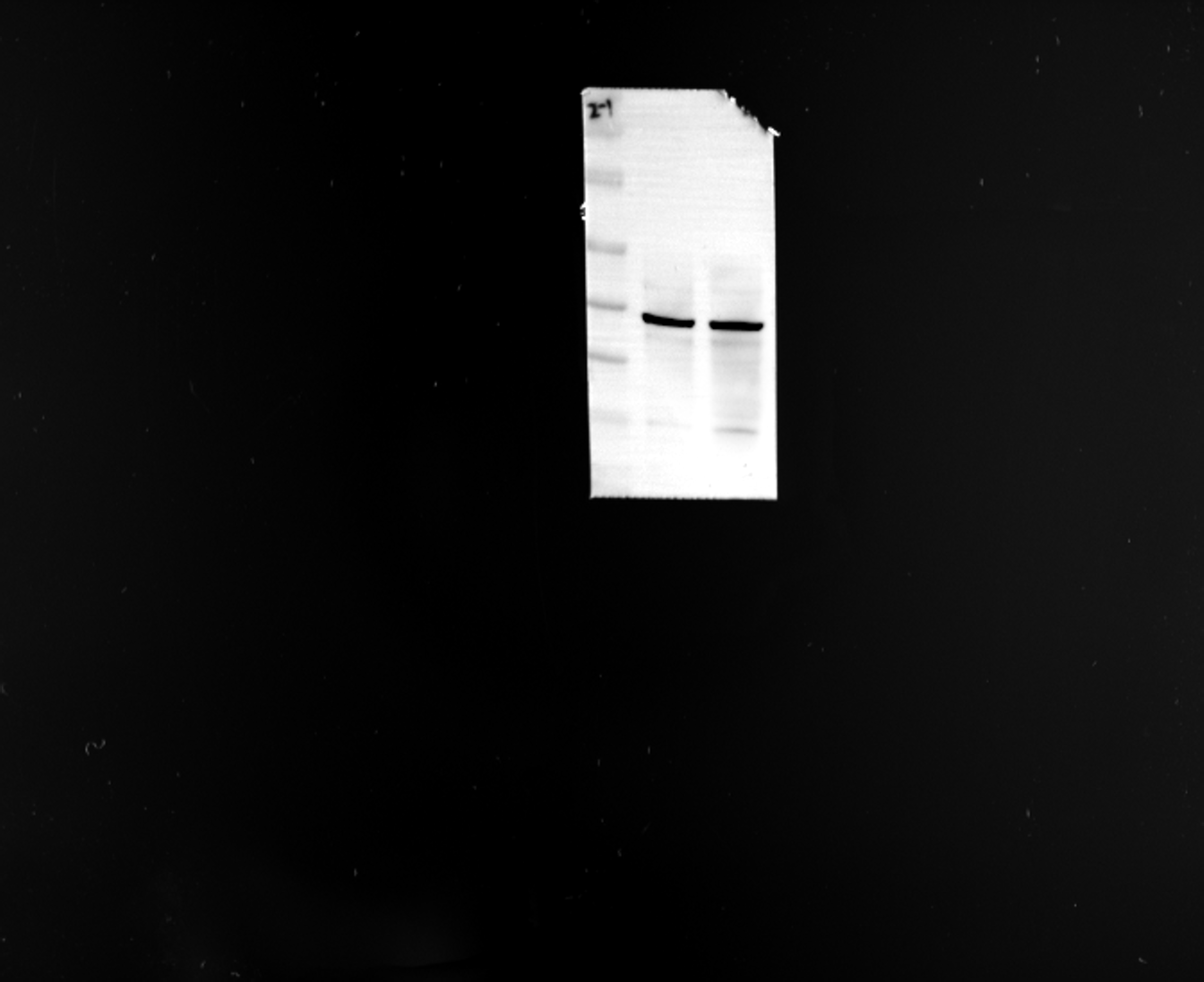

Supplement: Supplementary file 8 [file DataSheet8.zip › raw data traces-Figure 6D/MyD88/tubulin.Tif]

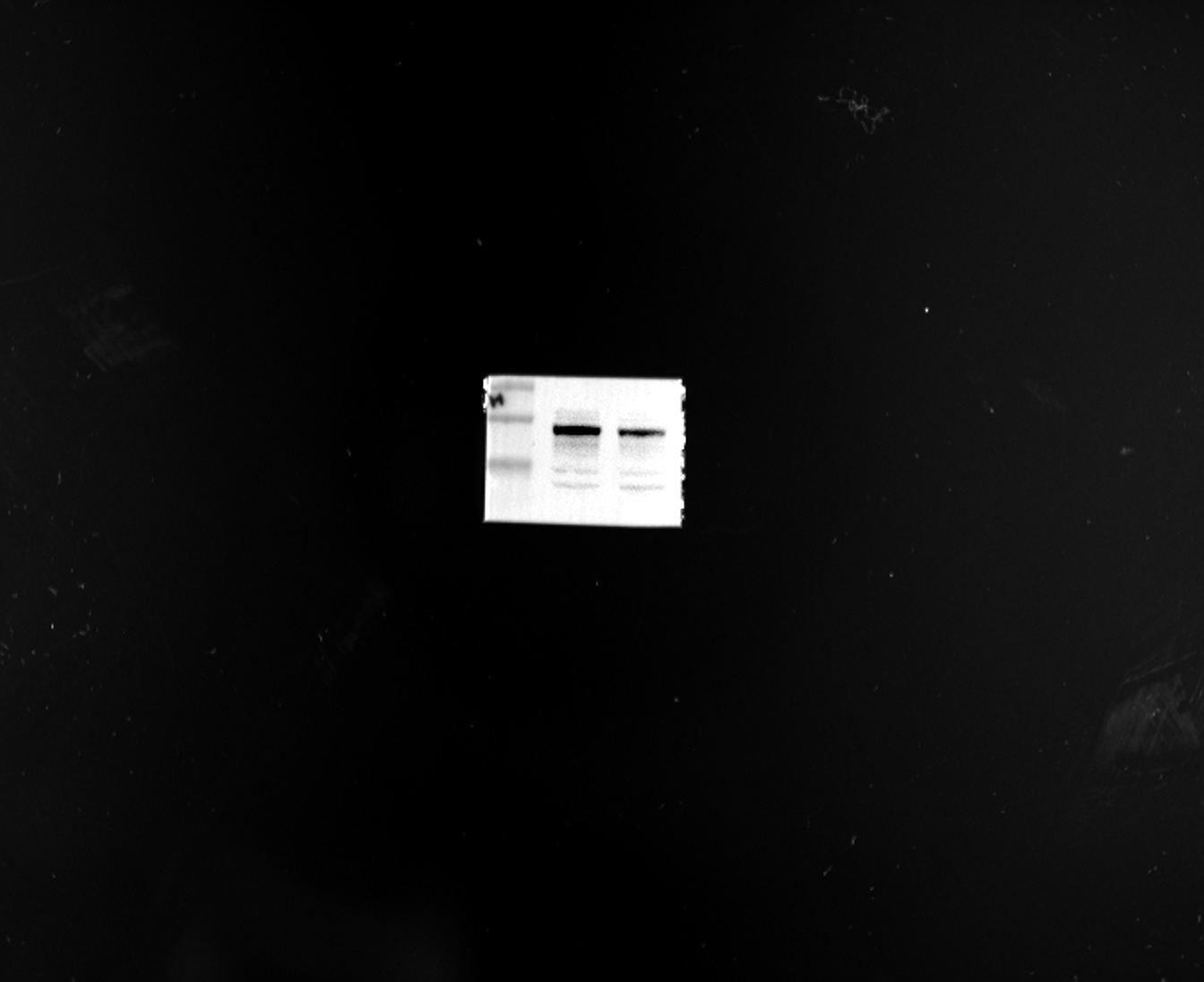

Supplement: Supplementary file 8 [file DataSheet8.zip › raw data traces-Figure 6D/p-JAK2/p-JAK2.Tif]

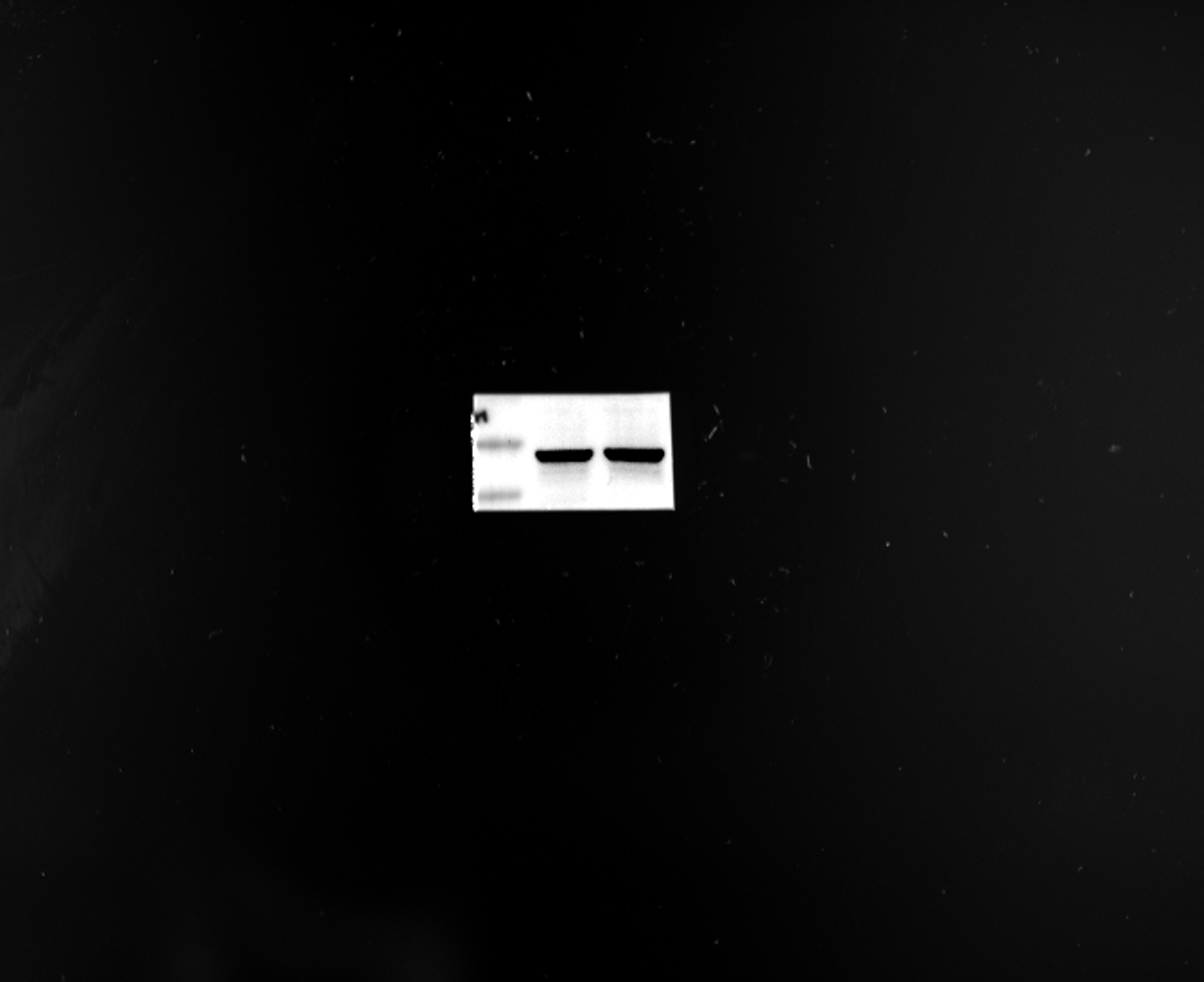

Supplement: Supplementary file 8 [file DataSheet8.zip › raw data traces-Figure 6D/p-JAK2/tubulin.Tif]

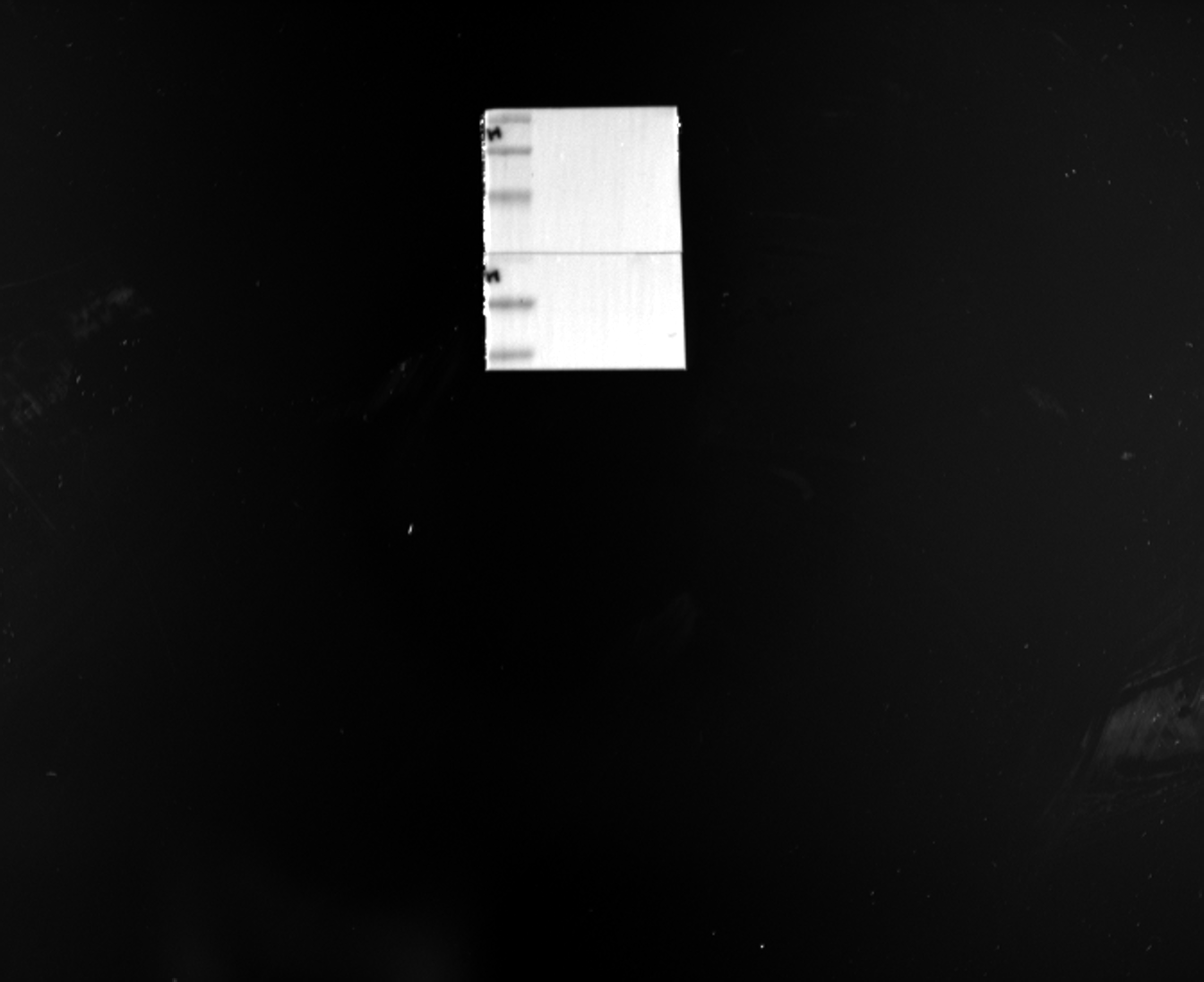

Supplement: Supplementary file 8 [file DataSheet8.zip › raw data traces-Figure 6D/p-JAK2/Uncropped images of blots.Tif]

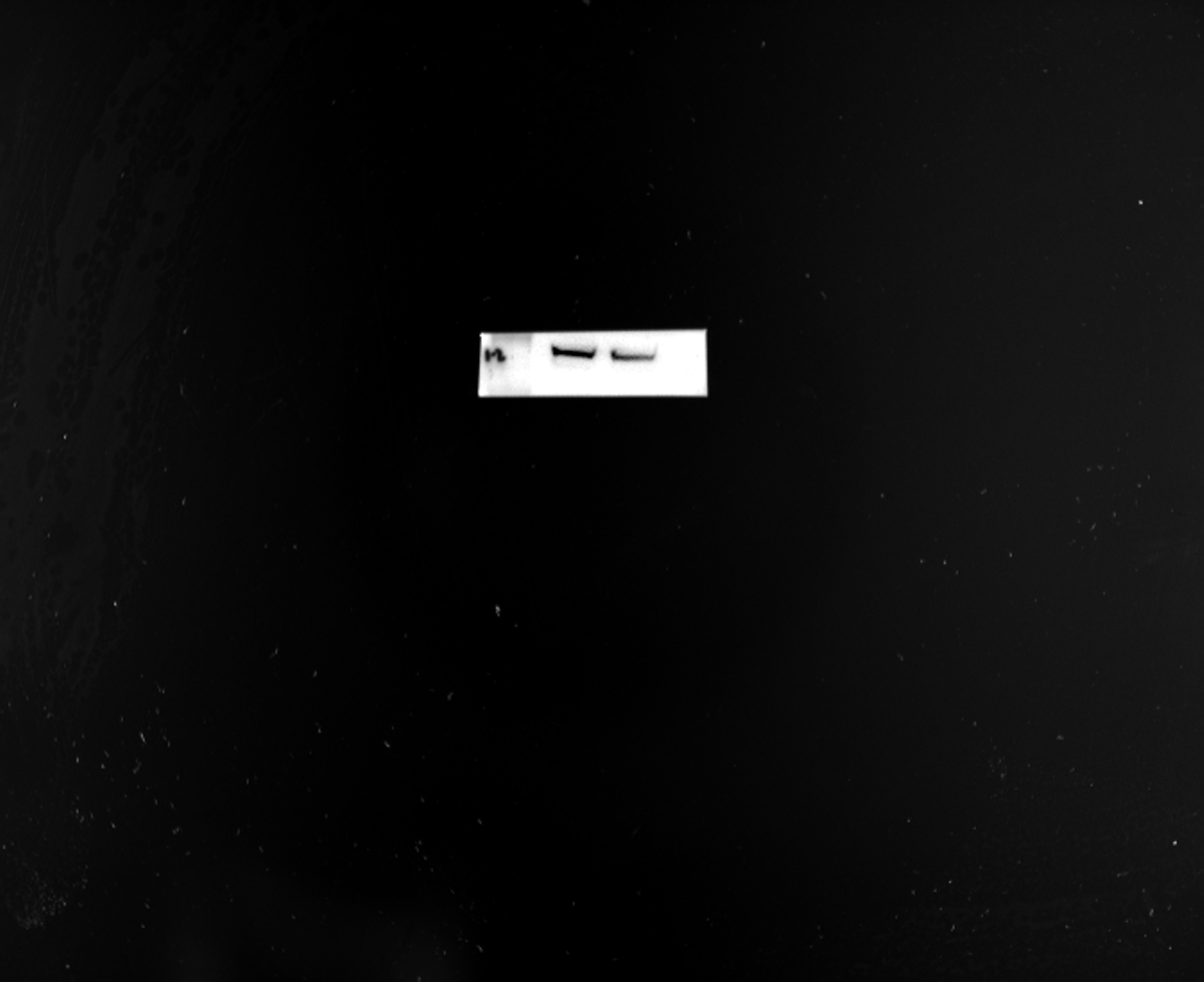

Supplement: Supplementary file 8 [file DataSheet8.zip › raw data traces-Figure 6D/p-STAT3/p-STAT3.Tif]

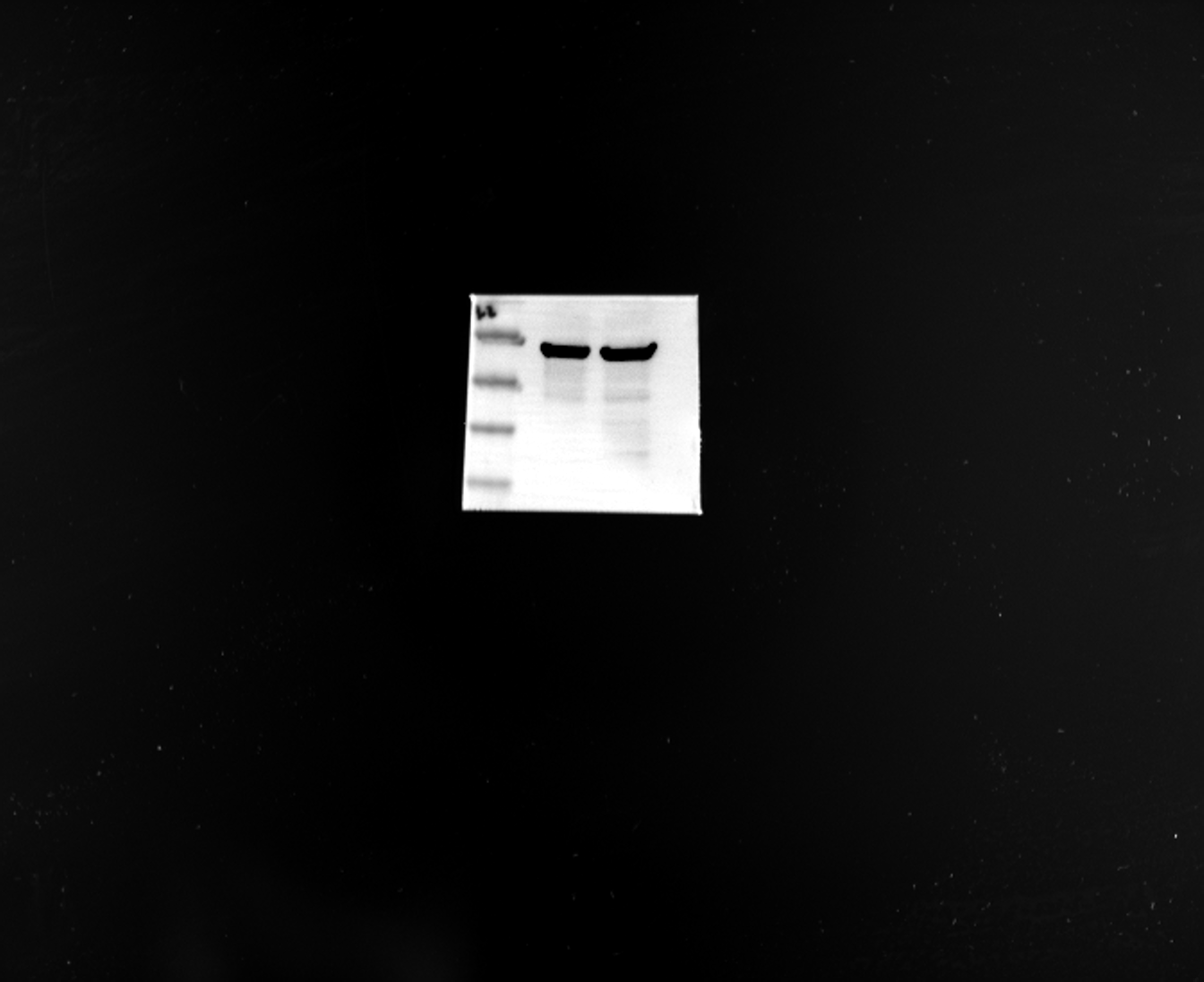

Supplement: Supplementary file 8 [file DataSheet8.zip › raw data traces-Figure 6D/p-STAT3/tubulin.Tif]

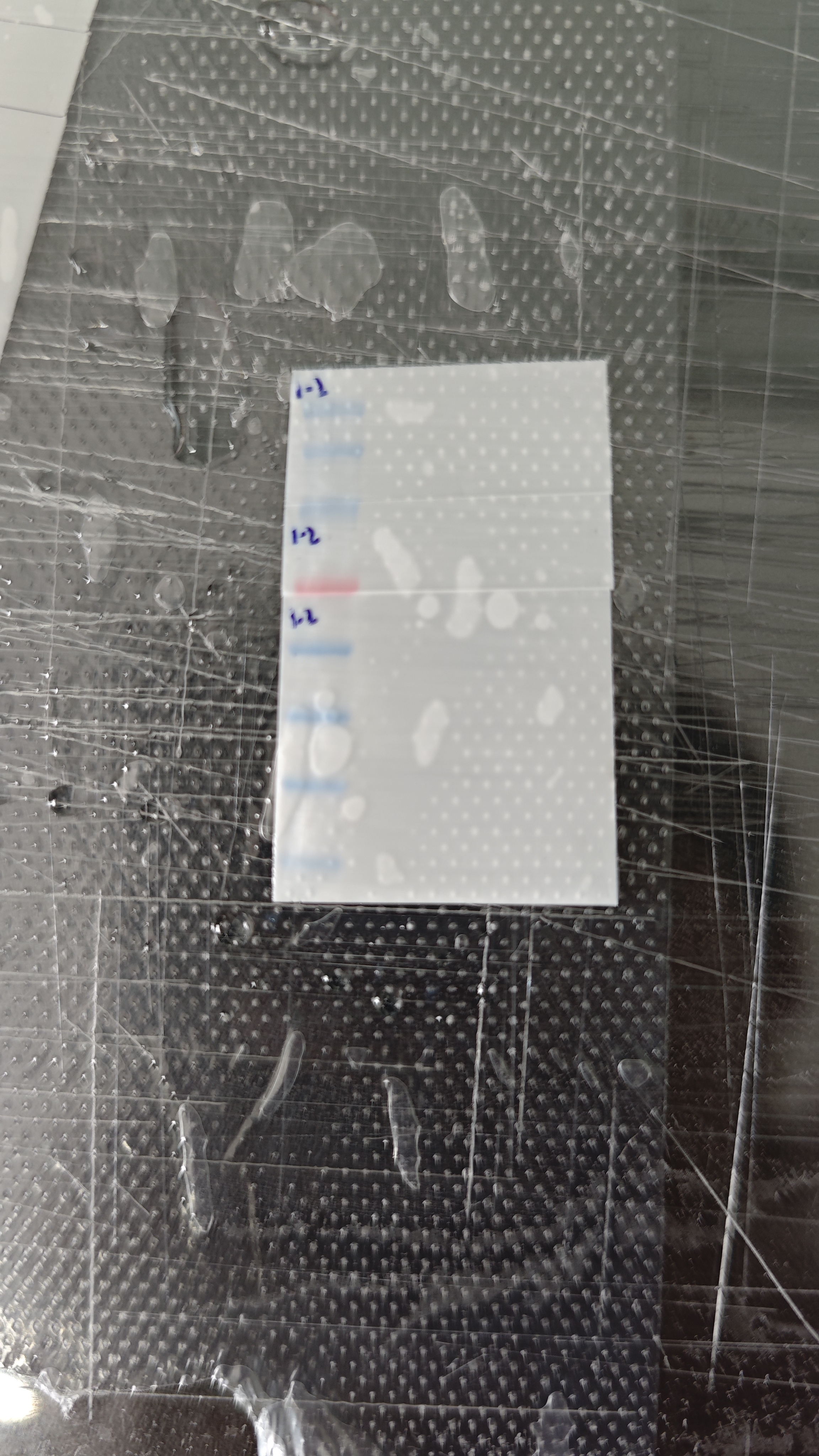

Supplement: Supplementary file 8 [file DataSheet8.zip › raw data traces-Figure 6D/p-STAT3/Uncropped images of blots.jpg]

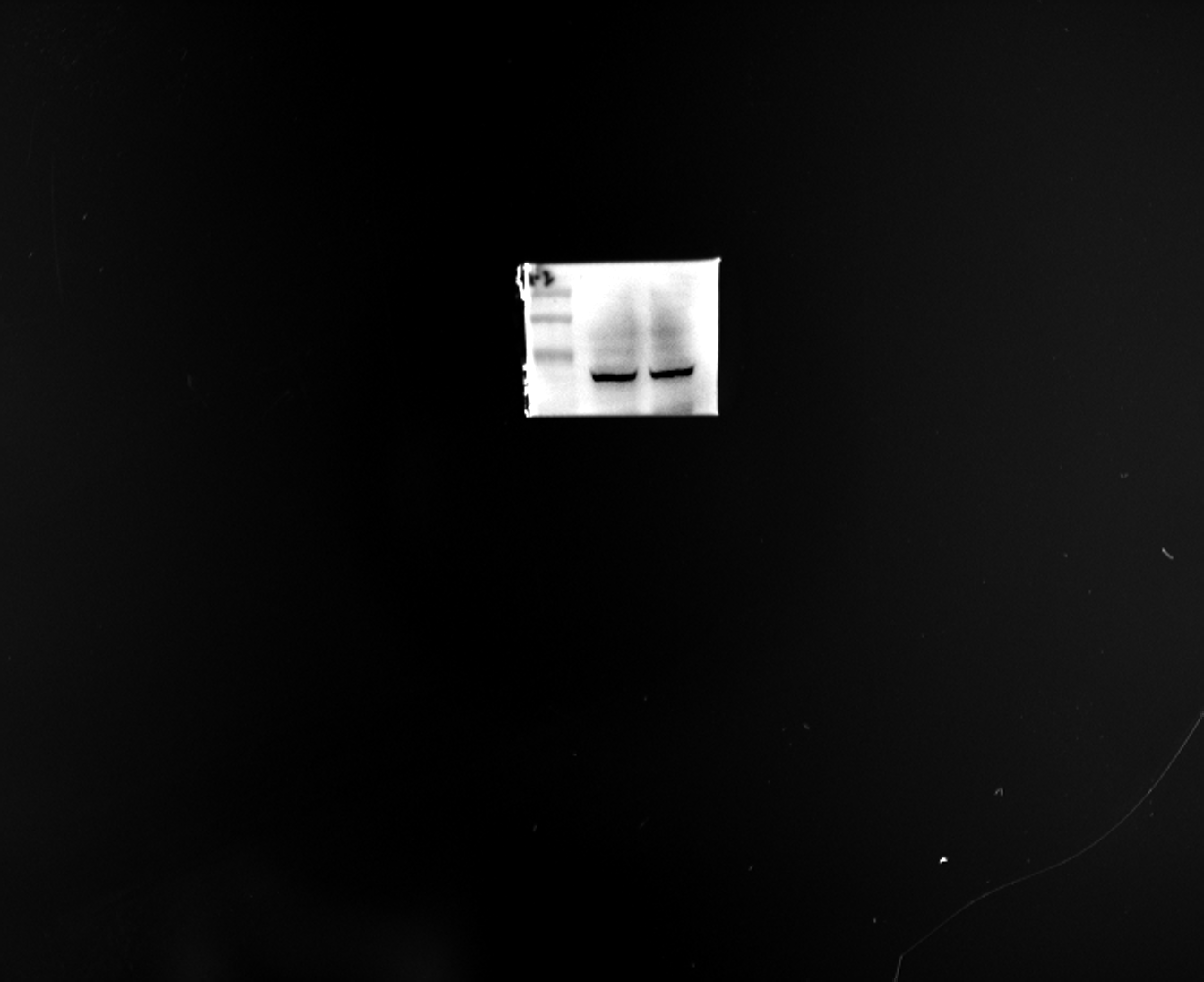

Supplement: Supplementary file 8 [file DataSheet8.zip › raw data traces-Figure 6D/STAT3/1-2T3.Tif]

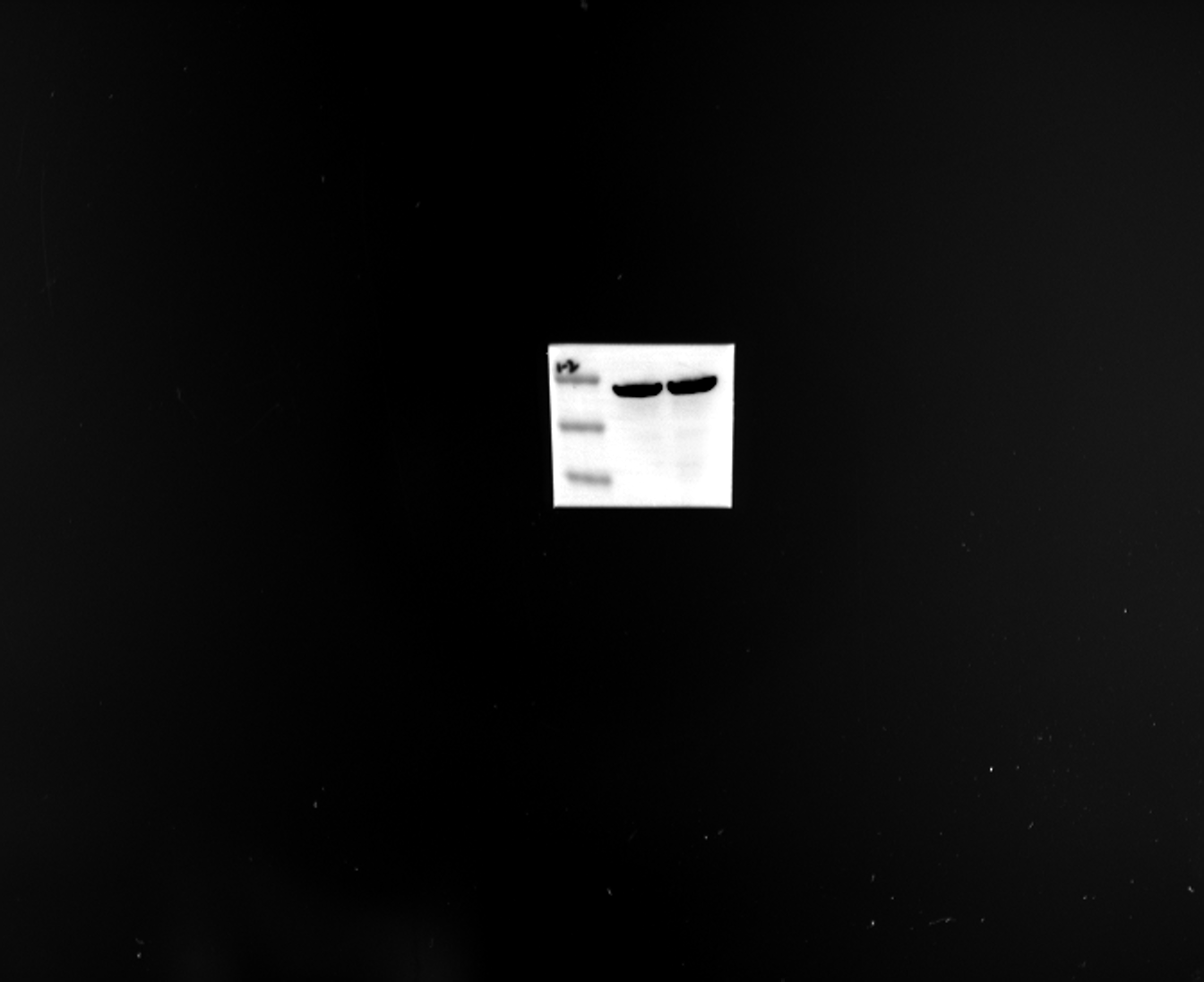

Supplement: Supplementary file 8 [file DataSheet8.zip › raw data traces-Figure 6D/STAT3/tubulin.Tif]

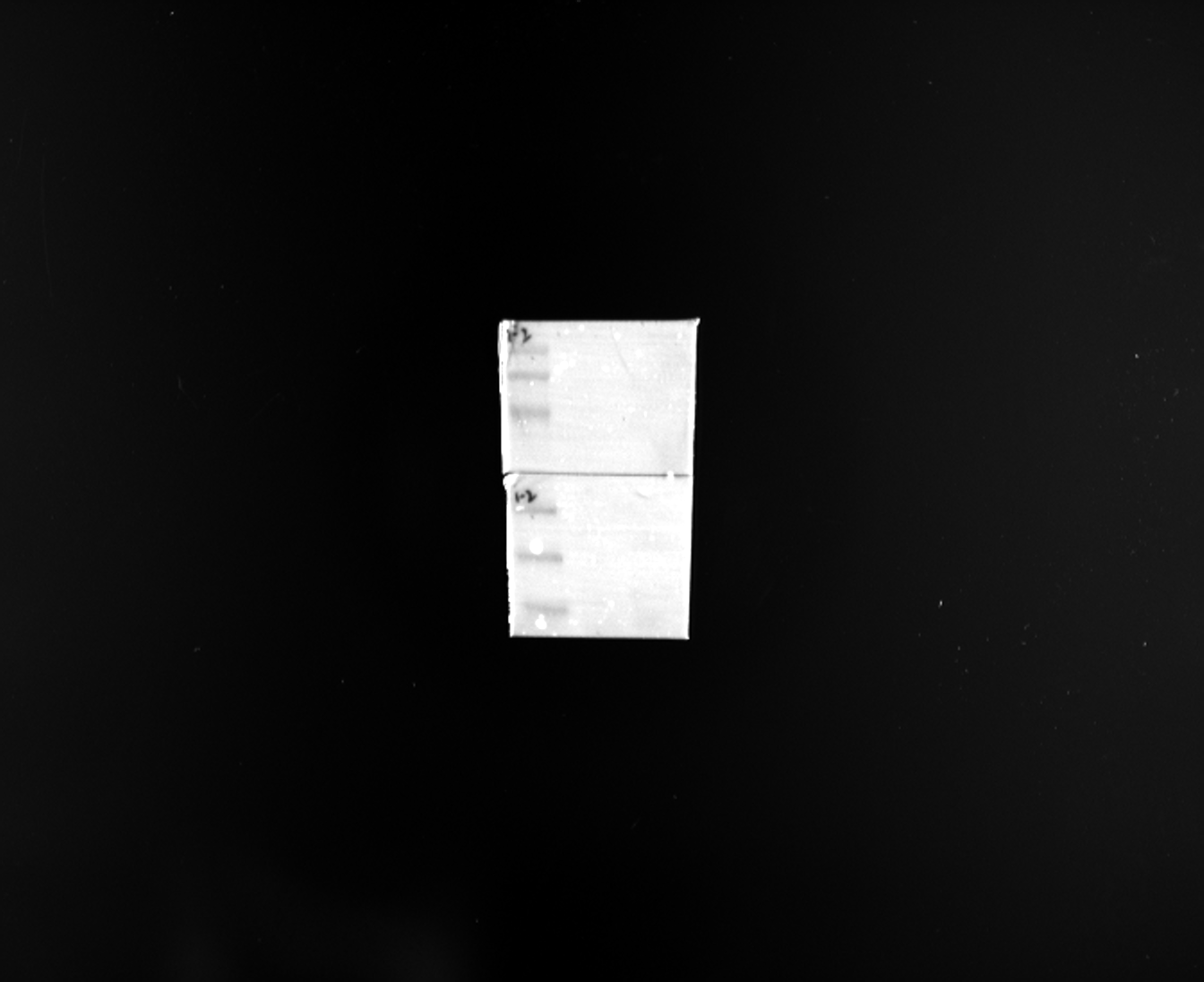

Supplement: Supplementary file 8 [file DataSheet8.zip › raw data traces-Figure 6D/STAT3/Uncropped images of blots.Tif]

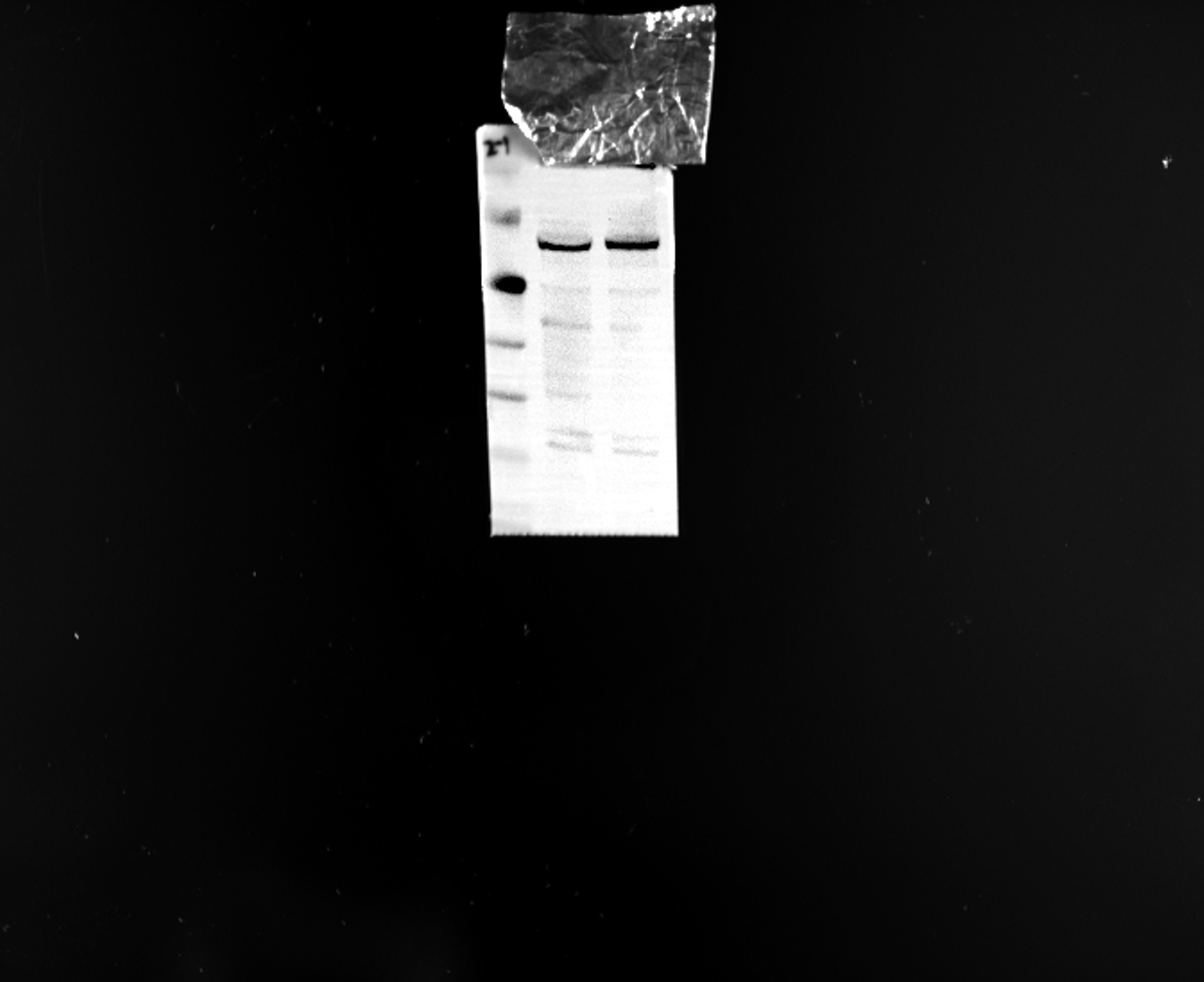

Supplement: Supplementary file 8 [file DataSheet8.zip › raw data traces-Figure 6D/TLR2/TLR2.Tif]

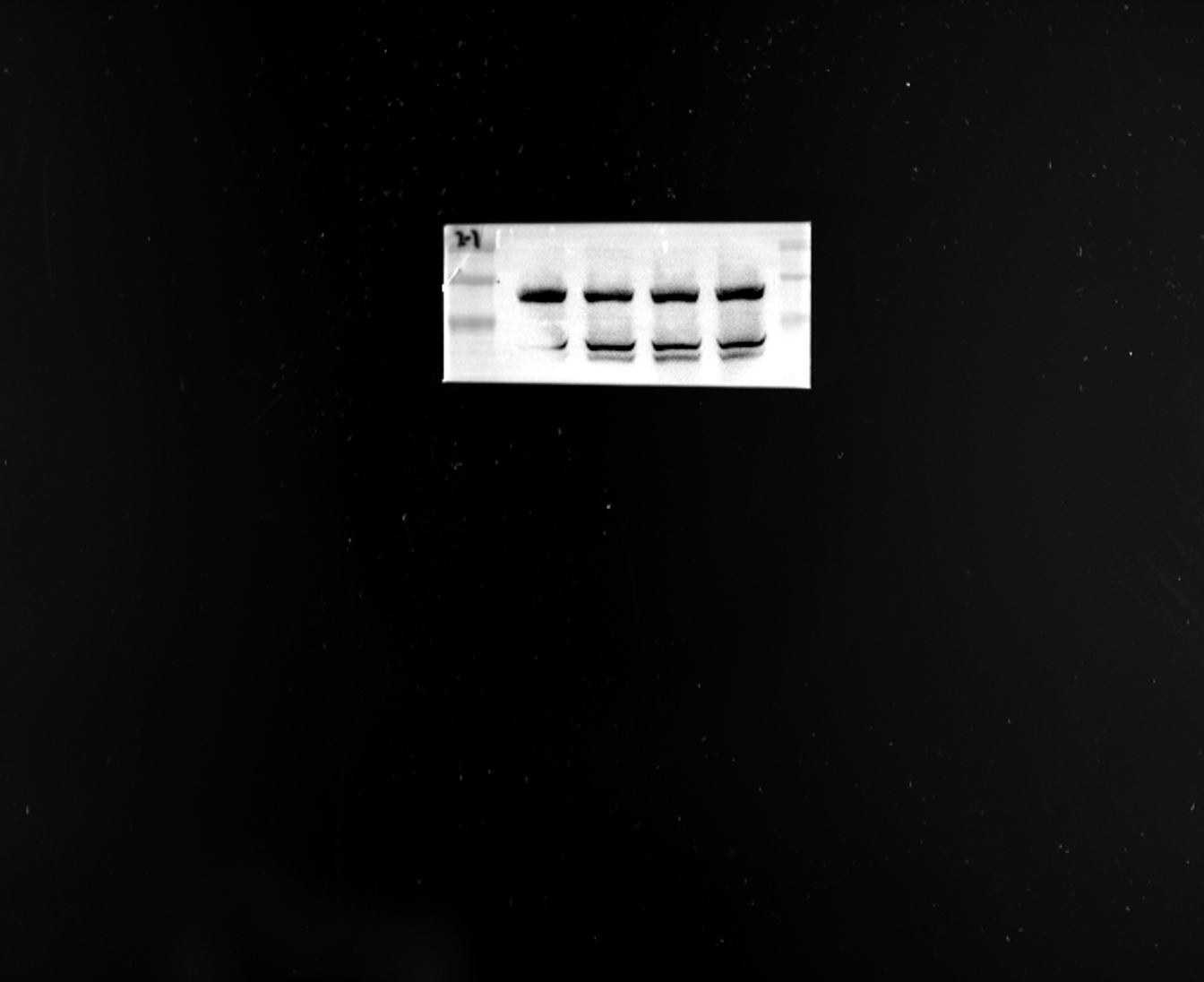

Supplement: Supplementary file 9 [file DataSheet9.zip › raw data traces-Figure 6E/JAK2/JAK2.Tif]

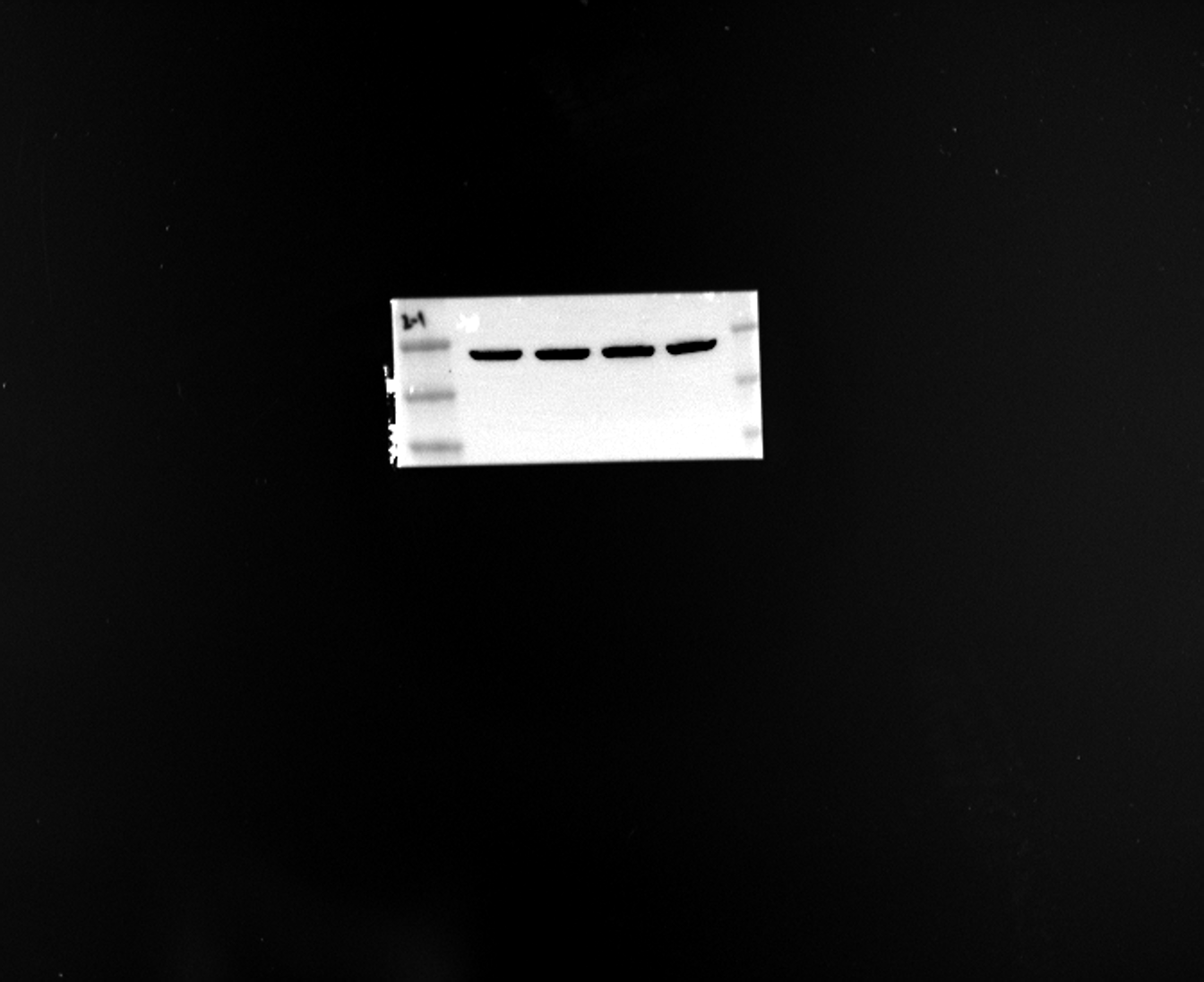

Supplement: Supplementary file 9 [file DataSheet9.zip › raw data traces-Figure 6E/JAK2/tubulin.Tif]

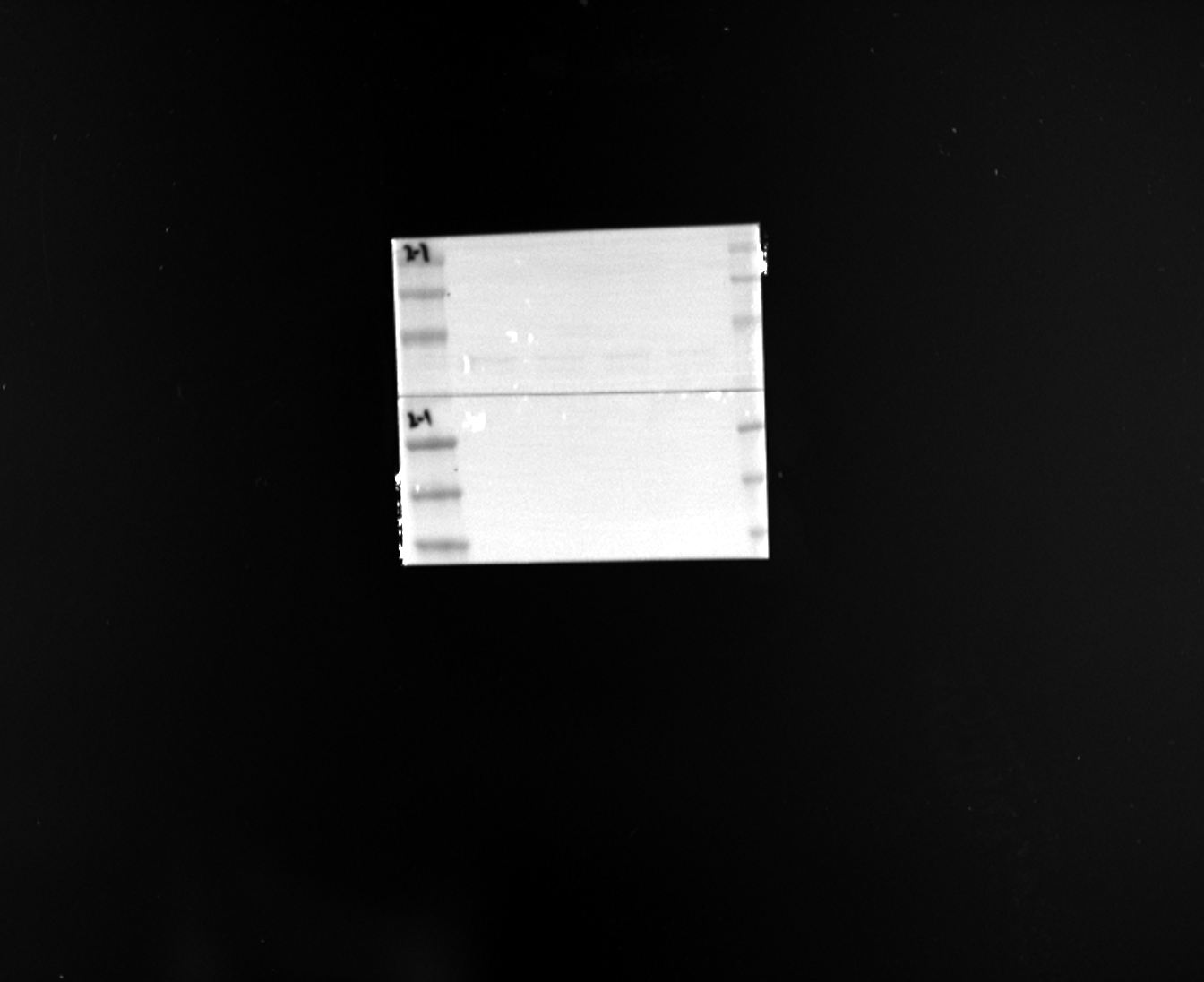

Supplement: Supplementary file 9 [file DataSheet9.zip › raw data traces-Figure 6E/JAK2/Uncropped images of blots.Tif]

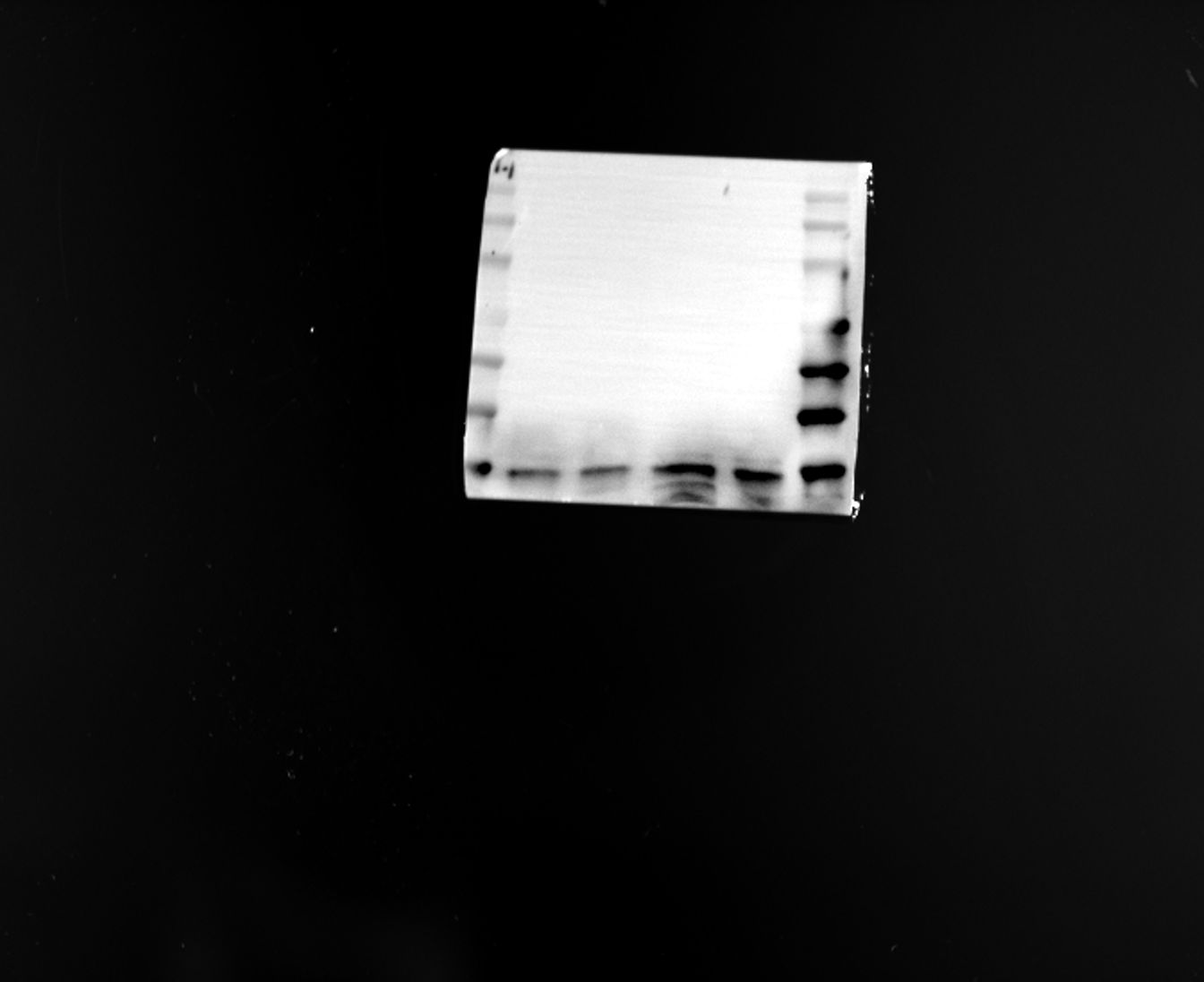

Supplement: Supplementary file 9 [file DataSheet9.zip › raw data traces-Figure 6E/MyD88/MyD88.Tif]

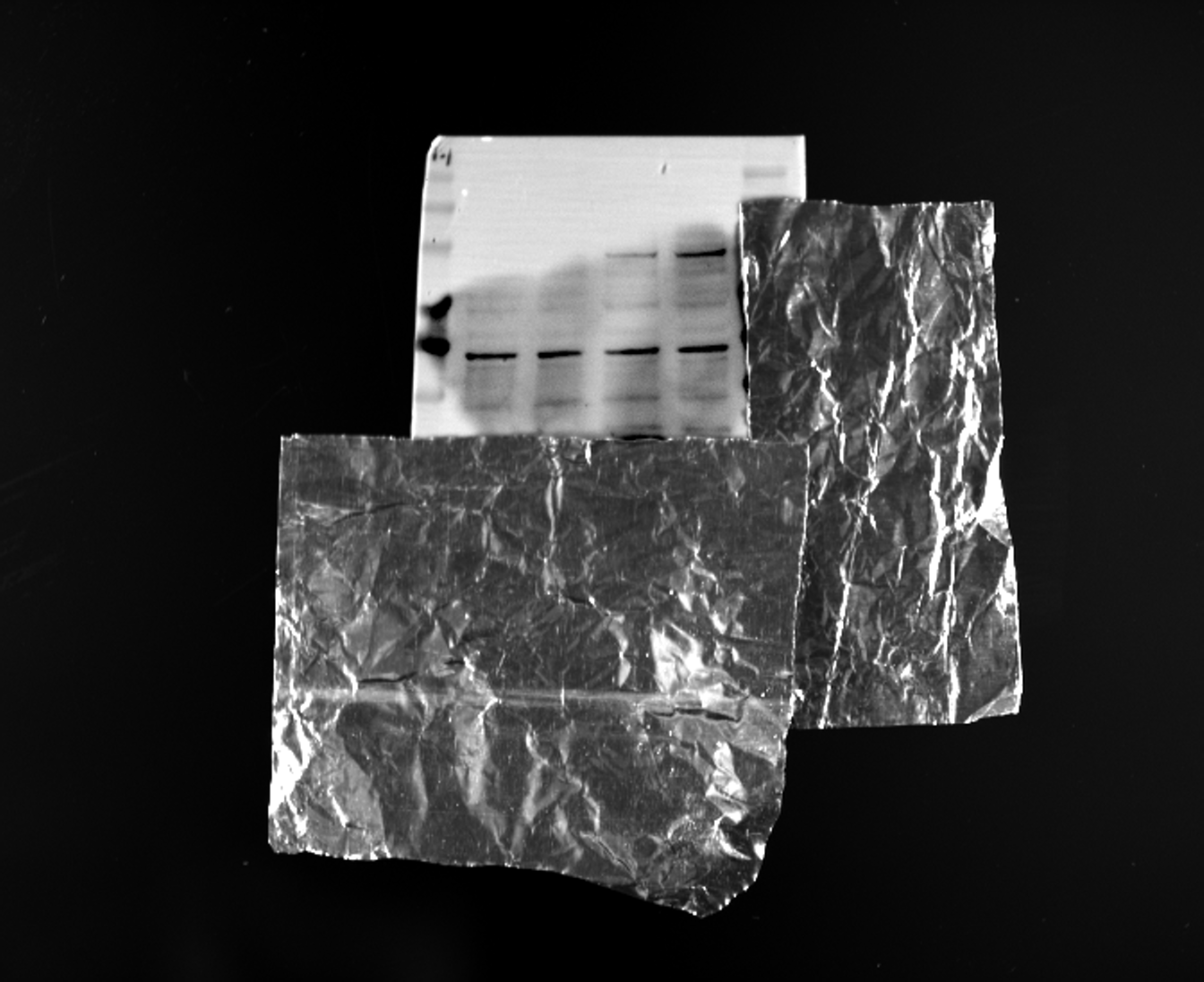

Supplement: Supplementary file 9 [file DataSheet9.zip › raw data traces-Figure 6E/MyD88/tubulin.Tif]

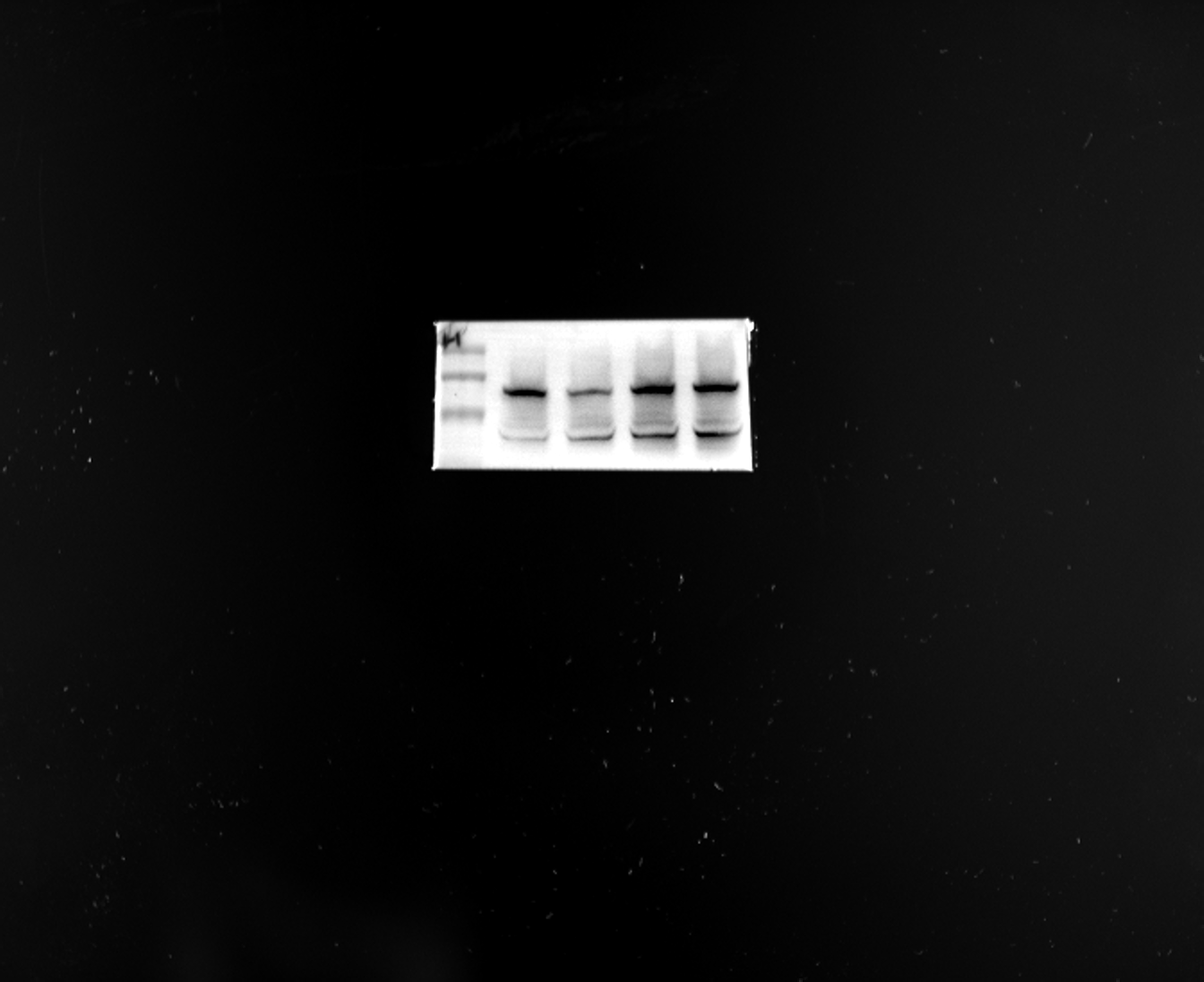

Supplement: Supplementary file 9 [file DataSheet9.zip › raw data traces-Figure 6E/p-JAK2/p-JAK2.Tif]

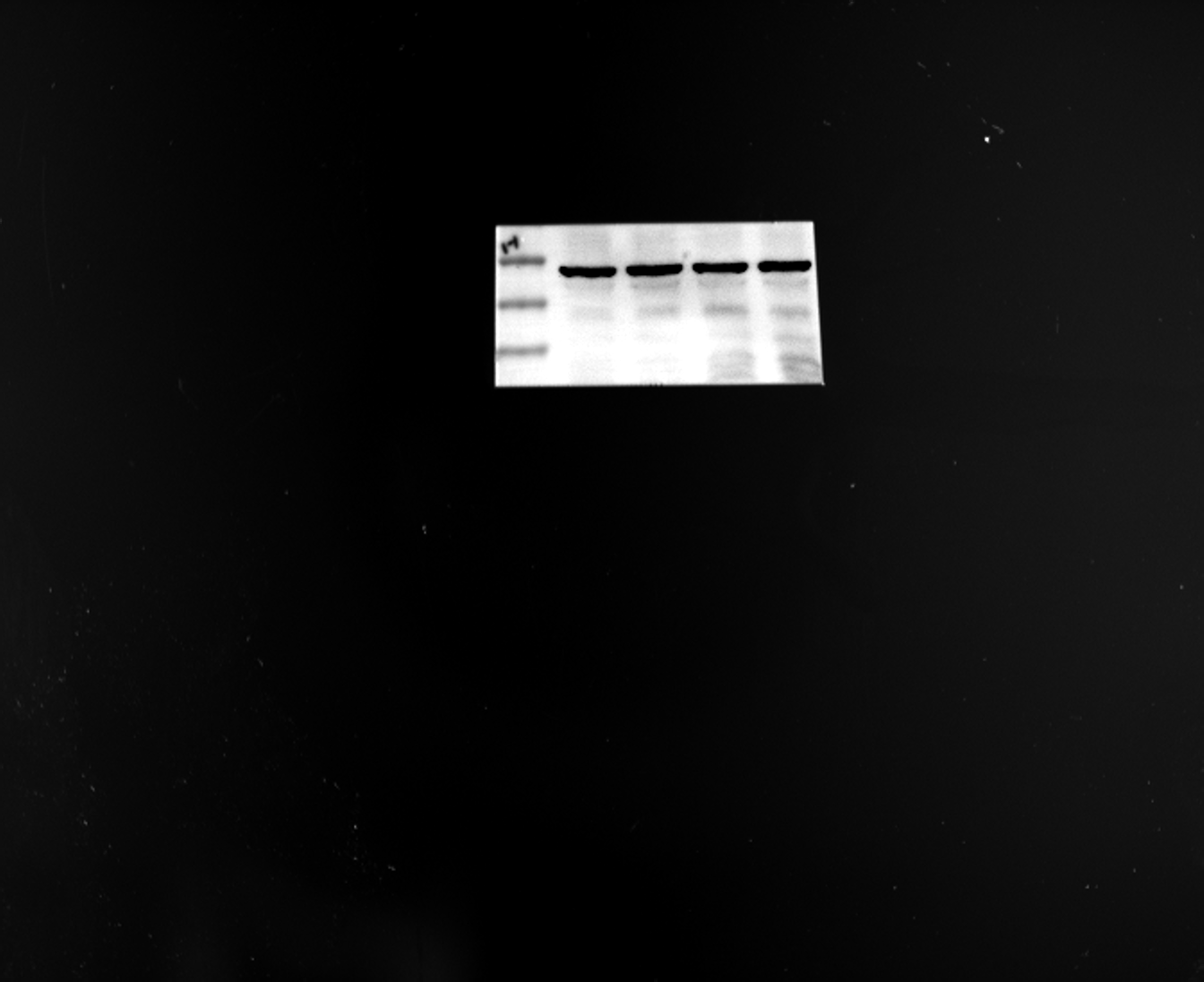

Supplement: Supplementary file 9 [file DataSheet9.zip › raw data traces-Figure 6E/p-JAK2/tubulin.Tif]

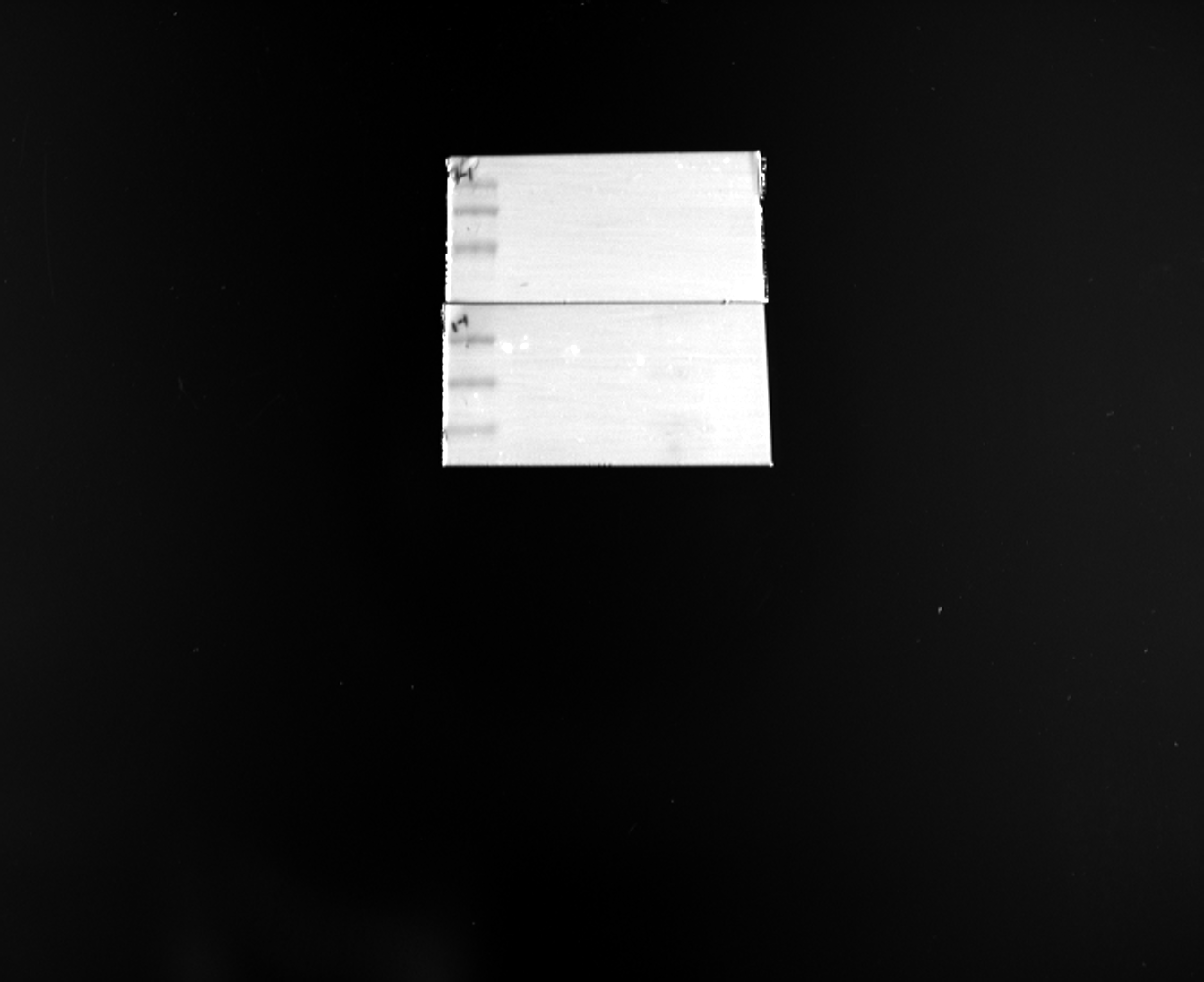

Supplement: Supplementary file 9 [file DataSheet9.zip › raw data traces-Figure 6E/p-JAK2/Uncropped images of blots.Tif]

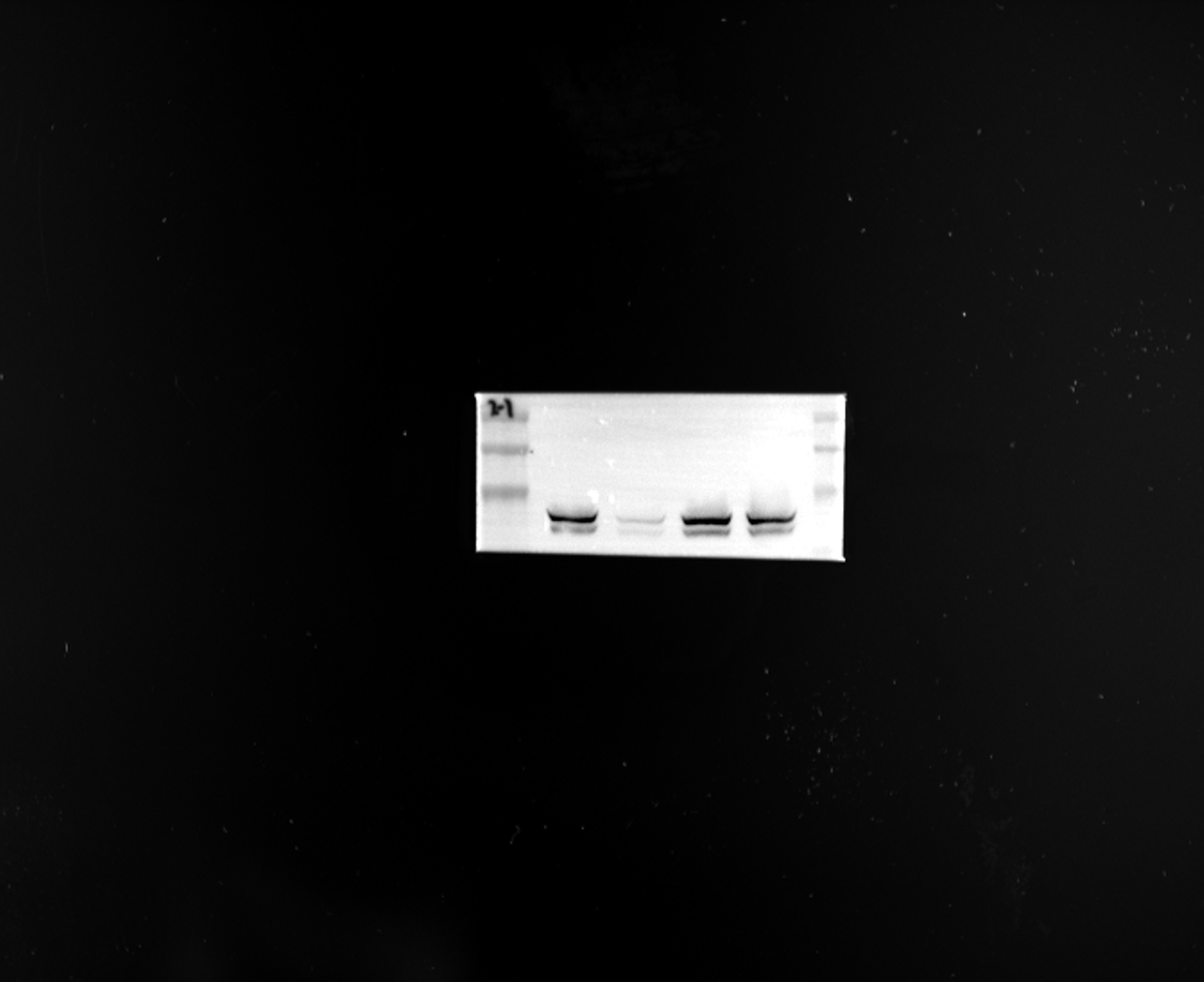

Supplement: Supplementary file 9 [file DataSheet9.zip › raw data traces-Figure 6E/p-STAT3/p-STAT3.Tif]

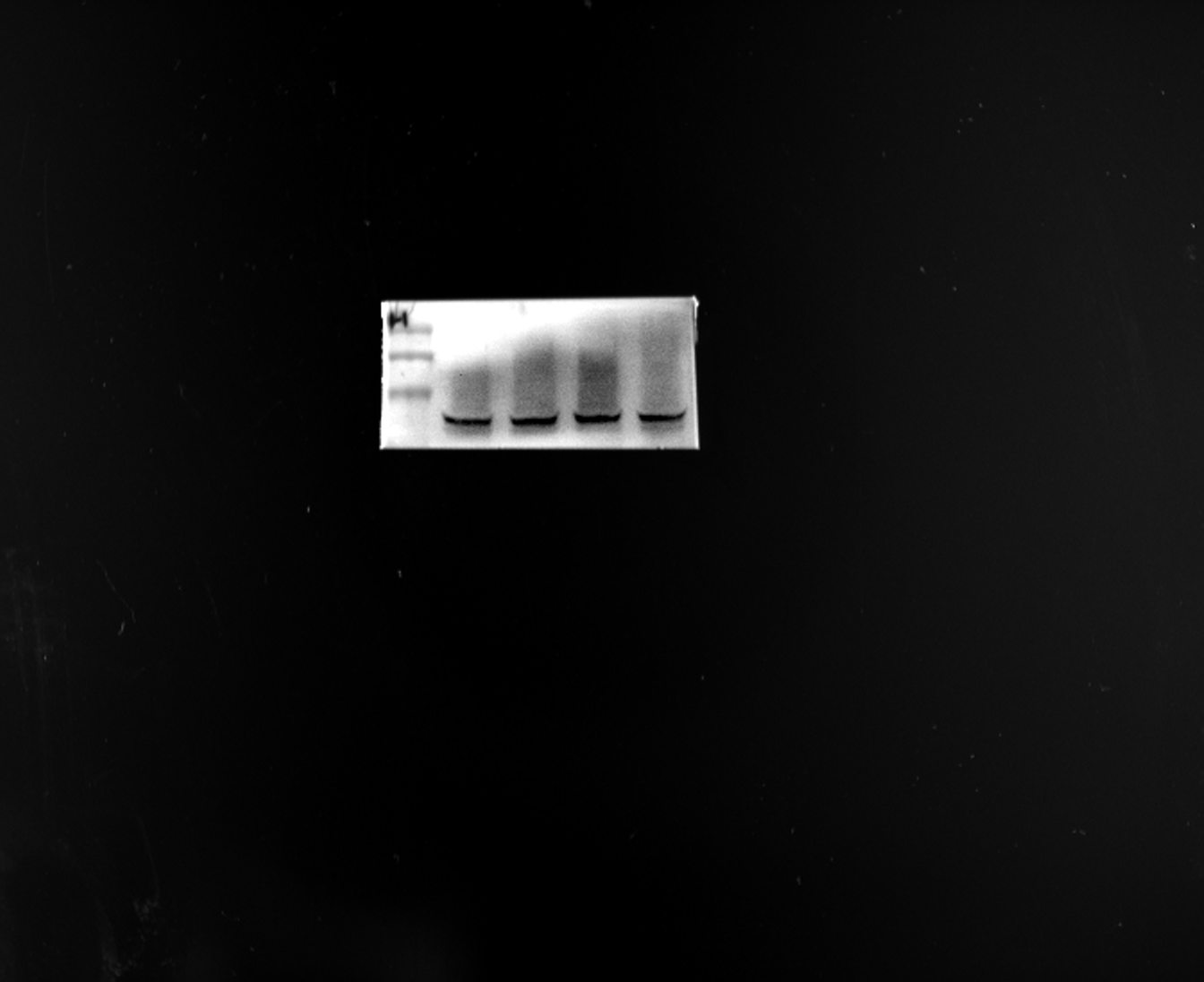

Supplement: Supplementary file 9 [file DataSheet9.zip › raw data traces-Figure 6E/STAT3/STAT3.Tif]

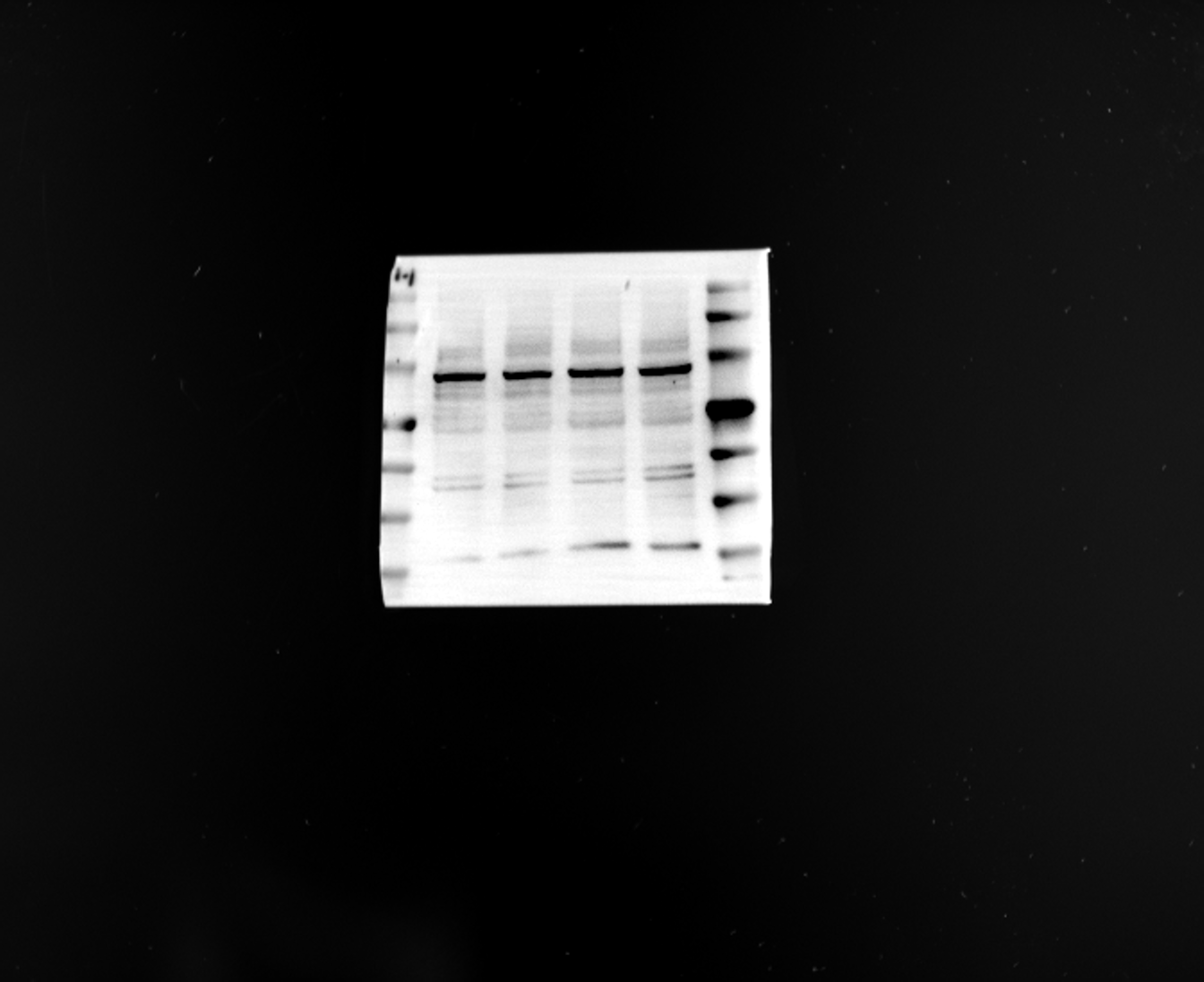

Supplement: Supplementary file 9 [file DataSheet9.zip › raw data traces-Figure 6E/TLR2/TLR2.Tif]

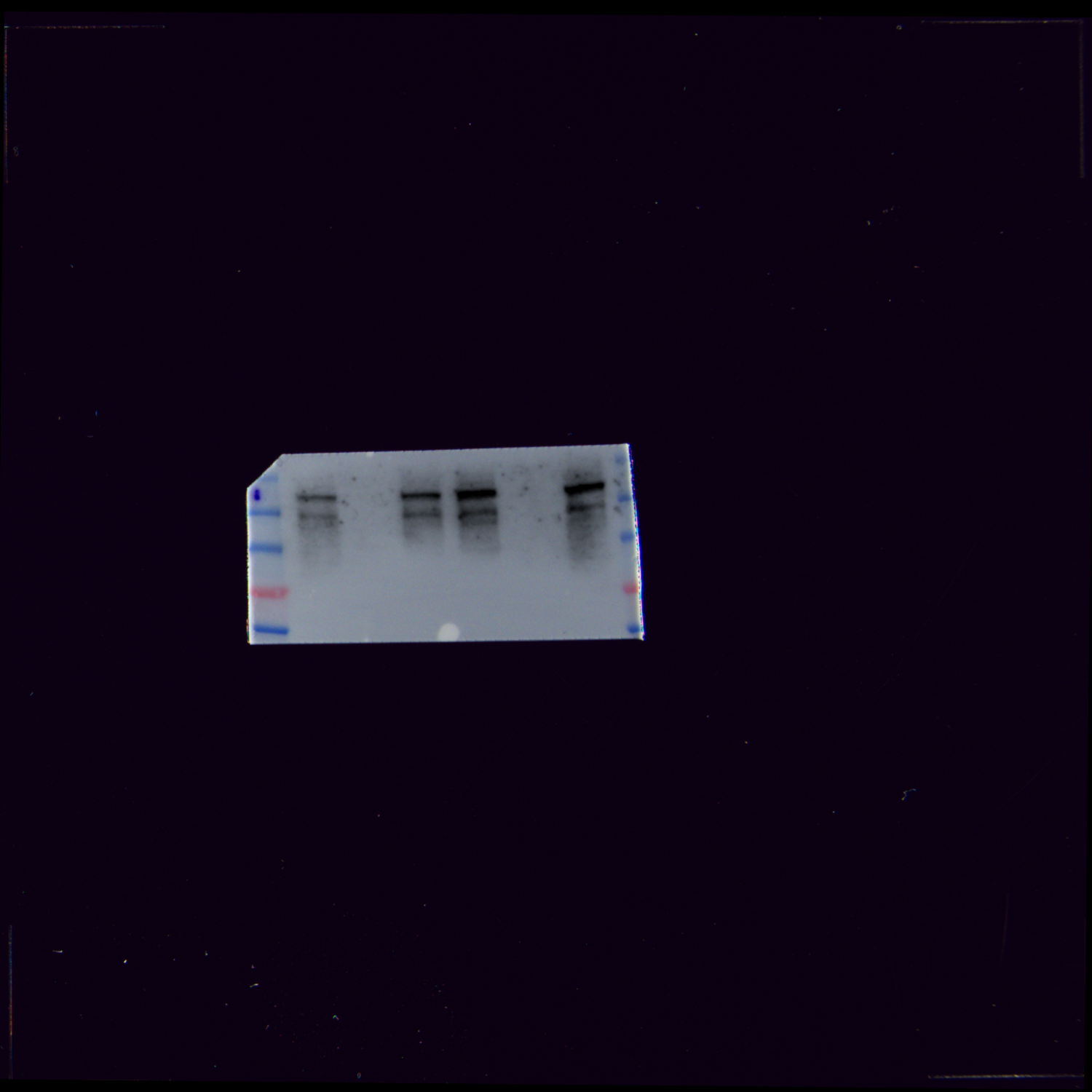

Supplement: Supplementary file 10 [file DataSheet10.zip › raw data traces-Figure 6I/Flag-JAK2.tiff]

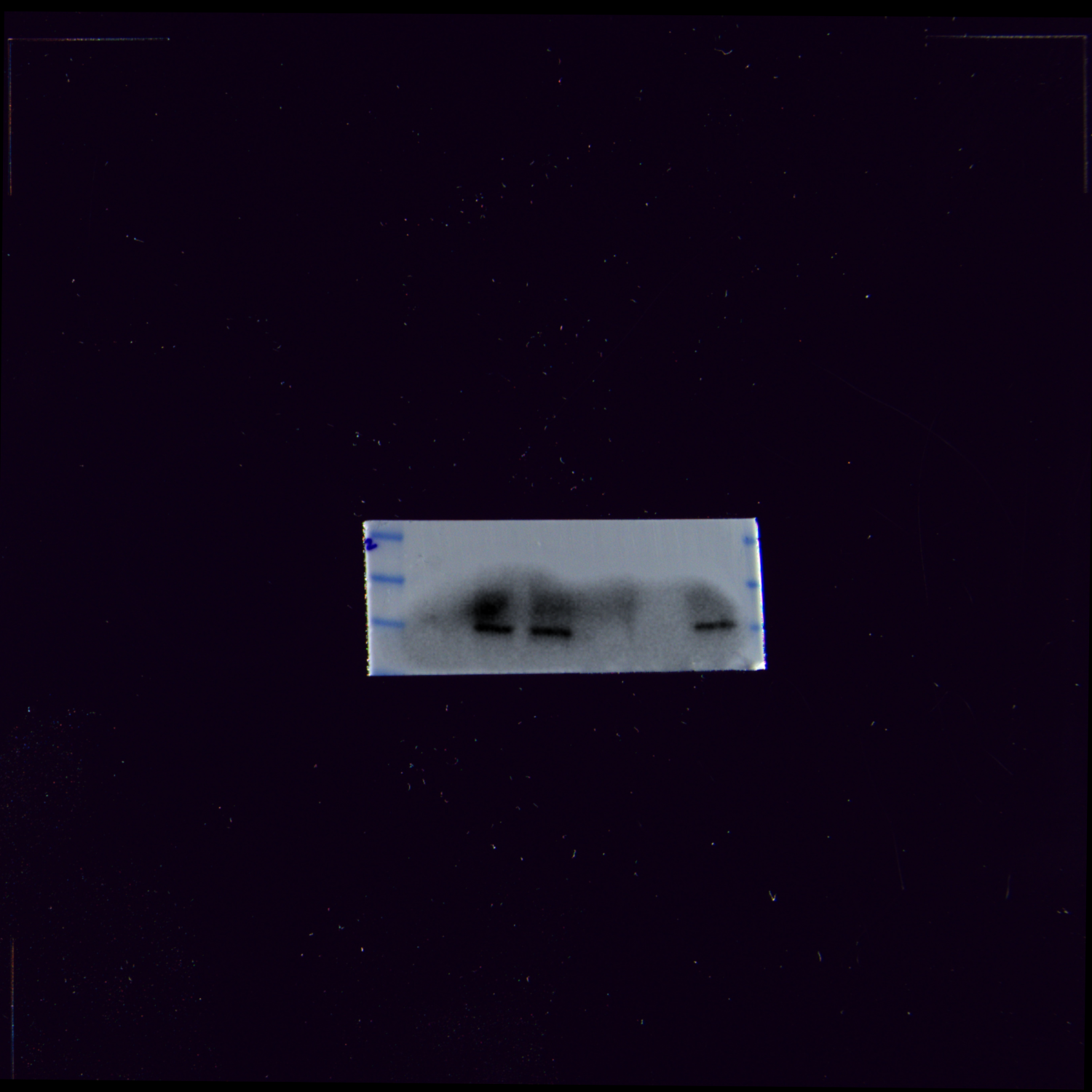

Supplement: Supplementary file 10 [file DataSheet10.zip › raw data traces-Figure 6I/HA-MyD88.tiff]

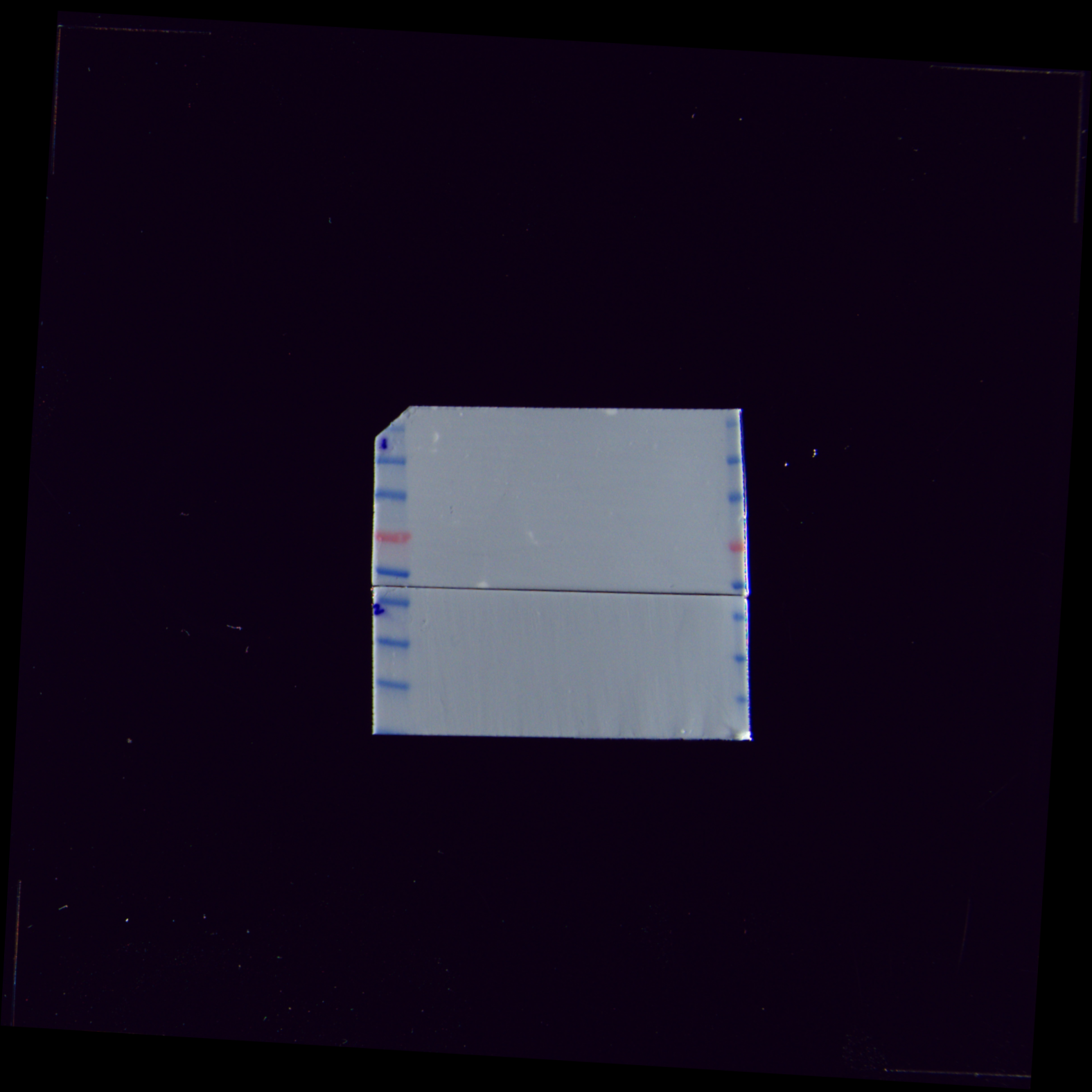

Supplement: Supplementary file 10 [file DataSheet10.zip › raw data traces-Figure 6I/Uncropped images of blots.tiff]
